# B7–H3 regulates osteoclast differentiation via type I interferon-dependent IDO induction

**DOI:** 10.1038/s41419-021-04275-6

**Published:** 2021-10-20

**Authors:** Younseo Oh, Robin Park, So Yeon Kim, Sung-ho Park, Sungsin Jo, Tae-Hwan Kim, Jong Dae Ji

**Affiliations:** 1 Hanyang University Institute for Rheumatology Research, Seoul, Republic of Korea; 2 Rheumatology, College of Medicine, Korea University, Seoul, South Korea; 3 MetroWest Medical Center/Tufts University School of Medicine, Framingham, MA 01702 USA; 4 Department of Translational Medicine, Graduate School of Biomedical Science and Engineering, Hanyang University, Seoul, Korea; 5 School of Life Sciences, Ulsan National Institute of Science & Technology (UNIST), Ulsan, 44919 Korea; 6 Department of Rheumatology, Hanyang University Hospital for Rheumatic Diseases, Seoul, Republic of Korea

**Keywords:** Checkpoint signalling, Rheumatoid arthritis, Chronic inflammation

## Abstract

While their function, as immune checkpoint molecules, is well known, B7-family proteins also function as regulatory molecules in bone remodeling. B7–H3 is a receptor ligand of the B7 family that functions primarily as a negative immune checkpoint. While the regulatory function of B7–H3 in osteoblast differentiation has been established, its role in osteoclast differentiation remains unclear. Here we show that B7–H3 is highly expressed in mature osteoclasts and that B7–H3 deficiency leads to the inhibition of osteoclastogenesis in human osteoclast precursors (OCPs). High-throughput transcriptomic analyses reveal that B7–H3 inhibition upregulates IFN signaling as well as IFN-inducible genes, including IDO. Pharmacological inhibition of type-I IFN and IDO knockdown leads to reversal of B7–H3-deficiency-mediated osteoclastogenesis suppression. Although synovial-fluid macrophages from rheumatoid-arthritis patients express B7–H3, inhibition of B7–H3 does not affect their osteoclastogenesis. Thus, our findings highlight B7–H3 as a physiologic positive regulator of osteoclast differentiation and implicate type-I IFN–IDO signaling as its downstream mechanism.

## Introduction

Osteoclasts are bone-resorptive cells derived from hematopoietic cells of the myeloid lineage that are central to physiological and pathological bone remodeling [1, 2]. The differentiation of OCPs into mature osteoclasts is triggered by stimulation with macrophage-colony-stimulating factor (M-CSF) and receptor activator of nuclear-factor kappa-B ligand (RANKL) [3]. Physiologically, osteoclast-mediated bone resorption and osteoblast-mediated bone formation maintain homeostasis [4]. On the other hand, in the pathological setting, increased osteoclast activity drives inflammatory bone destruction characteristic of certain chronic autoimmune diseases such as rheumatoid arthritis (RA) [5, 6]. Consequently, osteoclasts must be tightly regulated to suppress bone destruction and hence represent a crucial therapeutic target in inflammatory bone disorders.

The B7 superfamily of proteins comprises immunoregulatory receptor ligands that act as either costimulatory or co-inhibitory immune checkpoints by primarily regulating the activity of T cells [7]. Among them, B7–H3 is a type-I transmembrane protein, which exists in two separate isoforms that are determined by its extracellular domain. In humans, the extracellular domain consists of either one pair (2Ig–B7–H3) or two identical pairs (4Ig–B7–H3). While both isoforms are found in the intracellular compartment, only the 4Ig–B7–H3 isoform is expressed on the cell surface in human mononuclear cells [8]. The function of B7–H3 was previously thought to be a costimulatory checkpoint molecule but it has now been established as primarily a co-inhibitory immune-checkpoint molecule. Furthermore, B7–H3 is abundantly expressed in the synovium of RA patients with relative overexpression in synovial macrophages compared with their peripheral monocytes. Moreover, its expression is closely linked to clinically relevant disease-activity markers, such as C-reactive protein, erythrocyte-sedimentation rate, and Disease Activity Score-28 (DAS28), suggesting that B7–H3 is implicated in RA pathogenesis [9–11].

Certain members of the B7 family such as B7.1/B7.2 (CD80/86) and B7–H2 (ICOS ligand) act as regulatory molecules in osteoclast differentiation. Unlike in their function as immune checkpoints in which they utilize a forward-signaling mechanism to propagate their effects, B7.1/B7.2 (CD80/86) and B7–H2 (ICOS ligand) inhibit osteoclast differentiation using a distinct reverse-signaling mechanism [12, 13]. Furthermore, the dual role of immune response and the regulation of bone remodeling has also been established for B7–H3, which has been implicated in the regulation of osteoblast differentiation [14]. However, whether B7–H3 is implicated in the regulation of osteoclast differentiation remains unknown. Therefore, we aimed to elucidate the function of B7–H3 and establish B7–H3 as a regulatory mechanism in osteoclast differentiation in RA.

## Materials/subjects and methods

### Materials

Recombinant human M-CSF and soluble RANKL were purchased from PeproTech, NJ, USA. Recombinant human M-CSF receptor/Fc chimera and B7–H3–Fc were respectively obtained from Sino Biological, PA, USA and R&D System, MN, USA. Human IgG purchased from Millipore was used as the control for B7–H3–Fc. Human IFN-αR2-receptor antibody was obtained from PBL Assay Science. Human IFN-γ antibody, IL-27 antibody, and recombinant human IFN-β, IFN-γ, and IL-27 were obtained from R&D System. Mouse IgG2a purchased from R&D system was used as the isotype control. 1-D-MT, 1-L/D-MT, and CH223191 were purchased from Sigma, MA, USA.

### Cell isolation

Human peripheral blood monocytes (PBMCs) were isolated from the blood of healthy donors using Ficoll gradient centrifugation (GE Healthcare, IL, USA). Monocytes were purified from PBMCs using CD14-positive magnetic beads (Miltenyi Biotec, CA, USA) according to the manufacturer’s instructions. Inflammatory RA or ankylosing-spondylitis (AS) macrophages were purified by positive selection using CD14 magnetic beads from synovial-fluid mononuclear cells. The experiments using human cells were approved by the Ethics Committee of Hanyang University Hospital for Rheumatic Disease (IRB No. 2008-09-001, 2017-05-003). All healthy volunteers and RA and AS patients provided their written informed consent to participate in this study.

### Mice

Wild-type (WT) and B7–H3-knockout (KO) (B7–H3^−/−^) mice with the C57BL/6 background were purchased from Cyagen Biosciences. Eight-week-old mice were housed in a specific pathogen-free facility. All experiments were approved by the Institutional Animal Care and Use Committee at Hanyang University (HY-IACUC-2020-0131).

### Osteoclast differentiation

For human osteoclast differentiation, monocytes of human PBMC origin were incubated with 20 ng/ml M-CSF for two days to derive OCPs, which were further cultured in 20 ng/ml M-CSF and 40 ng/ml RANKL for an additional six days. For mouse osteoclast differentiation, whole bone marrow cells were isolated from femurs and tibias of mice. The cells were cultured in 10 ng/ml M-CSF for one day. Nonadherent cells were then cultured with 20 ng/ml M-CSF for one day to obtain bone marrow-derived macrophages (BMMs). The attached cells, which are capable of osteoclast differentiation, were seeded and cultured with 50 ng/ml M-CSF and 100 ng/ml RANKL for four days. Cytokines were replenished every three days. The cells were cultured in α-MEM (Gibco, MT, USA) supplemented with 10% FBS (Gibco, MT, USA). The cells were fixed with 4% paraformaldehyde and stained for TRAP using Acid Phosphatase Leukocytes diagnostic kit (Cosmo Bio, CA, USA), as recommended by the manufacturer. Multinucleated (>3 or 10 nuclei in human osteoclasts, >10 nuclei in mouse osteoclasts) TRAP-positive cells were counted in duplicate wells of 48-well plates. Microphotographs of staining were observed at original magnification X100.

### Transfection

Before transfection, 40 ng/ml M-CSF was added to CD14-positive cells from PBMCs or RA synovial-fluid mononuclear cells for three days. For RNA interference, the OCPs were transfected with ON-TARGET plus SMARTpool siRNAs specific for B7–H3, IDO, and AhR (Dharmacon). ON-TARGET plus Non-targeting Pool was used as the control. To overexpress *B7–H3*, the OCPs were transfected with human pCMV3–B7–H3 ORF-expression plasmid and pCMV3-negative control vector (Sino Biological, PA, USA). For transfection, lipofectamine 3000 (Invitrogen, CA, USA) was used according to the manufacturer’s instructions. After siRNA or plasmid transfection, OCPs were cultured in α-MEM with 40 ng/ml M-CSF and 80 ng/ml RANKL for seven days to generate mature osteoclasts. Of note, M-CSF 20 ng/mL was used as the default concentration, while the 40 ng/mL concentration was used to increase the transfection efficiency.

### Actin-ring staining

At the end of the culture period, the cells were fixed with 4% paraformaldehyde, permeabilized in 0.1% Triton X-100 for 5 min, and then stained with FITC-conjugated phalloidin (Sigma-Aldrich) for 1 h at 37 °C.

### Bone-resorption assays

After siRNA transfection, OCPs were plated on dentin slices (Immunodiagnostic Systems, UK) in 96-well plates and cultured with M-CSF and RANKL. The cells on the dentin disc were removed by washing with 70% ethanol. The dentin discs were rinsed with water and immersed in 1% toluidine blue O (Sigma-Aldrich) to stain resorption pits formed by osteoclasts.

### Bone-density imaging and analysis

Femurs from WT and B7–H3 KO male mice were fixed in 4% paraformaldehyde for micro-CT analysis. Trabecular and cortical morphometry within the femur were quantified using a benchtop cone-beam-type in vivo animal scanner (SKYSCAN1172 micro-CT, Bruker microCT, Belgium). Samples were imaged with the following settings: 60 kVp, 166 μA, and an aluminum 0.25-mm-thick filter. The pixel size was 9.0 μm and the rotation step was 0.6°. The cross-sectional images were reconstructed using a filtered back-projection algorithm (NRecon software, Bruker microCT, Belgium). Trabecular morphometry used the following measurement parameters: bone-volume fraction (BV/TV), trabecular thickness (Tb. Th), trabecular number (Tb. No), and trabecular spacing (Tb. Sp).

### Quantitative real-time PCR

Total RNA was extracted from the cells using Trizol (Invitrogen) according to the manufacturer’s instructions. The RNA was reverse-transcribed using reverse transcriptase (Invitrogen) and random hexamers (Invitrogen). qPCR was performed in triplicate with Fast SYBR Green Master Mix (Bioline, London, UK) using CFX96 (Bio-Rad, CA, USA). The primers used for qPCR are provided in Supplementary Table S1.

### Immunoblotting

Total cell extracts were obtained using 1X RIPA lysis buffer (Cell Signaling, MA, USA) containing 1X proteinase-inhibitor cocktail (Calbiochem, CA, USA), 1 mM PMSF (Sigma-Aldrich), and 1X phosphatase-inhibitor (Cell Signaling, MA, USA). Cell lysates were fractionated on 10% SDS-polyacrylamide gels, transferred to nitrocellulose membranes (GE Healthcare Life Sciences), and incubated with specific primary antibodies. Immunoblotting was performed with the following antibodies: B7–H3 (R&D System); c-Fos (Abcam, MA, USA); NFATc1 (BD Biosciences, CA, USA); c-Fms, IκBα, p-p38 MAPK, p-p44/42 MAPK, p44/42 MAPK, IDO, p-STAT1, STAT1, AhR, β-actin, and GAPDH (Cell Signaling, MA, USA); RANK, p38α, and iNOS (Santa Cruz, CA, USA). Secondary antibodies (Jackson ImmunoResearch) were incubated before adding the ECL substrate (Invitrogen). For quantitative analysis of protein expression, the optical densities of the blot bands were measured using the Image J software.

### Flow cytometry

Cells were washed with 1X PBS containing 0.5% bovine serum albumin and 0.1% sodium azide and then incubated in Fc-receptor-blocking reagent (Miltenyi Biotec). Cells were stained with APC-conjugated anti-human B7–H3 (Biolegend, CA, USA) and analyzed by using FACS Canto flow cytometer (BD Biosciences, CA, USA). The flow-cytometric data were analyzed using FlowJo software (Treestar, OR, USA).

### Cell-proliferation assay

Cells were seeded in 96-well plates and added Ez-cytox viability kit reagent (DoGen, Kyoto, Japan) for the indicated number of days. The cells were incubated for 1 h at 37°C to allow the Ez-cytox viability kit reagent to metabolize to formazan. Absorbance was measured at 450 nm using a microplate reader and culture medium was used as a blank.

### RNA sequencing

Total RNA concentration was calculated by Quant-IT RiboGreen (Invitrogen). To assess the integrity of the total RNA, samples are run on the *TapeStation RNA screentape* (Agilent, CA, USA). Only high-quality RNA preparations, with RIN greater than 7.0, were used for RNA library construction. A library was independently prepared with total RNA (1ug) for each sample by TruSeq Stranded mRNA Sample Prep Kit (Illumina, CA, USA). The libraries were quantified using KAPA Library Quantification kits for Illumina Sequencing platforms according to the qPCR Quantification Protocol (Kapa Biosystems, MA, USA) and qualified using the TapeStation D1000 ScreenTape (Agilent, CA, USA). Indexed libraries were then submitted to an Illumina NovaSeq (Illumina, CA, USA), and the paired-end (2 × 100 bp) sequencing was performed by the Macrogen Incorporated. Differentially expressed genes between B7–H3-deficient and control transcriptomes were identified using DESeq2 using a p-value <0.05 as the significance threshold and enriched pathways were identified using IPA.

### Statistical analysis

The results are expressed as mean ± SD. The Mann–Whitney U test was used to calculate statistical significance. Statistical significance was defined as *P < 0.05, **P < 0.01, ***P < 0.001. Columns and error bars in figures indicate the mean values and standard errors for at least three independent experiments.

## Results

### B7–H3 is expressed during human osteoclast differentiation

**Fig. 1:**
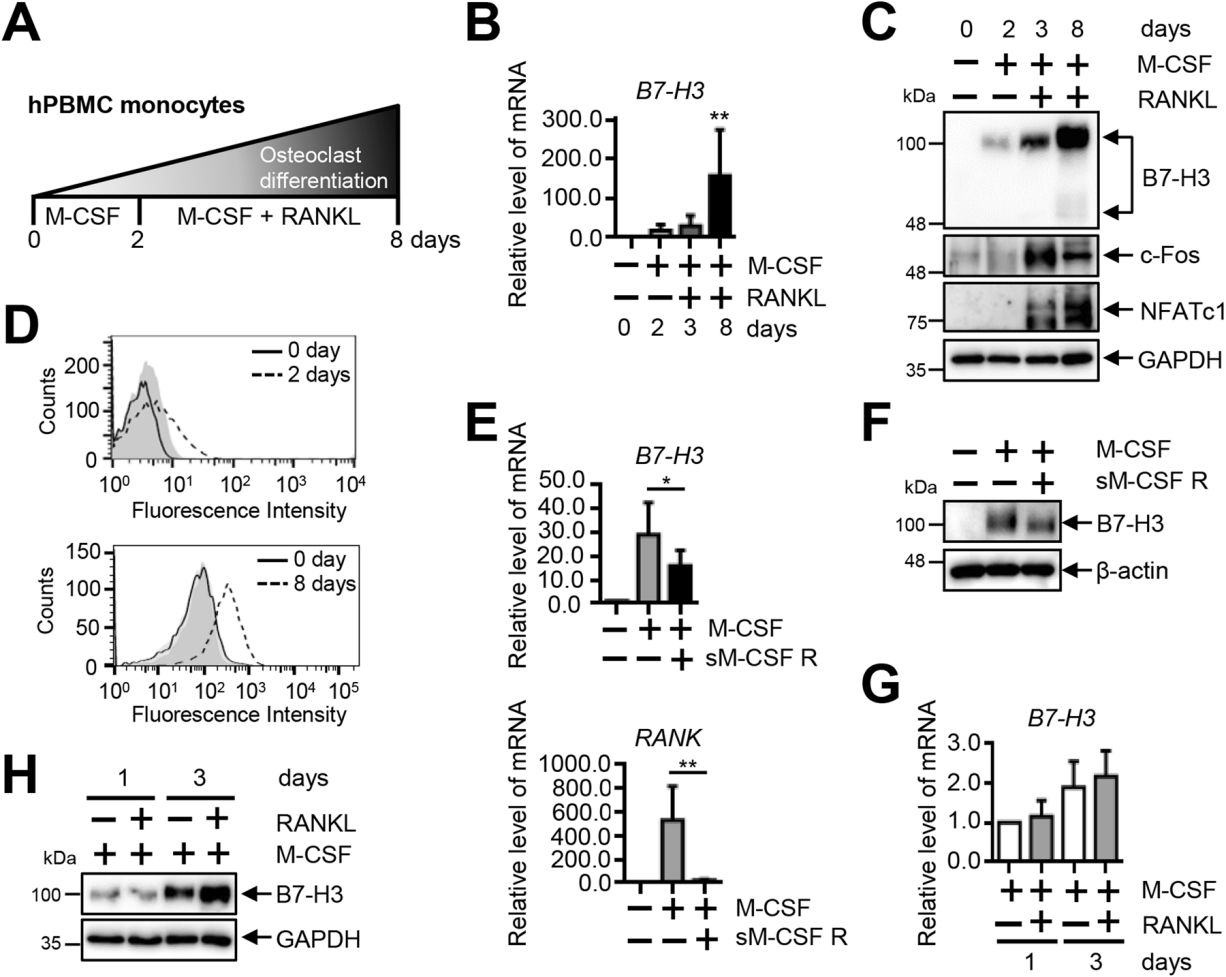
B7–H3 expression is increased during differentiation of human osteoclasts. **A** A schematic diagram outlining the process of osteoclast differentiation from human PBMCs. **B** B7–H3 mRNA expression during human osteoclast differentiation measured using RT-qPCR. **C** Whole-cell lysates were immunoblotted with B7–H3, c-Fos, and NFATc1 Abs. **D** At days 0, 2, and 8, the cell-surface B7–H3 expression was assessed by flow cytometry (solid line: monocytes; dashed line: OCPs or mature osteoclasts; gray shaded: isotype control). **E**, **F** OCPs were cultured as described in Fig. 1A. OCPs were incubated with M-CSF (20 ng/ml) in the presence or absence of recombinant human M-CSF receptor/Fc chimera (sM-CSF R) overnight. The expression of B7–H3 and RANK mRNA was measured using RT-qPCR and the expression of B7–H3 protein was detected using Western blot. **G**, **H** OCPs were cultured with M-CSF (20 ng/ml) in the presence of RANKL (40 ng/ml) for one or three days. RT-qPCR and Western blot were performed to detect the expression of B7–H3. (**B**, **E**, **G**) mRNA levels were normalized with GAPDH expression.

To confirm the presence of B7–H3 expression during human osteoclast differentiation, we induced PBMCs from healthy controls (HC) to undergo osteoclastogenesis using M-CSF and RANKL (Fig. 1A). Expression of the mRNA, protein, and cell-surface protein levels of B7–H3 gradually increased during osteoclast differentiation (Fig. 1B, C, D). Since the main form of cell-surface protein on human mononuclear cells is the 4Ig–B7–H3 isoform, our experiments involving B7–H3-surface proteins hereafter refer to this isoform. Osteoclast differentiation was confirmed using the expression of c-Fos and NFATc1 (Fig. 1C). We found that M-CSF blockade partially abolished M-CSF-induced B7–H3 mRNA and protein expression (Fig. 1E, F). Furthermore, RANKL upregulated B7–H3 protein expression at day 3 without affecting changes in its mRNA levels (Fig. 1G, H). Taken together, these findings confirm that B7–H3 expression is induced upon osteoclast differentiation.

### B7–H3 deficiency inhibits human osteoclast differentiation

**Fig. 2:**
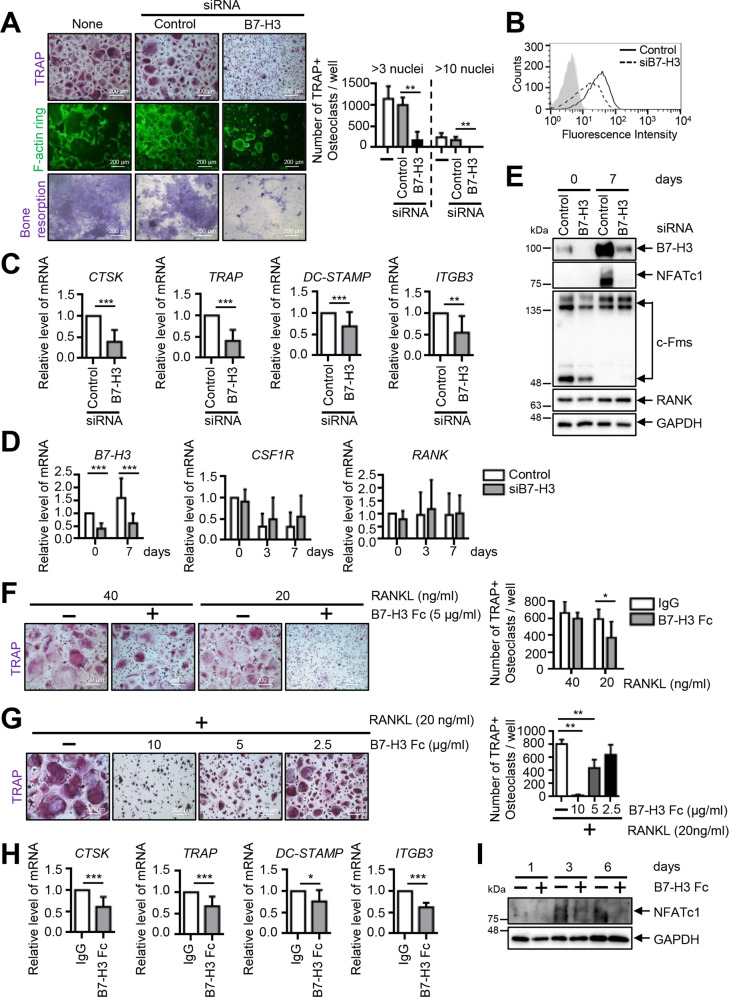
B7–H3 deficiency suppresses human osteoclast differentiation. **A** Prior to transfection, M-CSF (40 ng/ml) was added to monocyte culture for three days. OCPs were transiently transfected with B7–H3-specific siRNA, control siRNA (40 nM), or neither, and then induced to differentiate using M-CSF (40 ng/ml) and RANKL (80 ng/ml) for seven days. After staining for TRAP expression, the TRAP-positive multinucleated cells were counted as osteoclasts (>3 or 10 nuclei). Actin rings in osteoclasts were stained with FITC-phalloidin and bone-resorption pits were stained with 1% toluidine blue O in 0.5% sodium borate (scale bar, 200 μm). **B** Cell-surface B7–H3 expression was measured using flow cytometry at day 0. The representative histograms are shown (solid line: control siRNA; dashed line: B7-H3-specific siRNA; gray shaded: isotype control). **C** The expression of mature osteoclast markers, CTSK, TRAP, DC-STAMP, and ITGB3, was analyzed using RT-qPCR at day 7. **D** The expression of B7–H3, CSF1R, and RANK mRNA during osteoclast differentiation was detected using RT-qPCR. **E** Whole-cell lysates were immunoblotted with B7–H3, NFATc1, c-Fms and RANK antibodies. **F** Monocytes were cultured with M-CSF (20 ng/ml) for two days. OCPs were further incubated with M-CSF (20 ng/ml) and RANKL (40 or 20 ng/ml) in the presence of B7–H3–Fc (5 μg/ml) or human IgG for six days. **G** OCPs were cultured with M-CSF (20 ng/ml) and RANKL (20 ng/ml) in the presence of B7–H3–Fc (10, 5, and 2.5 μg/ml) or human IgG for six days. **F**, **G** TRAP staining was performed and the number of TRAP-positive multinucleated cells per well were counted as osteoclasts (scale bar, 200 μm). **H** OCPs were cultured with M-CSF (20 ng/ml) and RANKL (20 ng/ml) in the presence of B7–H3–Fc (5 μg/ml) or human IgG. The expression of the mature osteoclast markers, CTSK, TRAP, DC-STAMP, and ITGB3, was analyzed at day 8 using RT-qPCR. (I) Whole-cell lysates were immunoblotted with NFATc1 antibody. **C**, **D**, **H** The mRNA levels were normalized relative to GAPDH expression.

Next, we showed that B7–H3 knockdown was associated with a reduction of multinucleated TRAP-positive cells, decreased actin-ring formation, and suppressed osteoclast resorption of dentin discs (Fig. 2A). B7–H3 siRNA activity was confirmed in OCPs (Fig. 2B, D, E). Reduced osteoclast formation was not a consequence of changes in cell viability (Supplementary Fig. 1A). Furthermore, B7–H3 knockdown significantly suppressed RANKL-induced osteoclast-related gene expression such as cathepsin K (*CTSK*), tartrate-resistant acid phosphatase (*TRAP*), dendritic cell-specific transmembrane protein (*DC-STAMP*), and β3 integrin (*ITGB3*), as well as the protein expression of NFATc1 (Fig. 2C, E). Additionally, c-Fms was slightly reduced at day 0 without any changes in RANK expression (Fig. 2D, E). While B7–H3 knockdown slightly enhanced the phosphorylation of p38, it did not alter ERK, MAPK, and IκBα levels (Supplementary Fig. 2A). Next, we confirmed that the soluble recombinant B7–H3, protein (B7–H3–Fc) strongly suppressed the formation of RANKL-induced TRAP-positive cells in a dose-dependent manner (Fig. 2F, G) and significantly decreased the expression of *CTSK, TRAP, DC-STAMP*, and *ITGB3* as well as NFATc1 (Fig. 2H, I). The inhibitory effect of B7–H3, depending on the timing or duration of exposure, was assessed and B7–H3–Fc was found to decrease the number of osteoclasts, regardless of the phase of differentiation. (Supplementary Fig. 2B). Cell viability remained unaffected by B7–H3–Fc (Supplementary Fig. 1B). Taken together, the effects of B7–H3–Fc were consistent with that of B7–H3 siRNA, further supporting the regulatory function of B7–H3 in human osteoclastogenesis. On the other hand, micro-CT analysis of B7–H3 KO mice showed that there were no between-group differences in trabecular bone volume, thickness, number, spacing (Supplementary Fig. 3A, B). B7–H3 KO BMMs demonstrated reduced sizes of multinucleated TRAP-positive osteoclasts, actin-ring formation, and osteoclast-related genes compared with their wild-type (WT) counterparts. No between-group differences were seen in the number of osteoclasts (Supplementary Fig. 3C, D). These findings highlight the differences in the mechanism of B7–H3 in regulating osteoclast differentiation in mice and humans.

### B7–H3 deficiency activates IFN signaling

**Fig. 3:**
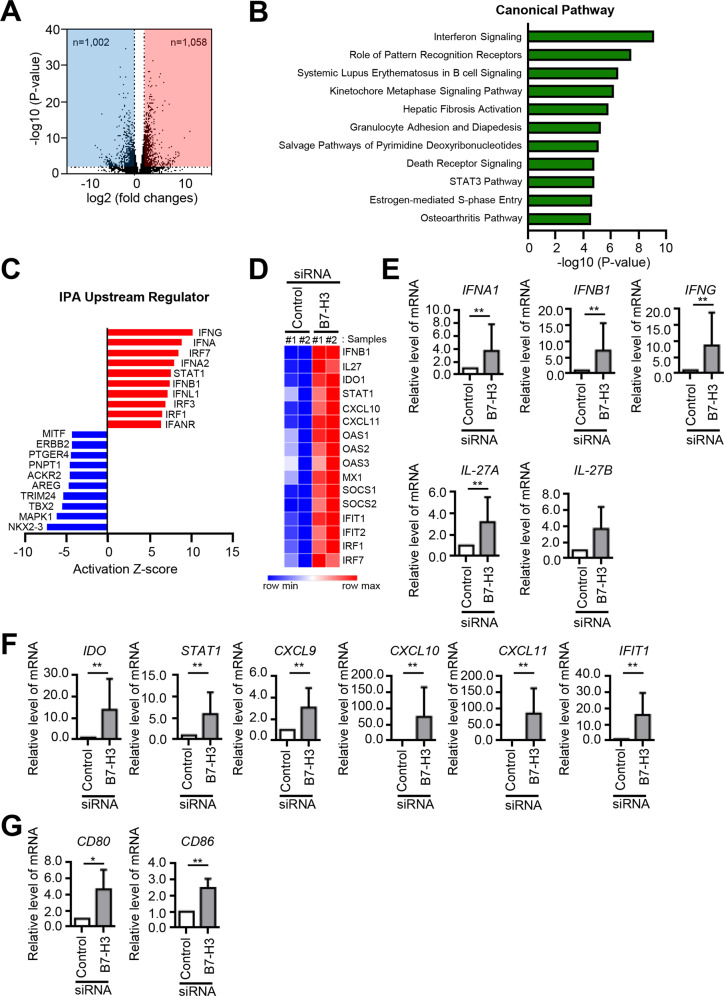
B7–H3 deficiency activates IFN signaling. **A** M-CSF (40 ng/ml) was added to monocyte culture for three days and then the cells were transiently transfected with B7–H3-specific siRNA or control siRNA (40 nM) for one day. Gene expression was analyzed using RNA sequencing (RNA-seq) and depicted using a volcano plot. The x axis indicates the log 2-fold changes in gene expression, such that data points plotted to the right indicate increased and left indicates decreased expression, respectively. **B** A simple bar graph depicting the 11 topmost enriched pathways. Higher values on the x axis indicate greater statistical significance. **C** A bar graph depicting the top 10 most upregulated (red) and downregulated (blue) potential upstream genes derived from Ingenuity Pathway Analysis (IPA). **D** Heatmap of RNA-seq CPM values for STAT1-activating and -dependent genes in B7–H3-deficient OCPs. RNA-seq data from 2 biological replicates were used. **E**, **F**, **G** Differentially expressed genes were analyzed using RT-qPCR. The mRNA levels were normalized relative to GAPDH expression.

Next, we searched for potential downstream mediators using transcriptomic analyses in B7–H3 siRNA-transfected OCPs and identified differentially expressed genes, many of which encode inflammatory cytokines (Fig. 3A, Supplementary Table S2). Among these genes, IPA revealed a pathway enrichment in IFN signaling (Fig. 3B) and top upstream-regulator predictions showed elevated activities of IFNs and STAT1 (Fig. 3C). Accordingly, B7–H3-deficient OCPs demonstrated upregulation of STAT1-activating and -dependent genes such as IFNs and IL-27 (Fig. 3D). These results were confirmed via RT-qPCR (Fig. 3E, F). Then, using B7–H3-expressing plasmid transfection, we showed that B7–H3 overexpression suppressed the expression of *IFNB, IFNG, IL-27A*, and *IL-27B* as well as IFN-response genes (Supplementary Fig. 4A–C). Also, we found that B7–H3 knockdown induced CD80/86 expression in the context of osteoclastogensis (Fig. 3G). Taken together, these results implicate IFNs and IL-27 as potential downstream mediators of B7–H3 in the regulation of osteoclast differentiation.

### The inhibition of osteoclastogenesis is restored by the addition of a neutralizing anti-IFN-αR2 antibody in B7–H3-deficient OCPs

**Fig. 4:**
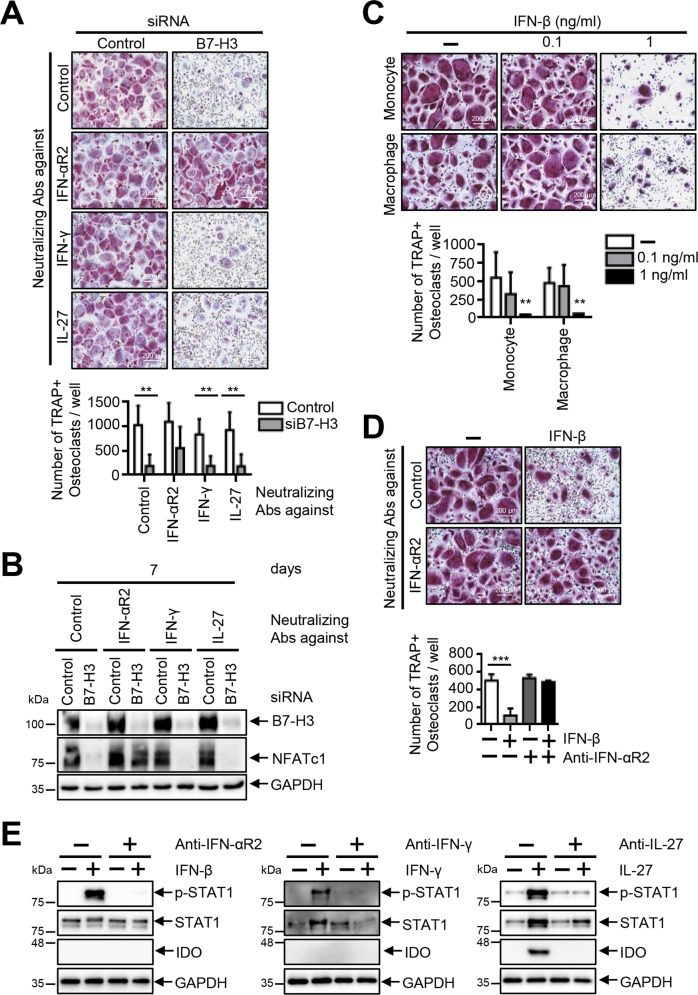
The inhibition of osteoclastogenesis is restored by the addition of a neutralizing anti-IFN-αR2 antibody in B7–H3-deficient OCPs. **A** M-CSF (40 ng/ml) was added in monocyte culture for three days. OCPs were transiently transfected with B7–H3-specific siRNA or control siRNA (20 nM) and then induced to differentiate using M-CSF (40 ng/ml) and RANKL (80 ng/ml) in the presence of neutralizing antibodies for IFN-αR2, IFN-γ, or IL-27 for seven days. **B** Whole-cell lysates were immunoblotted with B7–H3 and NFATc1 Abs. **C** Monocytes were cultured with M-CSF (20 ng/ml) in the presence of IFN-β (0.1 and 1 ng/ml) or distilled water for two days and then were further incubated with M-CSF (20 ng/ml) and RANKL (40 ng/ml) in the presence of recombinant IFN-β or distilled water for six days. OCPs, cultured with M-CSF (20 ng/ml) for two days, were induced to differentiate using M-CSF (20 ng/ml) and RANKL (40 ng/ml) in the presence of recombinant IFN-β (0.1 and 1 ng/ml) or distilled water for an additional six days. **D** OCPs were induced to differentiate using M-CSF (20 ng/ml) and RANKL (40 ng/ml) in the presence of anti-IFN-αR2 and recombinant IFN-β (1 ng/ml) for six days. (**A**, **C**, **D**) TRAP staining was performed and the number of TRAP-positive multinucleated cells per well were counted (scale bar, 200 μm). **E** Monocytes were incubated with or without neutralizing antibodies (2 μg/ml) against IFN-αR2, IFN-γ, or IL-27 for 1 h. Control antibodies were used at the equal concentration. At the end of the duration of culture, the cells were treated with recombinant IFN-β (10 ng/ml) or IFN-γ (10 ng/ml) for 10 m or recombinant IL-27 (50 ng/ml) for one day. The expression of phospho-STAT1 (PY701), STAT1, and IDO protein was evaluated via Western blot.

We then cultured B7–H3 siRNA-transfected OCPs in the presence of neutralizing antibodies to IFN-γ, IL-27, and IFN-αR2 to confirm the role of IFNs and IL-27 as downstream mediators. Whereas anti-IFN-γ and anti-IL-27 failed to affect osteoclast differentiation, including the expression of NFATc1, anti-IFN-αR2 successfully restored osteoclastogenesis inhibition in B7–H3 siRNA-transfected OCPs (Fig. 4A, B). We focused our investigations on the effect of IFN-β since previous studies showed that IFN-β, and not IFN-α, inhibits RANKL-induced osteoclastogenesis. IFN-β dose-dependently suppressed osteoclast differentiation, while anti-IFN-αR2 successfully reversed this IFN-β-mediated suppression of osteoclastogenesis (Fig. 4C–E). Thus, these findings indicate that type-I IFNs rather than type-II IFNs or IL-27 are the most likely candidate downstream mediators of B7–H3 in the regulation of osteoclastogenesis.

### The inhibition of osteoclastogenesis in B7-H3-deficient OCPs is not mediated by previously identified mechanisms modulated by IFN-β

**Fig. 5:**
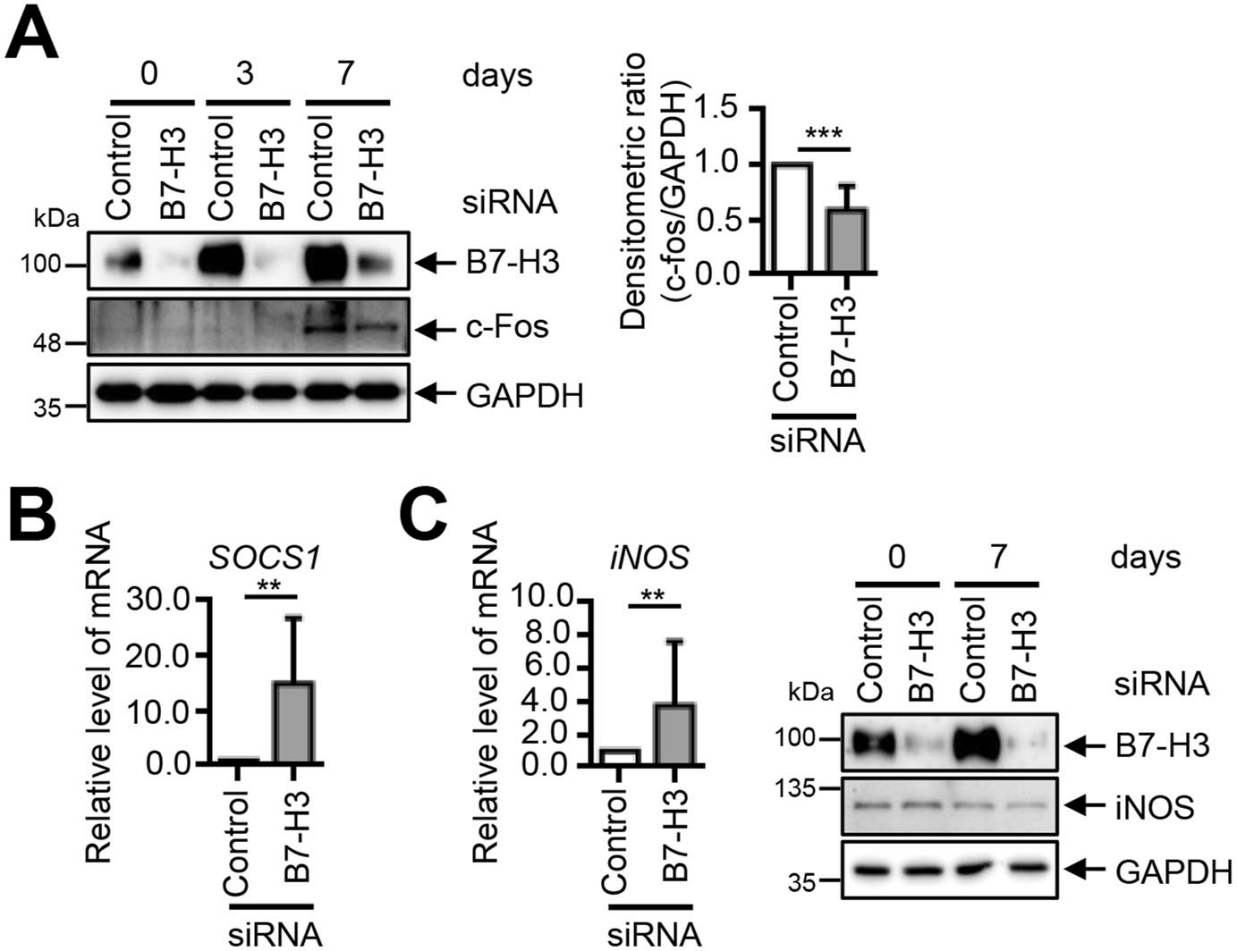
B7–H3 deficiency-mediated type-I IFN-dependent osteoclastogenesis inhibition is independent of c-Fos, iNOS, and SOCS1. **A**, **B** M-CSF (40 ng/ml) was added to monocyte culture for three days. OCPs that were transiently transfected with B7–H3-specific siRNA or control siRNA (40 nM) were induced to differentiate using M-CSF (40 ng/ml) and RANKL (80 ng/ml) for seven days. The expression of B7–H3, c-Fos and iNOS protein during osteoclast differentiation was detected via Western blot. The bar graphs show the densitometry analyses of the gels depicted relative to the internal control at day 7 of differentiation. **B**, **C** OCPs were transiently transfected with B7–H3-specific siRNA or control siRNA (40 nM) for one day. The expression of SOCS1 and iNOS mRNA was analyzed using RT-qPCR. The mRNA levels were normalized relative to GAPDH expression.

Next, we investigated the downstream mediators of type-I IFNs. We found that B7–H3 knockdown inhibited c-Fos expression. However, the c-Fos protein was only weakly inhibited and therefore unlikely to mediate B7–H3 siRNA-mediated osteoclastogenesis inhibition (Fig. 5A). We showed that B7–H3 siRNA upregulated SOCS1 expression, which is inconsistent with the previous studies showing that IFN-β-mediated osteoclastogenesis inhibition occurs through SOCS1 downregulation via miR-155 induction (Fig. 5B). We found that although the iNOS mRNA levels increased, its protein levels did not increase following B7–H3 knockdown (Fig. 5C). Taken together, c-Fos, SOCS1, and iNOS are unlikely candidate downstream mediators of type-I IFN-mediated osteoclastogenesis inhibition in B7–H3-deficient OCPs.

### IDO induction mediates osteoclastogenesis inhibition in B7–H3-deficient OCPs

**Fig. 6:**
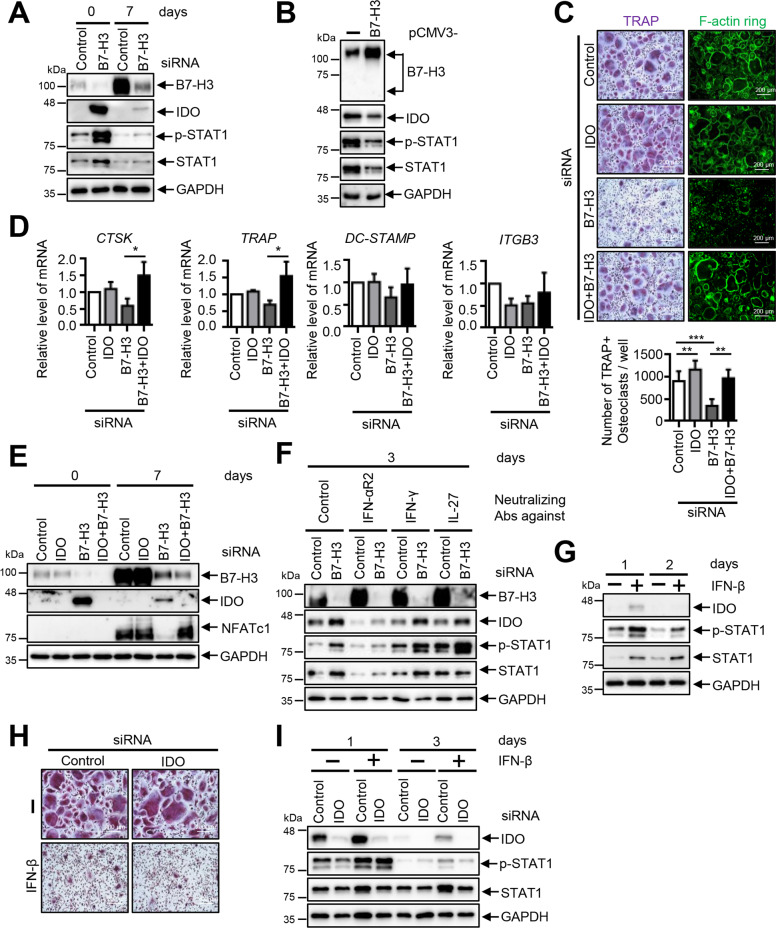
The inhibition of osteoclastogenesis is mediated by increased IDO expression in B7–H3-deficient OCPs. **A** M-CSF (40 ng/ml) was added to monocyte culture for three days. OCPs that were transiently transfected with B7–H3-specific siRNA or control siRNA (40 nM) were induced to differentiate using M-CSF (40 ng/ml) and RANKL (80 ng/ml) for seven days. **B** The M-CSF (40 ng/ml) was added in monocyte culture for three days, then the cells were transfected with human pCMV3–B7–H3 ORF-expression plasmid or pCMV3-negative control vector (2 μg) for one day. The expression of B7–H3, IDO, phospho-STAT1 (PY701), and STAT1 protein during osteoclast differentiation was analyzed by Western blot. **C** OCPs that were transiently transfected with B7–H3-specific siRNA (20 nM), IDO-specific siRNA (60 nM), control siRNA (80 nM), or both B7–H3- and IDO-specific siRNA were induced to differentiate using M-CSF (40 ng/ml) and RANKL (80 ng/ml) for seven days. The cells were stained for TRAP expression and TRAP-positive multinucleated cells were counted as osteoclasts. Actin rings in osteoclasts stained with FITC-phalloidin (scale bar, 200 μm). **D** The expression of mature osteoclast markers, CTSK, TRAP, DC-STAMP, and ITGB3, was analyzed using RT-qPCR. The mRNA levels were normalized relative to GAPDH expression. **E** The expression of B7–H3, IDO, and NFATc1 protein was analyzed via Western blot. **F** OCPs that were transiently transfected with B7–H3-specific siRNA or control siRNA (20 nM) were induced to differentiate using M-CSF (40 ng/ml) and RANKL (80 ng/ml) in the presence of anti-IFN-αR2, -IFN-γ, or -IL-27 for three days. The expression of B7–H3, IDO, phospho-STAT1 (PY701), and STAT1 protein was analyzed via Western blot. **G** Monocytes were treated with recombinant IFN-β (1 ng/ml) or distilled water for one or two days. Whole-cell lysates were immunoblotted with IDO, phospho-STAT1 (PY701), and STAT1 antibodies. **H** OCPs that were transiently transfected with IDO-specific siRNA or control siRNA (60 nM) were induced to differentiate using M-CSF (40 ng/ml) and RANKL (80 ng/ml) in the presence of recombinant IFN-β (1 ng/ml) or distilled water for seven days. The cells were stained for TRAP expression (scale bar, 200 μm). **I** At days 1 and 3, whole-cell lysates were immunoblotted with IDO, phospho-STAT1 (PY701), and STAT1 antibodies.

Next, we found that IDO protein expression and STAT1 phosphorylation were dramatically induced in B7–H3-deficient and B7–H3 Fc-treated OCPs and that B7–H3 deletion led to a sustained constitutive expression of IDO throughout osteoclastogenesis (Fig. 6A, Supplementary Fig. 5A, B). Conversely, B7–H3 overexpression suppressed IDO protein expression and STAT1 phosphorylation in OCPs (Fig. 6B). Moreover, IDO knockdown reversed osteoclastogenesis inhibition and the suppression of osteoclastogenesis-related genes as well as NFATc1 protein expression (Fig. 6C–E). Furthermore, anti-IFN-αR2 successfully reversed the IDO induction caused by B7–H3 siRNA and these results were confirmed by demonstrating that type-I IFNs increased IDO expression and reduced multinucleated TRAP-positive cell formation (Fig. 6F, G). However, IFN-β-mediated osteoclast inhibition was not reversed by IDO knockdown (Fig. 6H, I). Therefore, while osteoclastogenesis inhibition in the context of B7–H3 knockdown appears to be mediated at least in part by type-I IFN-mediated IDO induction, our findings raise the possibility that additional unidentified mechanisms may be present.

### IDO-mediated inhibition of osteoclastogenesis in B7–H3-deficient OCPs is independent of tryptophan metabolism

**Fig. 7:**
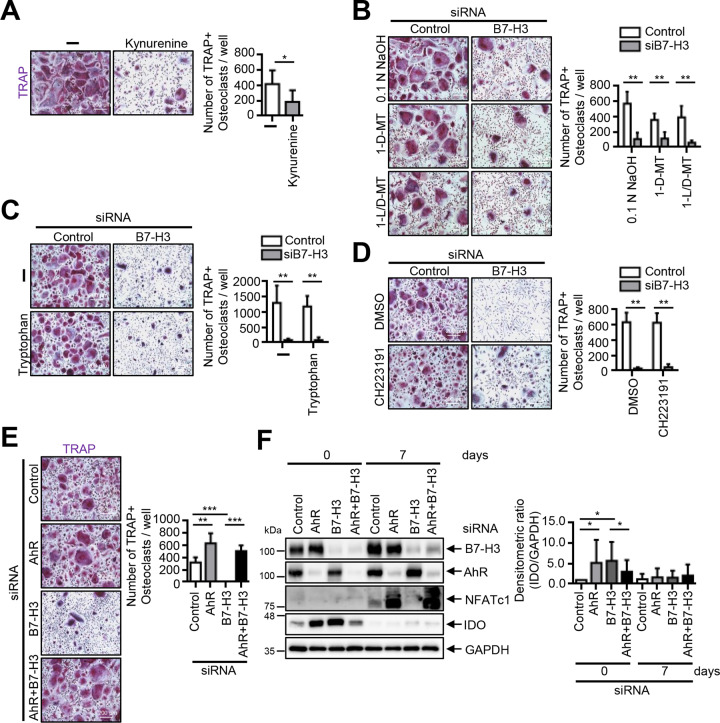
The inhibition of osteoclastogenesis is independent of the IDO/tryptophan pathway in B7–H3-deficient OCPs. **A** Monocytes were cultured with M-CSF (20 ng/ml) for two days, then M-CSF (20 ng/ml) and RANKL (40 ng/ml) were added for an additional six days in the presence of kynurenine (100 μM) or DMSO. **B** M-CSF (40 ng/ml) was added to monocyte culture for three days. OCPs that were transiently transfected with B7–H3-specific siRNA or control siRNA (20 nM) were induced to differentiate using M-CSF (40 ng/ml) and RANKL (80 ng/ml) for seven days with coadministration of 1-D-MT (200 μM), 1-L/D-MT (200 μM), or 0.1 N NaOH (control). **C** The experiment in (**B**) was conducted, except with coadministration of tryptophan (1 mM) or distilled water (control). **D** The experiment in (B) was conducted, except with coadministration of CH223191 (5 μM) or DMSO (control). **E** The OCPs that were transiently transfected with B7–H3-specific siRNA (20 nM), AhR-specific siRNA (40 nM), control siRNA (60 nM), or both B7–H3- and AhR-specific siRNA were induced to differentiate using M-CSF (40 ng/ml) and RANKL (80 ng/ml) for seven days. **F** Whole-cell lysates were immunoblotted with B7–H3, AhR, NFATc1, and IDO Abs. The bar graphs show the densitometry analyses of the gels depicted relative to the internal control. (**A**, **B**, **C**, **D**, **E**) TRAP staining was performed and the number of TRAP-positive multinucleated cells per well were counted as osteoclasts (scale bar, 200 μm).

We found that multinucleated TRAP-positive osteoclasts were strongly suppressed by kynurenine (Fig. 7A). We then cultured B7–H3 siRNA-transfected OCPs in the presence of 1-MT (IDO inhibitor), exogenous tryptophan, and CH223191 (AhR inhibitor). 1-MT and exogenous tryptophan failed to reverse B7–H3 siRNA-mediated osteoclastogenesis inhibition whereas CH223191 altered multinucleated osteoclast morphology without causing changes in the number of cells (Fig. 7B–D). Furthermore, AhR siRNA restored B7–H3 deficiency-mediated suppression of osteoclastogenesis (Fig. 7E, F). In one out of five of the repeat experiments, AhR siRNA failed to restore osteoclastogenesis inhibition, which was concluded to be due to the unsuccessful suppression of IDO by AhR siRNA (Supplementary Fig. 6A, B). Nonetheless, AhR siRNA alone enhanced IDO expression in OCPs. Taken together, these results suggest that the AhR-associated signaling pathway may be involved in B7–H3-mediated osteoclastogenesis.

### B7–H3 deficiency does not affect osteoclast differentiation in RA synovial-fluid macrophages

**Fig. 8:**
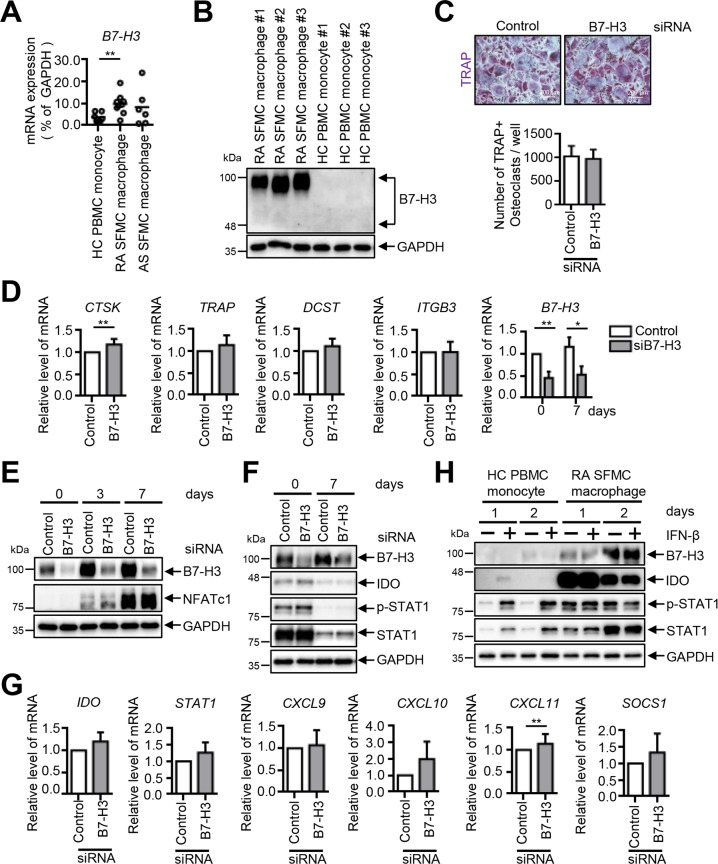
B7–H3 deficiency does not affect osteoclast differentiation in RA synovial-fluid macrophages. **A** The expression of B7–H3 mRNA was analyzed using RT-qPCR on human HC PBMCs and RA and AS synovial fluid macrophages. Monocytes were purified from PBMCs (*n* = 7), and macrophages were derived from RA synovial-fluid mononuclear cells (*n* = 9) and AS synovial-fluid mononuclear cells (*n* = 6). The results are shown as mean ± S.E.M. **B** The expression of B7–H3 protein between HC PBMCs and RA synovial-fluid macrophages (*n* = 3) was assessed by Western blot. **C** M-CSF (40 ng/ml) was added to RA synovial-fluid macrophages for three days. OCPs that were transiently transfected with B7–H3-specific siRNA or control siRNA (40 nM) were induced to differentiate using M-CSF (40 ng/ml) and RANKL (80 ng/ml) for seven days. TRAP staining was performed and the number of TRAP-positive multinucleated cells per well were counted as osteoclasts (scale bar, 200 μm). **D** The expression of the mature osteoclast markers, CTSK, TRAP, DC-STAMP, and ITGB3, and B7–H3, was analyzed by RT-qPCR. **E**, **F** The expression of B7–H3, NFATc1, IDO, phospho-STAT1 (PY701), and STAT1 protein was evaluated using Western blot. **G** The expression of IFN-inducible genes was analyzed using RT-qPCR at day 0. **H** HC PBMCs and RA synovial-fluid macrophages were treated with recombinant IFN-β (1 ng/ml) for one or two days. Whole-cell lysates were immunoblotted with B7–H3, IDO, phospho-STAT1 (PY701), and STAT1 antibodies. (**A**, **D**, **G**) The mRNA levels were normalized relative to GAPDH expression.

We then examined the B7–H3-expression levels in RA synovial-fluid macrophages compared with HC PBMCs and ankylosing-spondylitis (AS) synovial-fluid macrophages. B7–H3 mRNA levels were higher in RA synovial macrophages compared with HC PBMCs and no significant differences in B7–H3 mRNA levels were found between AS synovial-fluid macrophages and HC PBMCs (Fig. 8A). Consistently, B7–H3 protein levels were also higher in RA synovial-fluid macrophages (Fig. 8B). In contrast to HC PBMCs, B7–H3 knockdown did not affect RA synovial macrophage osteoclast differentiation and the expression of osteoclast-related genes as well as NFATc1 protein (Fig. 8C–E). Furthermore, IDO expression and STAT1 phosphorylation, as well as IFN-response gene expression, remained largely unchanged by B7–H3 knockdown and by IFN-β stimulation in RA synovial-fluid macrophages (Fig. 8F–H). Taken together, these results indicate that B7–H3 has differential effects on osteoclast differentiation in RA synovial-fluid macrophages compared with PBMCs.

## Discussion

In summary, we found that inhibition of B7–H3 using B7–H3 siRNA and soluble B7–H3–Fc treatment led to osteoclastogenesis inhibition and to increased type-I IFN signaling, STAT1 activation, and IDO induction. In addition, type-I IFN and IDO suppression reversed osteoclastogenesis inhibition, while type-I IFN blockade suppressed B7–H3 siRNA-mediated IDO induction. Taken together, we add to the emerging paradigm in which immune checkpoint molecules moonlight as regulatory molecules in bone remodeling whereby costimulatory molecules (B7.1/7.2, B7–H2) inhibit, while co-inhibitory molecules (B7–H3) stimulate osteoclastogenesis [12, 13].

The role of B7–H3 in innate immune cells, which share the same monocytic lineage as osteoclasts, remains controversial. In mouse-derived BMMs, soluble B7–H3 (muB7–H3) stimulates NF-kB activation and proinflammatory cytokine release, indicating that B7–H3 exerts costimulatory effects on murine innate immune cells [15]. In a previous study that showed the costimulatory effect of B7–H3 in murine microglial cells, muB7–H3 was used as an agonist, whereas a specific blocking antibody against B7–H3 was used as an antagonist [16]. Conversely, B7–H3–Fc and B7–H3 siRNA both showed anti-osteoclastogenic effects in our study, which is consistent with a previous study conducted in the context of osteoblast differentiation [14]. The discrepancy of these results may be explained in part by differences in experimental model and/or design.

Type-I IFNs regulate osteoclastogenesis via several mechanisms, including the inhibition of c-Fos and iNOS/NO signaling and indirect inhibition of SOC1 and MITF through miR-155 induction [17–20]. Our results suggest that mechanisms other than iNOS, SOCS1, and c-Fos are responsible for mediating the effects of type-I IFNs on osteoclastogenesis. Furthermore, our study implicates IDO, which is an IFN-inducible gene in the regulation of osteoclastogenesis, suggesting that B7–H3 inhibits the type-I IFN–IDO pathway to stimulate osteoclast differentiation. IDO acts either as a tryptophan-degrading enzyme or an intracellular signal transducer activated by transforming growth factor-β (TGF-β) [21–23]. Our results suggest that AhR signaling is crucial for B7–H3-mediated osteoclastogenesis inhibition. Notably, in our experiments, AhR siRNA alone enhanced IDO expression in OCPs. Since AhR is known to suppress STAT1 activity, AhR siRNA alone may have led to the increase in IDO, which is a STAT1-dependent gene [24, 25].

Furthermore, the reason for the discrepancy between the AhR siRNA experiment and the 1-MT and CH223191 experiments is that kynurenine is only one of many endogenous AhR agonizts. Consequently, 1-MT-mediated kynurenine inhibition may have been insufficient to inhibit other endogenous AhR agonizts. Also, CH223191, while known as an AhR inhibitor, is a ligand-selective antagonist that preferentially inhibits agonizts of the halogenated aromatic hydrocarbons (HAH). The action of endogenous AhR ligands may not be inhibited by CH223191 as they are more likely to bind AhR-like non-HAH agonizts [26]. Therefore, whether CH223191 blocks the endogenous ligand activation of AhR needs to be confirmed.

No significant bone-phenotypic changes were seen in B7–H3 KO mice in our study. The most likely hypothesis for this finding is that B7–H3 KO has opposing effects on osteoclasts and osteoblasts, such that there is no significant alteration in their relative balance [14]. To directly test the causal relationship between B7–H3 expression and osteoclast differentiation in vivo, a genetic approach is required in which B7–H3 is specifically deleted in myeloid-lineage OCPs. For example, using the experimental model in which B7–H3-floxed mice are crossed with lysozyme M-Cre mice may be appropriate. Nonetheless, in our in vitro study, BMMs derived from B7–H3 KO mice showed lower osteoclastogenic potential and osteoclast-related gene expression, which warrants this further experiment to observe bone-phenotypic changes.

We show that unlike in PBMCs, B7–H3 knockdown does not result in osteoclastogenesis inhibition in RA synovial-fluid macrophages. This finding raises the possibility that the B7–H3–IFN–IDO axis in healthy peripheral-blood OCPs is dysfunctional in the RA synovium. We further showed that the concentration of IFN-β capable of inducing IDO in healthy peripheral blood OCPs does not induce IDO nor STAT1 activation in RA synovial macrophages. This suggests that RA synovial macrophages have an attenuated response to exogenous IFN-β, which is consistent with many previous studies that have established the attenuated responses of RA synovial macrophages to inflammatory cytokines such as IL-6, -10, and -27 [27–30]. Therefore, further research focused on RA synovial macrophages is necessary.

Various mechanisms mediate the immune-regulatory effects of IDO [31]. In dendritic cells, B7.1/7.2 activates intracellular signaling to induce *IDO* transcription and IDO-dependent immune tolerance [32]. The function of B7 molecules is not limited to immune regulation but also includes osteoclastogenesis regulation. Bozec et al. demonstrated that RA patients treated with abatacept (CTLA-4-Fc), which is approved for clinical use in RA, remarkably reduced osteoclast precursors and downregulated osteoclast-differentiation markers such as c-Fos, NFATc1, c-Fms, and RANK [12]. Many clinical trials also show the bone-protective effect of abatacept in RA, suggesting that in inflammatory bone-resorptive disorders, B7 molecules represent a novel therapeutic avenue for the development of anti-osteoclastogenic therapy.

Both B7.1/7.2- and CD200R1-mediated IDO induction depend on type-I IFNs [33–35]. We showed that B7–H3 inhibition-dependent IDO induction is also mediated by type-I IFNs. B7–H3 inhibition increases the production of anti-DNA autoantibodies and exacerbates glomerulonephritis in lupus mouse models and type-I IFNs are central to lupus pathogenesis [36, 37]. We further observed that deletion of B7–H3 induces B7.1/7.2, suggesting that B7–H3 inhibits B7.1/7.2-induced IDO expression in osteoclast differentiation. Taken together with our findings showing that B7–H3 inhibits type-I IFNs in human macrophages, the association between B7–H3 inhibition and increased autoantibody production and worsening glomerulonephritis may be mediated by type-I IFN activation. Therefore, B7–H3 may inhibit type-I IFNs and disease activity and has the therapeutic potential in patients with lupus.

A previously reported study established that B7–H3 is required to induce bone mineralization and osteoblast differentiation [14]. Adding to this finding, we found that while trabecular bone mass as assessed by micro-CT analysis is unaffected in B7–H3 KO mice, mouse BMMs derived from these mice had reduced osteoclast size. Thus, our investigation suggests that B7–H3 may be involved in regulating the balance between osteoclast and osteoblast formation.

Our study has several limitations. First, exogenous IFN-β and B7–H3 inhibition both led to IDO induction, whereas IDO knockdown led to reversal of osteoclastogenesis inhibition only in the setting of B7–H3 inhibition and not exogenous IFN-β administration. Second, the mechanism of IDO-mediated osteoclastogenesis inhibition in the setting of B7–H3 inhibition or deficiency remains unknown. Third, the reason that B7–H3 deficiency does not lead to osteoclastogenesis inhibition in RA synovial macrophages remains unclear. Fourth, the mechanism of osteoclastogenesis inhibition in B7–H3 KO mice and the impact of B7–H3 KO in specific disease-model mice remains unclear. Last, the direct role of B7–H3 as a direct positive regulator of osteoclastogenesis was not evaluated mainly because of the lack of available B7–H3 agonizts since sB7–H3–Fc acted as an antagonist in our experiments. Although our gain-of-function study revealed that B7–H3 overexpression led to STAT1 inhibition and STAT-1 dependent genes, the transient nature of B7–H3 overexpression precluded further assessment of the impact on osteoclastogenesis. Future research is warranted in examining the above questions to further elucidate the mechanism of B7–H3 inhibition in the negative regulation of osteoclastogenesis.

In conclusion, B7–H3 deficiency leads to inhibition of osteoclast differentiation, and the type-I IFN–IDO pathway is an important downstream-regulatory mechanism in B7–H3-mediated regulation of osteoclastogenesis. Our study highlights B7–H3 as a strong potential therapeutic target in human diseases in which B7–H3 type-I IFN–IDO-mediated regulation of immune responses and osteoclast activation play important pathogenic roles.

## Supplementary information

Supplementary figures and legends

Table S1

Table S2

## Data Availability

The authors confirm that the data supporting the findings of this study are available within the article and its supplementary materials.
