# Supplementary material for: B7–H3 regulates osteoclast differentiation via type I interferon-dependent IDO induction
Source: Cell Death Dis. 2021 Oct 20;12(11):971. doi: 10.1038/s41419-021-04275-6 (PMC8528854; doi:10.1038/s41419-021-04275-6)
Supplement: Supplementary file 1 — Supplementary figures and legends [file 41419_2021_4275_MOESM1_ESM.pdf]

Oh_Supplementary Fig.S1

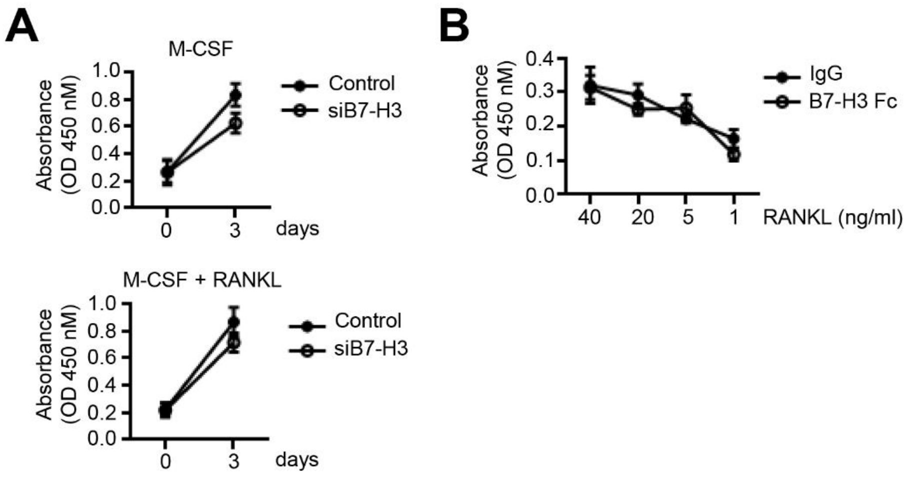

Supplementary S1. B7-H3 does not affect cell viability in human HC PBMCs. (A) Monocytes were cultured in M-CSF (40 ng/ml) for 3 days. B7-H3-specific siRNA or control siRNA (40 nM) transfected OCPs were incubated in the presence or absence of RANKL (80 ng/ml). At day 0 and 3 of incubation, cell viability was measured using the Ez-cytox viability kit reagent. (B) OCPs were cultured with M-CSF (20 ng/ml) and RANKL (40, 20, 5, 1 ng/ml) in the presence of B7-H3 Fc (5 μg/ml) or human IgG for 8 days. Cell viability was measured using the Ez-cytox viability kit.

Oh_Supplementary Fig.S2

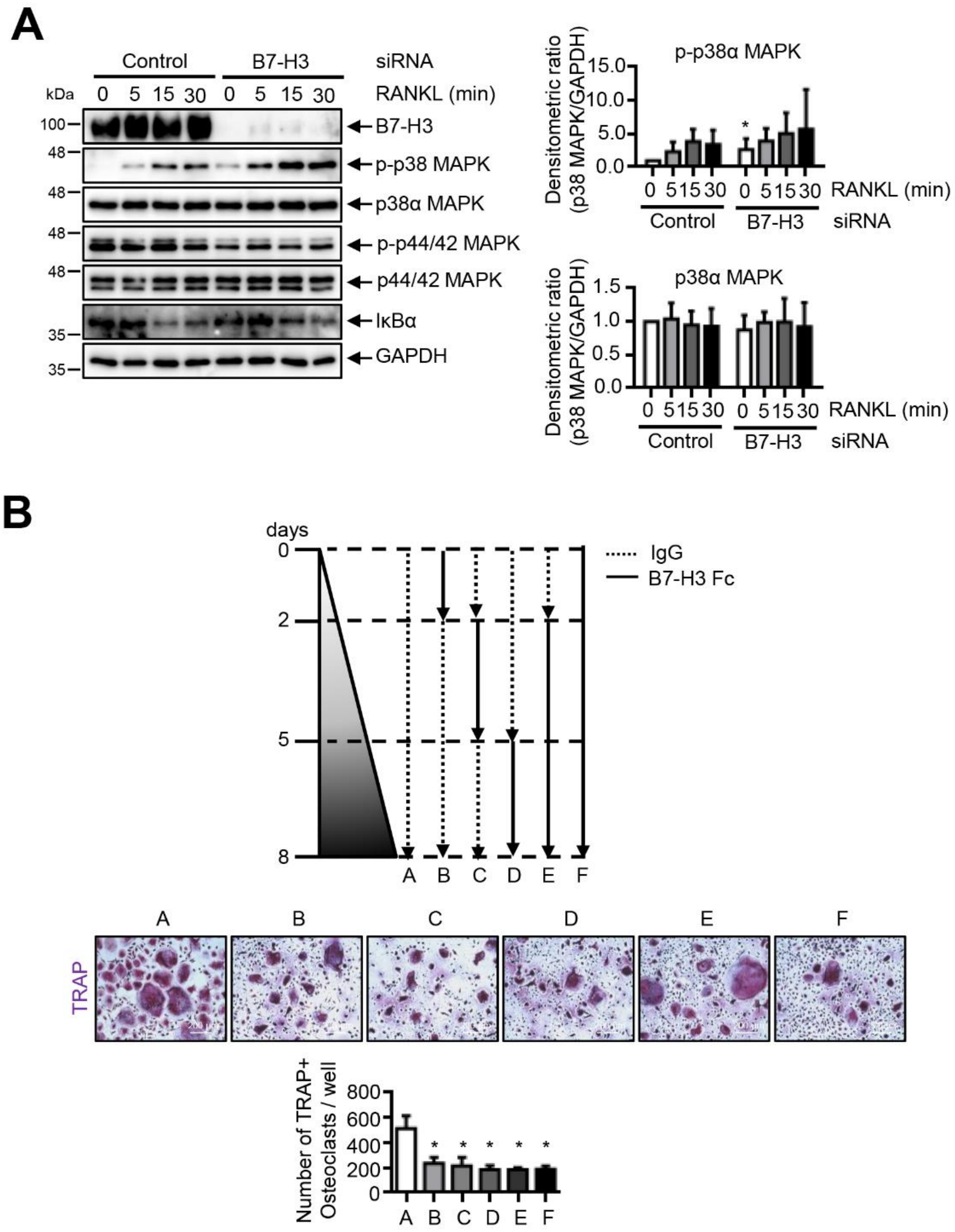

Supplementary S2. Inhibition of B7-H3 suppresses RANKL- induced human osteocalstogensis. (A) OCPs were transiently transfected with B7-H3-specific siRNA or control siRNA (40 nM), then removed from serum for 6 hours. The cells were stimulated with RANKL (40 ng/ml) for the indicated times. Western blot was performed to detect expression of the

indicated proteins. The bar graphs show the densitometry analyses of the gels quantified relative to the internal control. The mRNA levels were normalized relative to GAPDH expression. (B) In the absence or presence of B7-H3-Fc (5 μg/ml) for indicated time, monocytes were cultured with M-CSF (20 ng/ml) for 2 days, after which, RANKL (20 ng/ml) was added and the cells cultured for an additional 6 days. TRAP staining was performed and the number of TRAP-positive multinucleated cells per well were counted as osteoclasts (scale bar, 200 μm).

Oh_Supplementary Fig.S3

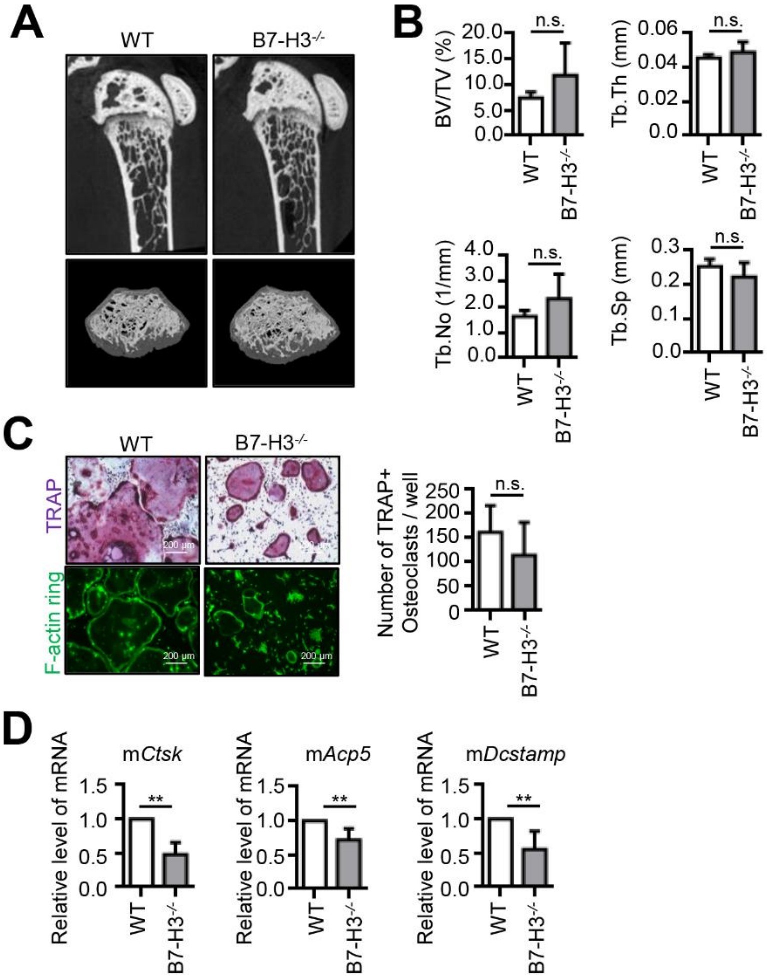

Supplementary S3. B7-H3 affects osteoclast differentiation in mouse BMMs. (A, B) Representative microCT images of the femur (upper panel) and trabecular (Tb) bone (lower panel) from WT or B7-H3$^{-/-}$ male mice (n = 3 per group). (B) Tb bone volume per tissue volume (BV/TV), Tb thickness (Th), number (No) and spacing (Sp) were determined by microCT analysis. (C) Mouse BMMs derived from WT or B7-H3$^{-/-}$ male mice (n = 5 per group) were cultured in the presence of M-CSF (50 ng/ml) and RANKL (100 ng/ml). After 4 days, TRAP staining was performed, and the number of TRAP- positive multinucleated osteoclasts per well was counted. Actin ring in osteoclasts stained with FITC-phalloidin (Scale bar, 200 μm). (D) The expression of

mature osteoclast marker; mCtsk, mAcp5 and mDcstamp was analyzed by RT-qPCR at 4 days. The mRNA levels were normalized relative to the expression of GAPDH. n.s., not significant.

Oh_Supplementary Fig.S4

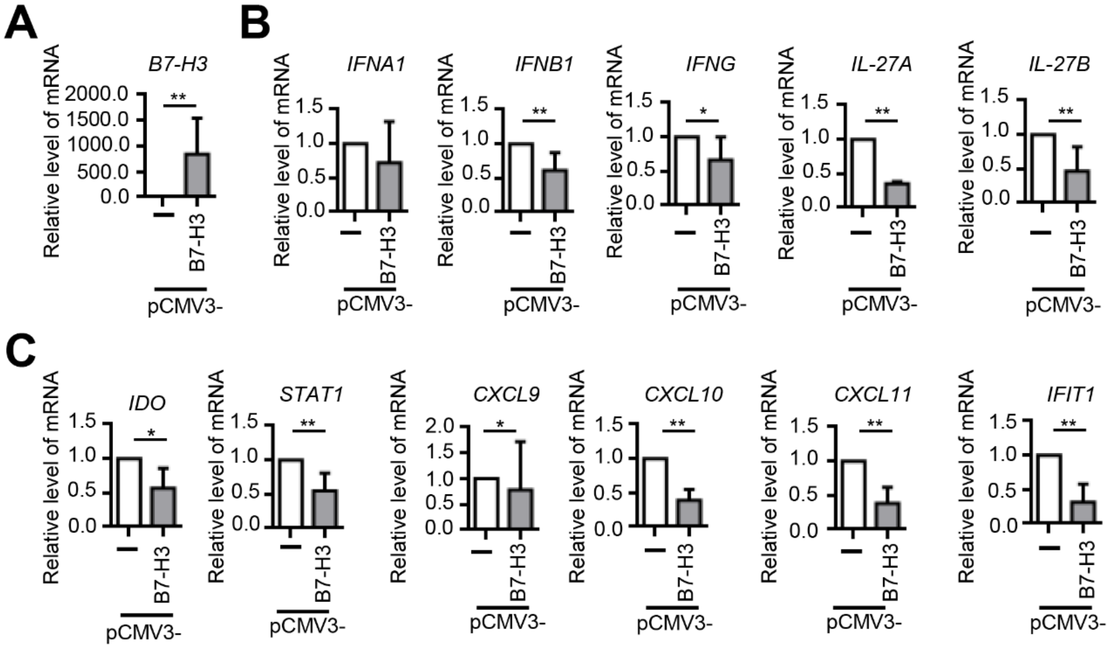

Supplementary S4. B7-H3 overexpression leads to decreased IFN signaling. (A, B) M-CSF (40 ng/ml) was added on monocytes for 3 days and then the cells were transfected with human pCMV3-B7-H3 ORF expression plasmid or pCMV3-negative control vector (2 μg) for 1 day. RT-qPCR was used to evaluate the expression of B7-H3, IFNs, and IL-27. (C) Same as (A, B) except the expression of IFN-inducible gene was analyzed. The mRNA levels were normalized relative to GAPDH expression.

Oh_Supplementary Fig.S5

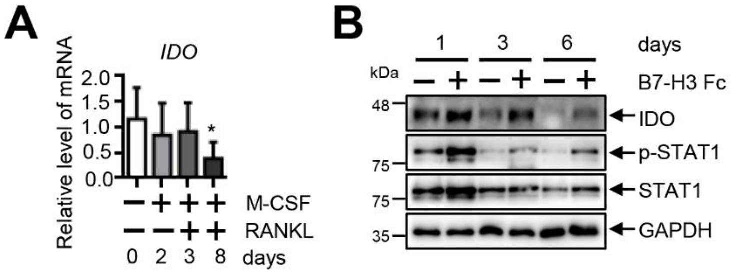

Supplementary S5. B7-H3 Fc induces IDO protein expression. (A) As described for Fig. 1A, the expression of IDO mRNA during human osteoclast differentiation was detected by RT-qPCR. (B) OCPs, cultured with M-CSF (20 ng/ml) for 2 days, were incubated with M-CSF (20 ng/ml) and RANKL (40 or 20 ng/ml) in the presence of B7-H3-Fc (5 μg/ml) or human IgG for 6 days. The expression of IDO, phospho-STAT1 (PY701), and STAT1 protein was analyzed by Western blot.

Oh_Supplementary Fig.S6

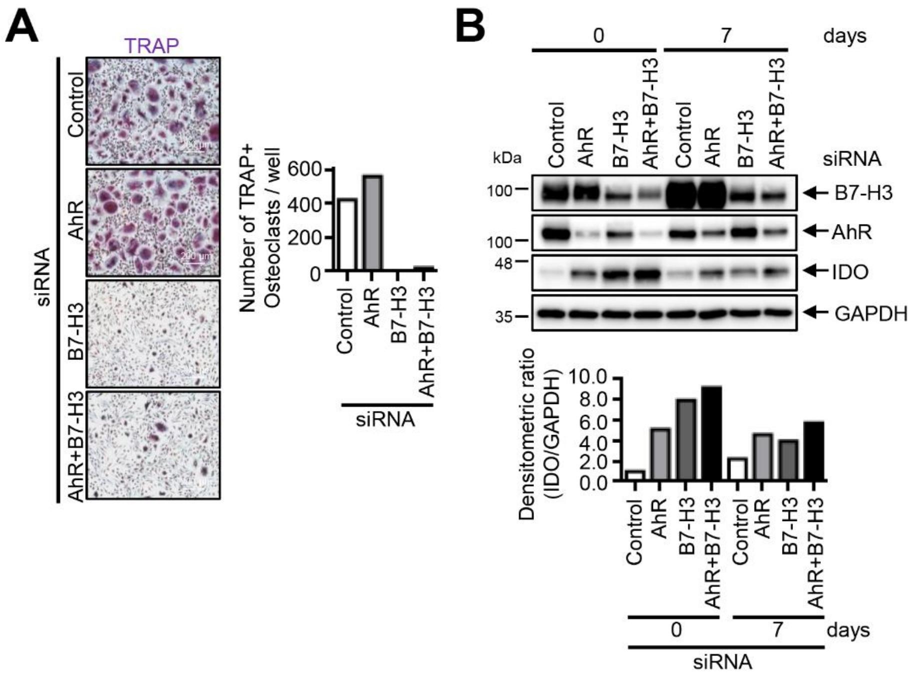

Supplementary S6. AhR siRNA is unable to inhibit osteoclastogenesis due to failure to inhibit IDO. (A) In 1 out of 5 of the repeat experiments, AhR failed to reverse the inhibition of osteoclastogenesis in B7-H3 deficient osteoclasts. The cells were stained for TRAP expression and TRAP-positive multinucleated cells were counted as osteoclasts (Scale bar, 200 μm). (B) Whole-cell lysates were immunoblotted with B7-H3, AhR and IDO Abs. The bar graphs show the densitometry analyses of the gels referred to the relative internal control.
