# Supplementary material for: B7–H3 regulates osteoclast differentiation via type I interferon-dependent IDO induction
Source: Cell Death Dis. 2021 Oct 20;12(11):971. doi: 10.1038/s41419-021-04275-6 (PMC8528854; doi:10.1038/s41419-021-04275-6)
Supplement: Supplementary file 2 — Table S1 [file 41419_2021_4275_MOESM2_ESM.pdf]

Supplementary Table S1. List of primer sequences used for RT-qPCR analysis.

**RT-qPCR primer sequences**		
**Gene**	**Forward Primer (5'→3')**	**Reverse Primer (5'→3')**
B7-H3	GTCCCTGAGTCCCAGAGT	GGTCCTCAGCTCCTGCATT
CTSK	CTCTTCCATTTCTTCCACGAT	ACACCAACTCCCTTCCAAAG
TRAP	TGGCTTTGCCTATGTGGA	CCTGGTCTTAAAGAGGGACTT
DC-STAMP	AAAGCTTGCCAGGGTTTGAG	GGTTTTGGGATACAGTTGGGTTC
ITGB3	GGAAGAACGCGCCAGAGCAAAATG	CCCCAAATCCCTCCCCACAAATAC
CSF1R	GGTACTGCTGTAATGAGCCAA	AGTTTGTGCTTCCTGCTTGGT
RANK	CCATCATCTTTGGCGTTTG	AGCTGTGAGTGCTTTCCCT
IFNA1	CCAGTTCCAGAAGGCTCCAG	CCTCTCCTCCTGCATCACAC
IFNB1	CTAGCACTGGCTGGAATGAGA	TCCTTGGCCTTCAGGTAATGC
IFNG	ACCAGAGCATCCAAAAGAGTGT	TTAGCTGCTGGCGACAGTTC
IL-27A	CAGACGGCAGGCGACCTT	GAGATGCAGGCTGACTGTGAA
IL-27B	GTTCTCCATGGCTCCCTACG	AAGATCTCTGGGAAGGGCCA
STAT1	TGGGTTTGACAAGGTTCTT	TATGCAGTGCACGGAAAG
CXCL9	AGAAAGGGTCGCTGTTCCTG	TCACATCTGCTGAATCTGGGTT
CXCL10	TGTACGCTGTACCTGCATCA	TTGATGGCCTTCGATTCTGGA
CXCL11	GAAGGATGAAAGGTGGGTGA	AAGCACTTTGTAAACTCCGATG
IFIT1	GTGCTTGAAGTGGACCCTGA	AGCTTCAGGGCAAGGAGAAC
IDO	TTAGAGTCAAATCCCTCAGTCC	TTTGCAGATGGTAGCTCCTC
iNOS	AATGTGGAGAAAGCCCCCTG	TGCATCCAGCTTGACCAGAG
SOCS1	CTTCGACTGCCTTTTCGAGC	GGAGTACCGGGTTAAGAGGG
CD80	TGGTGCTGGCTGGTCTTTC	CTGTGCCACTTCTTTCACTTCC
CD86	ACATTCTCTTTGTGATGGCCTTC	TGCAGTCTCATTGAAATAAGCTTGA
GAPDH	CAAGATCATCAGCAATGCC	CTGTGGTCATGAGTCCTTCC
**m*B7-H3***	GACACGGATGCCACCCTACGCTG	CTGTGATGGTGACTGAGCCGTGAG
**m*Ctsk***	TGGAGGCGGCTATATGACCA	CCTTTGCCGTGGCGTTATAC
**m*Acp5***	ACGGCTACTTGCGGTTTC	TCCTTGGGAGGCTGGTC
**m*Dcstamp***	TCCTCCATGAACAAACAGTT	AGACGTGGTTTAGGAATGCA
