# Supplementary material for: B7–H3 regulates osteoclast differentiation via type I interferon-dependent IDO induction
Source: Cell Death Dis. 2021 Oct 20;12(11):971. doi: 10.1038/s41419-021-04275-6 (PMC8528854; doi:10.1038/s41419-021-04275-6)
Supplement: Supplementary file 3 — Table S2 [file 41419_2021_4275_MOESM3_ESM.pdf]

ctsinfo	B7-H3 0 day vs CTL 0day log2FoldChange	B7-H3 0 day vs CTL 0day_pvalue	B7-H3 0 day vs CTL 0day_padj	CTL 0 day_1_CPM	CTL 0 day_2_CPM	B7-H3 0 day_1_CPM	B7-H3 0 day_2_CPM
TMED7-TICAM2	23.20851645	3.93E-13	3.35E-11	0.00078358	0.000878761	0.000630753	21.55160373
C7orf55-LUC7L2	23.03820372	1.48E-06	2.66E-05	0.00078358	0.000878761	20.31088975	0.000708911
RIPOR2	10.64415941	2.06E-12	1.42E-10	0.00078358	0.000878761	19.11245832	19.70843509
GSDMA	9.492581868	1.11E-10	5.43E-09	0.00078358	0.000878761	8.074274082	9.429225394
CACNA1I	8.371750727	1.82E-06	3.19E-05	0.00078358	0.000878761	4.605130464	3.403481777
SETDB2-PHF11	8.200997218	6.91E-06	0.000103271	0.00078358	0.000878761	3.659000387	3.474372879
EDARADD	8.131298552	9.50E-07	1.83E-05	0.00078358	0.000878761	3.53284971	3.261699574
CSAG3	8.014063299	1.24E-05	0.000168775	0.00078358	0.000878761	3.217473017	3.04902627
TNFSF12-TNFSF13	7.977940647	0.009175242	0.040917158	0.00078358	0.000878761	5.109733172	0.922293229
OSBPL10	7.622598628	3.10E-06	5.11E-05	0.00078358	0.000878761	2.775945648	1.98565975
TMEM171	7.614549761	2.77E-05	0.000331551	0.00078358	0.000878761	2.208267601	2.552788561
MIR6087	7.544021517	1.09E-06	2.07E-05	0.00078358	0.264507145	3.974377079	34.73734859
SBK1	7.386803575	0.000308133	0.002574796	0.00078358	0.000878761	2.208267601	1.843877547
SCUBE2	7.353250385	5.00E-05	0.000553731	0.00078358	0.000878761	2.523644294	1.418530939
CR1L	7.279041756	0.000187867	0.001701712	0.00078358	0.000878761	1.514438878	2.269224155
PLEKHH1	7.156577317	2.82E-05	0.00033599	0.00078358	0.000878761	1.892890909	1.560313141
SRD5A3-AS1	7.019767321	0.000360562	0.002941362	0.00078358	0.000878761	1.451363539	1.702095344
LIFR	6.992321918	0.001711531	0.010479046	0.00078358	0.000878761	1.325212862	1.772986445
ELAVL4	6.921078322	0.000154883	0.001441083	0.00078358	0.000878761	1.451363539	1.48942204
KCTD14	6.818650478	2.58E-08	7.06E-07	0.157499592	0.000878761	6.938917989	7.798730063
GPR85	6.567123503	5.66E-05	0.000613733	0.00078358	0.000878761	0.694459477	1.631204243
ADD2	6.554691465	0.006395832	0.030827739	0.00078358	0.000878761	1.009836169	1.276748736
TNFSF18	6.551501329	0.001953809	0.011650999	0.00078358	0.000878761	1.072911508	1.205857634
RHPN1-AS1	6.480652863	0.003681074	0.019681951	0.00078358	0.000878761	0.694459477	1.48942204

MDGA1	6.410263806	0.000217974	0.001916978	0.00078358	0.000878761	1.135986847	0.922293229
ANKRD53	6.409515245	0.008562012	0.038793843	0.00078358	0.000878761	1.135986847	0.922293229
CACNA1A	6.318042767	0.000148792	0.001392488	0.862721647	0.000878761	27.81685503	27.22289184
44078	6.308701927	6.08E-20	2.19E-17	0.627647628	0.000878761	21.76162254	19.28308849
ATL1	6.271296034	0.000732418	0.005268953	0.00078358	0.000878761	0.883685492	0.99318433
FAM26F	6.179024847	5.14E-08	1.31E-06	0.157499592	3.340171616	109.057891	91.02488308
TTC39A	6.167976515	5.33E-05	0.000584198	0.00078358	0.000878761	0.694459477	1.064075432
HEYL	6.156641313	0.001144262	0.007592113	0.00078358	0.000878761	0.946760831	0.780511026
LOC102723753	6.145284016	0.000812421	0.005699354	0.00078358	0.000878761	1.135986847	0.567837722
TRIL	6.127966097	7.24E-06	0.000107116	0.157499592	0.000878761	3.785151064	5.317541514
ZNF391	6.093449354	0.002231005	0.013081834	0.00078358	0.000878761	1.009836169	0.638728823
MAST4	6.052582602	7.42E-05	0.000780809	0.00078358	0.000878761	0.631384138	0.99318433
CTH	6.04486908	0.000575233	0.004314544	0.00078358	0.000878761	0.820610154	0.780511026
CLEC4G	5.967711618	0.002234391	0.013091554	0.00078358	0.000878761	1.009836169	0.496946621
DUXAP10	5.945753547	0.001896201	0.011377993	0.00078358	0.000878761	0.379082784	1.134966533
TDO2	5.840238228	0.001349165	0.008680247	0.00078358	0.000878761	0.883685492	0.496946621
CEP112	5.766097428	0.002679326	0.015192671	0.00078358	0.000878761	0.883685492	0.426055519
ASAH2B	5.719139681	0.001732327	0.010566022	0.00078358	0.000878761	0.505233461	0.780511026
TGFB2	5.717457765	0.000462381	0.003606824	0.00078358	0.000878761	0.505233461	0.780511026
TMEM154	5.714353958	0.002663324	0.015113812	0.00078358	0.000878761	0.5683088	0.709619925
TIMD4	5.571435284	4.57E-05	0.000511922	1.646301707	0.000878761	36.52125175	25.80506981
PLEKHB1	5.537675176	0.002468925	0.014240229	0.00078358	0.000878761	0.694459477	0.426055519
ANKRD22	5.522790651	1.76E-05	0.000227081	0.00078358	1.319020678	27.24917699	20.34645501
CARD14	5.346559204	0.001692333	0.010392367	0.00078358	0.000878761	0.757534815	0.213382215
KLHDC7B	5.32986884	5.38E-21	2.28E-18	1.254511677	0.176631017	24.9784648	21.69338593
LINC02099	5.258731691	0.003361741	0.018222435	0.00078358	0.000878761	0.5683088	0.355164418
PHYHD1	5.250888514	0.00731367	0.03417862	0.00078358	0.000878761	0.631384138	0.284273317

IL27	5.201750584	3.99E-26	3.84E-23	3.683609864	0.616011656	68.62659905	58.27319424
INPP4B	5.181390611	0.001600103	0.009913337	0.00078358	0.000878761	0.189856769	0.709619925
GCSAML-AS1	5.174684257	0.006707485	0.03192634	0.00078358	0.000878761	0.316007446	0.567837722
SOCS2	5.167822314	0.008348693	0.037986012	0.00078358	0.000878761	0.379082784	0.496946621
PROS1	5.161761455	0.000852752	0.005922071	0.00078358	0.000878761	0.442158123	0.426055519
DEFB1	4.931011541	6.95E-13	5.67E-11	1.097795665	0.176631017	17.85095155	13.47001817
GREB1	4.913820446	0.003316847	0.018041997	0.00078358	0.000878761	0.442158123	0.284273317
HAPLN3	4.909593268	7.44E-20	2.56E-17	1.332869683	0.4402594	24.28463608	18.50328637
SOCS1	4.890524273	4.01E-18	9.48E-16	1.254511677	0.352383272	17.66172554	20.84269272
CXCL11	4.885776219	0.000417953	0.003317645	68.32896485	2.285658083	807.8695664	851.6155098
FOXD4	4.825139075	0.001628054	0.010057553	0.157499592	0.000878761	1.135986847	2.552788561
SNX7	4.813992504	1.86E-16	2.86E-14	1.019437659	0.000878761	12.04802041	11.55595844
CXCL10	4.802065371	0.000132908	0.001267673	558.4582926	25.92433645	5775.998604	7203.174629
IFNB1	4.76543831	2.12E-09	7.77E-08	0.39257361	0.352383272	8.074274082	8.011403367
ST3GAL5-AS1	4.7535722	0.000639502	0.004704474	0.157499592	0.000878761	2.334418278	1.134966533
NEXN	4.732260428	4.81E-05	0.000534432	5.642560015	0.352383272	54.37157255	72.80587003
KCNA3	4.682957413	0.005051557	0.025480258	0.157499592	0.000878761	1.262137524	2.056550851
NUPR1	4.617397958	4.95E-08	1.27E-06	104.0602156	17.2245998	1304.903234	1055.427426
TNFSF10	4.611638124	3.27E-05	0.000381491	160.3996219	11.60052763	1608.043311	1735.131306
AK5	4.460053452	0.003876381	0.020566195	0.00078358	0.000878761	0.379082784	0.142491114
STAG3	4.452237393	2.83E-28	4.54E-25	1.332869683	1.670525189	24.03233472	28.07358506
ALK	4.437537244	1.41E-05	0.000188327	0.314215604	0.000878761	1.892890909	3.757937284
BATF3	4.366569083	0.007460467	0.034740232	2.038091737	0.000878761	18.54478027	14.8878402
IDO1	4.363213466	0.003058648	0.016972315	13.32164461	2.900790978	126.6559105	138.8763765
RET	4.331977577	1.57E-27	2.06E-24	2.821671798	1.758401317	34.3136149	39.41616128
ABTB2	4.229272065	7.18E-12	4.51E-10	1.724659713	0.4402594	14.12950658	18.57417747
APOBEC3A	4.228790242	0.001843992	0.01112546	63.70584249	2.109905828	522.4536596	456.681184

SMPD3	4.208436948	1.99E-06	3.47E-05	0.314215604	0.528135528	7.191219343	5.033977109
EIF5AL1	4.157276639	6.29E-09	2.07E-07	0.235857598	0.352383272	3.659000387	4.608630501
NT5C3A	4.075800521	2.04E-05	0.000255154	71.14985306	8.261234771	468.0827178	598.4633867
CLMP	4.04782631	0.000229	0.002001635	0.00078358	0.4402594	1.955966247	3.687046183
IFIT2	4.007953001	0.00048797	0.003773791	588.782841	42.26929622	3525.848978	4559.787241
IFIT1	3.982364224	1.08E-05	0.000151782	1193.863364	96.84037156	7776.622191	8442.421972
GBP4	3.942532459	0.000173493	0.00158964	26.1723576	2.373534211	153.8413814	195.8019309
PMAIP1	3.917266523	4.90E-17	8.84E-15	2.273165755	2.373534211	27.18610165	28.56982277
ETV7	3.903047628	0.000537358	0.004098631	18.80670503	1.934153572	128.1066433	118.3179571
ASCL2	3.868354387	3.06E-06	5.06E-05	11.28433645	2.197781955	84.14313232	72.23874122
HSH2D	3.854837117	0.001504728	0.009445291	21.62759325	1.143268422	140.7847863	120.6573635
APOBEC3B	3.824290943	3.78E-19	1.11E-16	11.9895585	3.515923872	91.1444949	83.79399074
FRMD3	3.77843569	5.22E-10	2.18E-08	2.351523761	0.4402594	13.87720522	16.94368214
KIAA0040	3.772834782	9.80E-62	1.41E-57	17.47461893	11.86415601	144.5062313	175.3852937
PIK3CD-AS1	3.743851347	0.000731247	0.00526578	0.78436364	0.000878761	4.163603095	4.325066095
CCL5	3.671094643	3.23E-25	2.59E-22	27.19101168	9.755128943	190.6773791	183.7504437
LINC00996	3.634816292	1.39E-06	2.52E-05	0.941079653	0.352383272	6.560465958	6.310016934
CH25H	3.616610941	0.003396927	0.018373709	0.00078358	0.528135528	1.325212862	3.757937284
HESX1	3.569893266	0.000340721	0.002804239	15.04552074	1.494772933	77.70944779	78.47715814
GBP1	3.553543012	2.29E-05	0.000281236	118.3997307	20.56389266	572.9770058	726.3509336
GCH1	3.528631901	2.20E-05	0.000272998	35.2618863	6.415836088	167.3395038	215.8641126
FOXC1	3.52244308	0.000467832	0.003643438	0.470931616	0.264507145	2.523644294	4.254174994
STAP2	3.467283159	0.002547308	0.014610572	0.00078358	0.176631017	0.883685492	0.567837722
USP30-AS1	3.455944941	7.85E-09	2.53E-07	3.605251858	1.23114455	26.74457428	15.38407791
CYP19A1	3.424212822	1.89E-06	3.31E-05	1.646301707	0.176631017	7.885048067	7.869621164
TMEM229B	3.423803712	7.60E-05	0.000796002	17.1611869	2.461410339	84.39543368	83.01418862
MDK	3.417910035	5.64E-05	0.000611673	17.1611869	2.81291485	98.71353552	70.46646368

IL1RN	3.362616926	3.94E-38	1.90E-34	517.0069074	300.0978551	2968.893739	3723.059571
CD274	3.362484125	8.23E-18	1.92E-15	13.79179264	4.921941916	68.75274973	84.92824836
ISG20	3.359365255	0.001565249	0.009757551	132.4258138	11.77627988	670.1761024	502.2641622
PDCD1	3.32874623	4.08E-13	3.47E-11	0.706005634	0.967516167	7.001993328	6.239125832
LINC01010	3.320646978	0.000352415	0.002887959	0.00078358	0.616011656	2.397493617	2.340115256
PRLR	3.312009022	1.28E-07	3.01E-06	2.586597779	0.791763911	10.91266432	16.02209782
CCL8	3.310128596	0.000559271	0.004223357	575.618696	66.08372684	2285.28322	2780.633269
DSP	3.299044031	5.12E-05	0.000564865	0.862721647	0.352383272	4.478979787	5.10486821
GBP5	3.289806179	3.46E-05	0.000399798	14.96716273	3.252295489	68.75274973	73.01854333
OTOF	3.287009031	0.002246193	0.013124162	21.86266727	5.712827066	118.8345685	94.56943815
AMIGO2	3.272140642	0.003555206	0.019103941	0.157499592	0.176631017	1.135986847	1.418530939
CDKL1	3.270443129	0.003324585	0.018075847	0.78436364	0.000878761	2.586719632	3.54526398
P3H2	3.252615741	4.24E-06	6.69E-05	0.00078358	0.528135528	2.082116924	1.702095344
LINC01296	3.247501817	0.004796452	0.024432617	0.39257361	0.000878761	1.009836169	2.056550851
LINC00537	3.230994267	1.26E-11	7.48E-10	3.840325876	1.494772933	21.19394449	18.78685078
PTPRU	3.230188848	2.74E-09	9.85E-08	3.683609864	1.319020678	20.81549246	16.58922663
HES1	3.221373889	9.52E-09	2.96E-07	1.959733731	5.712827066	40.24269672	15.88031562
IFIT3	3.211747346	6.37E-05	0.000685028	900.882779	126.8940073	3547.042292	4026.686158
SAMD4A	3.208461505	3.64E-15	4.52E-13	32.83278811	9.315748304	146.3354161	163.9718264
LINC01358	3.177338516	0.007577115	0.035159277	0.627647628	0.000878761	2.523644294	2.056550851
OASL	3.14650424	0.000180912	0.001647156	199.735341	32.0756654	804.4634981	826.7327332
ENDOD1	3.139687639	1.01E-14	1.16E-12	15.90745881	4.482561277	68.18507168	75.14527637
NFIX	3.122231009	9.93E-05	0.00098961	2.194807749	0.352383272	8.137349421	9.641898698
SERPING1	3.115261515	0.000321227	0.002670291	76.08640745	11.24902312	311.1512756	289.7326402
IL15RA	3.111078193	5.04E-13	4.23E-11	24.84027149	6.152207705	106.7241035	106.4082521
ELL3	3.105166344	0.000638983	0.004704474	1.411227689	0.967516167	6.560465958	9.783680901
SELL	3.102026217	7.89E-16	1.09E-13	21.15744521	6.591588344	90.63989219	99.24825084

RARRES3	3.101640209	0.000211576	0.001872015	18.414915	3.252295489	83.19700224	64.29893786
MEFV	3.1005593	3.55E-06	5.76E-05	2.821671798	0.528135528	11.03881499	11.98130504
GPBAR1	3.083844295	3.21E-21	1.40E-18	20.84401319	12.12778439	124.700575	97.1215178
C8orf31	3.068956206	7.78E-05	0.000811292	0.78436364	0.264507145	3.659000387	3.403481777
PLA2G16	3.064096639	7.05E-16	9.88E-14	17.39626092	7.294597366	91.20757023	72.80587003
DDX58	3.058520521	4.63E-05	0.000516474	152.9556114	33.13017893	562.8849516	670.9140924
AXL	3.055271104	0.000465667	0.003628531	50.30662346	7.382473493	171.7547775	210.0510423
ZNF702P	3.031405467	2.64E-06	4.47E-05	2.038091737	0.703887783	7.569671374	10.42170081
IGFBP4	3.027288417	1.35E-17	2.90E-15	134.93327	47.71761614	610.2545308	572.3754614
GLT1D1	3.024003776	2.06E-12	1.42E-10	4.937337961	3.428047744	31.22292331	22.75675245
IGF2BP3	3.01383187	2.11E-11	1.20E-09	3.213461828	1.670525189	13.24645184	18.36150417
RSAD2	2.996468313	0.000210652	0.001866122	1269.870629	170.744195	4105.259037	5046.171087
CCNA1	2.955866694	1.20E-09	4.66E-08	4.623905936	1.934153572	19.238609	21.40982153
ZNF618	2.933369415	4.45E-10	1.89E-08	2.97838781	1.143268422	10.28191093	15.0296224
SECTM1	2.926946582	2.41E-06	4.12E-05	238.0524059	73.90470221	993.3741369	889.3295757
TLR7	2.923963293	5.30E-32	1.53E-28	11.28433645	8.788491538	56.38998338	65.00784887
PRICKLE1	2.919908054	0.008033556	0.036815574	0.706005634	0.000878761	2.397493617	1.914768648
FN3K	2.919251447	1.40E-05	0.000188084	0.78436364	0.000878761	2.208267601	2.694570763
PLEKHA4	2.916746783	3.22E-09	1.13E-07	4.310473912	2.0220297	21.44624585	16.58922663
IRF4	2.894775554	0.001600125	0.009913337	1.332869683	0.703887783	5.425109865	6.664472441
HPSE	2.87955069	1.60E-18	4.21E-16	92.93337874	35.41495825	371.3251485	379.6225568
APOBEC3D	2.878435085	8.50E-07	1.65E-05	2.508239773	0.791763911	9.777308222	9.641898698
COL9A2	2.868913624	6.50E-08	1.63E-06	1.332869683	1.758401317	11.79571905	5.955561427
NEURL3	2.861470851	1.14E-10	5.56E-09	13.08657059	3.867428383	49.45169614	48.70289556
FAS	2.858984224	3.17E-11	1.75E-09	4.858979955	3.955304511	23.96925939	26.93932743
EXOC3L1	2.858340657	5.98E-06	9.09E-05	3.997041888	1.143268422	17.78787621	11.83952284
GAREM1	2.851090469	0.0016635	0.010245828	3.056745816	0.4402594	8.705027467	11.48506733

ANXA2R	2.826507033	1.26E-05	0.000171279	2.664955785	0.791763911	11.03881499	8.507641076
FPR2	2.825864263	8.09E-09	2.58E-07	3.605251858	1.494772933	12.93107515	16.02209782
USP18	2.814178225	0.001204974	0.007907677	120.2803229	17.57610431	377.3173057	393.3045394
ISG15	2.813278261	0.001744478	0.010635643	2593.964216	265.1231562	9271.444638	6640.795522
CKB	2.800343386	2.84E-20	1.10E-17	12.06791651	6.415836088	57.39918879	44.94566718
CDKN1C	2.797338289	3.32E-11	1.82E-09	3.135103822	3.515923872	17.85095155	18.85774188
TAP1	2.791407966	3.96E-41	2.86E-37	164.3958803	121.7971918	796.0114027	777.8887643
LOC100128993	2.7836609	3.78E-06	6.09E-05	2.038091737	1.494772933	7.948123405	11.48506733
GVINP1	2.769699215	9.12E-05	0.000919487	2.429881767	0.616011656	6.749691974	9.925463104
SAMD9	2.767258625	0.000371788	0.003015895	101.1609694	20.56389266	270.5938329	390.1853309
ACTA2	2.761532372	5.88E-11	3.01E-09	1.881375725	2.637162594	12.86799981	11.34328513
PTCH2	2.736765343	0.001149755	0.007618048	0.941079653	0.352383272	3.154397679	3.757937284
APOBEC3G	2.732724502	4.27E-05	0.000483122	49.5230434	12.56716503	162.1042507	165.9567772
LOC100506388	2.730895823	0.000749767	0.005364344	2.508239773	0.616011656	8.263500098	8.294967772
JAK2	2.728405678	3.33E-12	2.20E-10	24.9186295	10.72176635	74.74490688	113.9227088
GOLM1	2.72801792	0.003790829	0.020174194	23.35146938	2.725038722	65.97743483	71.4589391
RABGAP1L	2.724614271	2.34E-16	3.53E-14	54.06780775	20.73964491	195.7234061	197.5033174
IL15	2.724522122	2.57E-07	5.67E-06	4.310473912	1.670525189	14.44488327	17.15635544
TCN2	2.721615321	1.68E-12	1.23E-10	78.67222164	23.37592875	290.0841125	244.078771
IFIH1	2.717784982	8.73E-05	0.000888173	173.4070509	40.42389753	519.4260434	599.7394266
GBP3	2.715217685	2.24E-15	2.91E-13	34.63502225	16.69734304	114.6715962	154.1888544
VAMP5	2.70875831	0.000304849	0.002553329	50.54169748	12.74291729	200.1386798	127.1793448
TNFSF15	2.706494566	1.45E-18	3.88E-16	5.955992039	5.976455449	28.5737591	33.39041766
PPDPF	2.705313833	4.58E-25	3.48E-22	43.56783494	26.53946935	199.6340771	163.1920243
ITGA2	2.696159716	0.000104081	0.001026976	0.00078358	0.791763911	1.262137524	2.694570763
BATF2	2.690076965	0.000425291	0.00336595	54.06780775	10.98539473	165.6364697	168.0126192
EXT1	2.68803846	5.04E-22	2.70E-19	4.310473912	3.164419361	18.79708163	19.63754399

ATP10A	2.680726477	6.66E-09	2.18E-07	19.98207512	4.570437405	57.96686684	67.55992852
SLC27A3	2.678537792	5.95E-19	1.65E-16	16.29924884	10.37026184	73.92492748	61.67596711
LYSMD2	2.677501188	1.19E-18	3.24E-16	21.47087724	10.72176635	83.00777623	80.88745558
XRN1	2.674214812	1.62E-12	1.19E-10	21.70595125	8.788491538	60.86833241	94.71122035
KIF17	2.674002269	0.00194484	0.011611911	0.627647628	0.352383272	2.712870309	2.269224155
ADPGK-AS1	2.657780247	0.000268375	0.002286259	0.862721647	1.406896806	5.929712573	5.388432616
GRIN3A	2.654909028	3.00E-08	8.02E-07	7.679868172	2.725038722	23.90618405	28.42804056
BRIP1	2.651320839	5.36E-07	1.10E-05	2.900029804	1.319020678	8.768102806	12.47754275
TGM1	2.649673298	6.74E-06	0.000100937	2.508239773	1.494772933	11.98494507	7.940512265
PALM2-AKAP2	2.621473253	2.94E-21	1.32E-18	19.82535911	16.69734304	72.15881801	107.1880542
FXYD6	2.620840524	4.60E-12	2.98E-10	62.21704038	20.21238815	217.2951719	185.1682657
MAP2K6	2.60490376	0.003766397	0.020063813	0.941079653	0.000878761	1.82981557	2.836352966
SPN	2.580215983	2.42E-20	9.71E-18	7.053004124	12.4792889	49.57784682	42.74804304
TAGLN	2.579383985	1.30E-08	3.86E-07	3.918683882	1.758401317	15.39101335	11.62684954
FGL2	2.576894919	4.64E-12	2.99E-10	102.7281295	32.16354152	296.5177971	344.106115
GPR141	2.56221023	5.09E-15	6.07E-13	8.228374214	4.39468515	25.67229353	33.95754647
ARNT2	2.555226806	0.003245596	0.017775025	0.235857598	1.143268422	3.848226402	2.481897459
GPR161	2.546902918	1.13E-08	3.43E-07	2.743313791	2.81291485	12.74184913	13.04467156
TNK2-AS1	2.542896653	0.000710143	0.005142036	1.489585695	0.528135528	4.857431818	4.537739399
APOL6	2.542569922	7.51E-05	0.000788222	71.30656908	20.30026427	173.9624144	251.6641188
RPS6KL1	2.539917972	0.000688445	0.005012585	0.314215604	0.352383272	1.640589555	1.418530939
CMPK2	2.53670945	0.000100958	0.001002698	167.1384105	44.29044715	419.6408578	557.7718946
ARHGAP23	2.534172704	1.75E-09	6.56E-08	2.194807749	1.670525189	7.821972728	10.06724531
HERC5	2.525790081	0.00023405	0.002043305	122.395989	29.35150543	321.8740832	373.455031
TNFSF13B	2.523952326	0.001348093	0.008680247	76.24312346	15.6428295	169.1056133	252.4439209
CRHBP	2.523638457	1.77E-13	1.61E-11	13.00821258	6.679464471	42.89186094	47.35596463
CD40	2.519859547	1.34E-16	2.17E-14	30.0902579	15.46707725	112.0224319	95.49102247

METTL27	2.518987258	0.001704002	0.010450681	1.489585695	0.4402594	5.740486558	3.04902627
SP140	2.518325381	1.05E-07	2.54E-06	24.21340745	5.097694172	63.39134595	70.39557258
DYSF	2.517420962	6.79E-25	4.90E-22	33.38129415	24.95769905	131.2604102	134.339346
NCOA7	2.515973268	1.90E-12	1.37E-10	24.05669143	9.667252815	68.50044837	85.35359497
SIDT2	2.508954063	3.53E-19	1.06E-16	63.00062044	32.16354152	215.2136857	215.438766
TMCC3	2.506257044	1.08E-06	2.06E-05	1.489585695	5.449198683	13.56182853	17.65259315
ZBTB42	2.504177296	2.49E-10	1.14E-08	4.467189924	3.252295489	19.93243772	14.8169491
P2RY11	2.501800698	4.32E-09	1.47E-07	16.22089083	4.658313533	54.81309992	39.06170577
SLC22A16	2.495885666	1.12E-06	2.11E-05	5.564202009	1.758401317	14.0033559	19.07041518
LOC728743	2.483760836	3.25E-08	8.65E-07	2.743313791	2.197781955	13.37260252	8.507641076
JUP	2.475659434	0.006875868	0.032554348	110.9557201	14.41256371	268.3861961	285.9754119
RTP4	2.474217012	0.001150632	0.007620363	37.06412044	7.558225749	102.8134325	94.07320044
FGD2	2.470855008	1.29E-11	7.61E-10	98.73187119	31.19690412	279.2982297	293.4898686
SRGAP2D	2.461443164	1.95E-08	5.48E-07	6.034350045	3.076543233	14.69718463	25.52150541
KCNJ10	2.451951188	0.003062663	0.016985891	0.78436364	0.616011656	3.722075725	2.340115256
PPM1K	2.446109695	9.82E-09	3.03E-07	19.90371711	5.888579322	47.2440593	65.00784887
GMPR	2.444628408	0.000339809	0.002798962	119.7318168	28.64849641	324.460172	317.1674965
C5orf56	2.443239972	5.21E-06	8.09E-05	5.329127991	2.197781955	17.40942418	15.17140461
PKIB	2.440202849	1.19E-05	0.000162933	5.329127991	0.616011656	11.29111635	14.60427579
MB21D1	2.437079868	1.38E-11	8.13E-10	9.168670287	4.658313533	27.31225233	32.46883334
APOL1	2.429352132	0.000653596	0.004785411	115.7355585	25.57283194	288.6964551	316.6003677
CD1D	2.427251063	0.005555788	0.027519572	1.567943701	0.352383272	4.35282911	3.899719487
JADE2	2.421916694	1.40E-16	2.24E-14	37.53426847	18.63061785	121.2314314	118.1052838
C9orf66	2.419370918	0.001152062	0.007622836	1.332869683	0.528135528	3.785151064	4.183283892
44075	2.418055405	0.000212854	0.00187871	0.862721647	2.0220297	7.632746713	4.466848298
NFE2L3	2.417770858	1.06E-11	6.42E-10	12.38134853	7.11884511	40.30577206	42.67715194
PODXL	2.416896679	0.001887737	0.011341354	0.549289622	0.4402594	2.145192263	2.056550851

MCC	2.411318201	0.002446325	0.014149466	0.39257361	0.528135528	1.009836169	2.907244068
DENND1B	2.410639878	2.22E-18	5.61E-16	16.53432285	12.39141278	50.46090156	72.30963232
F2RL3	2.40768168	1.06E-07	2.55E-06	7.52315216	2.637162594	18.73400629	24.38724779
SIGLEC16	2.405440827	4.70E-22	2.61E-19	22.2544573	19.15787461	87.99072797	86.34607039
RBMS2	2.403606326	6.85E-17	1.20E-14	19.43356908	10.72176635	61.18370911	65.92943319
STOM	2.396864156	1.01E-22	6.05E-20	128.5079135	73.90470221	382.9940862	465.8261361
FUT4	2.391344278	3.41E-20	1.26E-17	28.05294974	21.61840619	97.38895341	110.0236983
HELZ2	2.375132158	1.64E-10	7.74E-09	135.9519241	45.78434133	338.5259725	412.1615723
TUFT1	2.366555498	1.50E-07	3.48E-06	2.351523761	2.461410339	10.66036296	9.003878786
PRUNE2	2.364724115	1.82E-06	3.19E-05	2.194807749	1.319020678	6.938917989	7.515165657
CBWD1	2.35788363	3.28E-27	3.64E-24	29.30667784	21.9699107	109.6255691	99.17735974
SMCHD1	2.356879445	3.53E-13	3.05E-11	43.0976869	22.32141521	108.2379116	159.2221226
IL4I1	2.355639075	3.08E-18	7.53E-16	458.3167609	251.4144803	1660.648143	1216.492009
SCIN	2.355180817	1.40E-10	6.70E-09	20.60893917	11.60052763	54.87617525	76.6339895
SMAD3	2.351430871	2.57E-08	7.06E-07	9.403744305	4.39468515	26.17689623	29.98764479
RBM43	2.327638158	3.47E-09	1.21E-07	6.112708051	4.043180638	16.7786708	23.96190118
NAA25	2.320583995	1.95E-20	8.05E-18	17.94476696	13.0944218	57.58841481	65.92943319
TRIM5	2.314607964	3.64E-11	1.94E-09	35.81039234	16.17008627	82.69239954	123.7765719
MCOLN2	2.311585515	7.77E-05	0.000811222	8.620164244	1.846277444	20.43704043	20.98447492
FLJ32255	2.301757107	7.10E-13	5.75E-11	14.02686666	7.294597366	40.81037477	42.96071635
CD72	2.294920553	1.29E-08	3.84E-07	7.13136213	3.955304511	26.80764962	16.30566223
C17orf97	2.293508102	0.003413713	0.018457586	0.00078358	1.23114455	2.208267601	2.481897459
PI4K2B	2.2916883	3.84E-16	5.59E-14	22.72460533	17.48822819	70.77116056	86.06250598
SAMD9L	2.268388893	0.001463894	0.009257428	193.1532685	44.29044715	369.4959637	542.7429811
ADAMTSL4	2.267008094	1.87E-14	2.09E-12	30.32533192	15.11557274	92.40600167	81.38369329
DUSP5	2.261640916	2.57E-18	6.39E-16	27.19101168	44.46619941	145.8938887	126.3995427
SPATS2L	2.257492573	0.000334743	0.002761963	103.1199195	27.76973513	239.1823144	258.4696645

SP110	2.251552914	0.002459053	0.014211692	127.8026914	28.47274416	289.8948865	301.5714542
PML	2.248101812	1.21E-11	7.19E-10	198.7166869	73.02594093	507.5048044	518.4982244
NCF1	2.247051976	2.06E-19	6.33E-17	524.607634	335.5998107	1805.595271	1431.079372
LILRA5	2.246263372	0.003035617	0.016881307	122.552705	25.30920356	298.7254339	257.8316446
SLFN12	2.240159415	1.87E-07	4.23E-06	8.071658202	2.900790978	20.31088975	21.05536602
CD38	2.235474559	4.00E-10	1.73E-08	72.63865518	26.18796484	190.109701	179.6387598
C1GALT1	2.235219847	5.57E-13	4.64E-11	24.21340745	12.21566052	59.10222293	77.76824712
OAS1	2.234976406	0.000347382	0.002854826	637.4431627	154.6628636	1436.604541	1527.420379
MX1	2.230727309	0.000314945	0.00262412	1489.743194	389.3800009	3330.189278	3683.289663
CTSC	2.227123838	5.57E-29	1.00E-25	493.2644316	346.7600789	1581.740894	1542.023946
FAR2	2.225398619	3.31E-09	1.16E-07	5.564202009	10.45813797	24.22156074	35.3753685
HEG1	2.222762522	8.37E-17	1.42E-14	13.08657059	13.88530695	46.80253193	53.31081715
NUB1	2.22217487	6.87E-20	2.42E-17	84.00056606	49.47513869	242.7145333	252.514812
PDGFRL	2.221226732	0.004300257	0.022427541	5.642560015	0.879640039	12.04802041	12.19397835
CD47	2.220360367	1.01E-17	2.25E-15	163.5339422	86.73461686	459.8198485	467.1021759
ANO5	2.208515723	1.23E-05	0.000166982	1.724659713	2.197781955	5.172808511	9.287443192
GSDMD	2.208181937	0.00327036	0.017874183	122.474347	26.27584096	296.2024204	248.970257
SSTR2	2.197253895	0.001460806	0.009241952	0.862721647	1.934153572	5.298959188	4.821303805
PARP15	2.195182479	2.21E-08	6.14E-07	10.34404038	5.185570299	27.12302631	29.56229819
ARL5B	2.194113526	0.000111889	0.001092721	3.291819834	1.143268422	7.31737002	8.932987685
FAM122C	2.188326954	0.000991323	0.006743593	3.135103822	0.879640039	5.614335881	9.074769887
CD200R1	2.187994228	0.00169489	0.010403639	3.840325876	0.703887783	5.866637235	10.77615632
BAK1	2.182942808	5.77E-22	2.98E-19	76.47819748	51.32053738	229.4687122	231.8146104
NTNG2	2.180706166	6.23E-05	0.000670126	5.172411979	1.758401317	12.11109575	12.97378046
FAM129A	2.178294349	8.96E-12	5.55E-10	20.92237119	18.01548495	63.26519528	77.05933611
ATXN7L1	2.175183718	4.06E-10	1.75E-08	12.61642255	6.767340599	30.33986858	39.55794348
APOL3	2.174545038	0.001624764	0.010045181	104.2169316	24.60619454	239.6869171	222.0316384

TMEM268	2.157416188	5.93E-14	5.90E-12	57.358844	28.03336352	144.3170053	158.8676671
FFAR2	2.155232532	8.25E-11	4.15E-09	53.75437572	25.1334513	146.6507928	132.4252863
NCF1C	2.154889669	1.30E-21	6.07E-19	228.4927292	176.5440194	816.0693604	612.9251714
PIWIL4	2.151702557	8.24E-06	0.000119437	8.228374214	3.164419361	19.11245832	21.26803932
IRF7	2.149672933	7.44E-10	3.01E-08	307.7910313	113.2732074	809.9510525	671.7647856
PLSCR1	2.146976988	6.97E-10	2.83E-08	135.32506	50.52965223	299.86079	355.3778001
C4orf32	2.14414964	4.03E-06	6.44E-05	3.918683882	2.109905828	10.34498627	10.91793852
SLC8A1	2.139488979	8.07E-09	2.58E-07	16.45596485	9.315748304	36.39510107	54.30329257
EMBP1	2.134299119	0.007855337	0.036148075	0.862721647	0.528135528	2.523644294	2.340115256
GCNT1	2.133680083	2.46E-26	2.53E-23	71.07149506	56.1537244	205.1216316	239.2581761
DDO	2.129116087	5.07E-06	7.88E-05	6.661214093	2.109905828	14.63410929	16.02209782
PNPT1	2.127554477	4.23E-09	1.45E-07	66.3700147	24.51831841	142.0462931	174.3219272
MVB12B	2.117220598	5.60E-10	2.32E-08	27.89623373	11.33689924	66.86048957	68.62329505
NMI	2.109510125	5.49E-10	2.28E-08	101.9445494	38.66637498	234.7039653	247.8359993
CBWD2	2.109509895	1.21E-16	1.99E-14	41.29545276	24.43044228	111.9593566	113.6391444
IL3RA	2.1083717	0.000394533	0.003161301	1.097795665	2.725038722	6.560465958	6.451799136
AKAP7	2.105462701	3.64E-07	7.81E-06	6.112708051	3.779552255	16.27406809	17.65259315
C21orf91	2.105044006	2.43E-06	4.15E-05	3.213461828	3.164419361	9.588082206	12.26486945
EIF2AK2	2.103555447	3.96E-08	1.04E-06	88.85876243	29.61513382	166.4564491	239.6835227
PKD2L1	2.103211884	2.85E-06	4.77E-05	7.209720136	2.988667105	18.98630764	15.88031562
IFIT5	2.098638269	4.00E-07	8.49E-06	41.68724279	12.30353665	85.27848841	98.89379533
OAS2	2.094536439	0.000804787	0.005662549	537.144915	144.9964896	1043.203654	1275.756969
EPS8L1	2.091829375	1.13E-05	0.00015651	5.094053973	2.637162594	15.45408869	10.70526522
ARAP2	2.091696107	8.05E-07	1.58E-05	5.955992039	4.921941916	13.87720522	23.18209906
MIR155HG	2.086397657	2.22E-05	0.000274446	2.273165755	1.406896806	4.605130464	7.940512265
APOBEC3F	2.086042966	2.46E-05	0.000299338	14.34029869	3.428047744	28.2583824	31.90170453
PARP11	2.070467356	9.84E-09	3.04E-07	9.247028293	6.152207705	23.08620465	28.49893167

DHX58	2.060052105	9.32E-09	2.91E-07	141.7504165	46.39947422	312.4758577	310.9999707
BLZF1	2.043574249	4.41E-07	9.18E-06	22.48953131	10.28238571	39.29656664	68.76507725
EPDR1	2.043312298	3.82E-14	4.05E-12	10.6574724	6.855216727	28.32145774	29.20784268
GBP2	2.0371528	2.24E-15	2.91E-13	84.94086213	47.19035937	210.1045833	221.1809452
SCIMP	2.035712029	2.00E-12	1.41E-10	55.32153584	28.38486803	136.6848893	136.1825147
SLC44A2	2.035527159	1.54E-12	1.15E-10	12.30299053	21.0032733	58.66069556	49.41180657
SSB	2.030454801	5.70E-11	2.93E-09	179.7540494	83.57107627	388.4185653	467.9528691
AMER1	2.0277689	0.001469199	0.009274723	1.176153671	2.285658083	3.595925048	7.65694786
CBR1	2.027635312	3.63E-09	1.26E-07	308.5746114	112.3944462	727.7008111	634.1925018
TMEM110	2.027275673	4.37E-17	8.08E-15	52.1872156	33.48168344	138.0094714	139.6561786
ZNRF2	2.026870184	2.78E-13	2.44E-11	34.47830624	32.0756654	89.88298813	126.3286516
FAM46A	2.026510821	2.33E-13	2.08E-11	57.51556001	30.66964735	127.4128145	158.8676671
HERC6	2.021382384	0.004969925	0.02517409	63.00062044	13.79743082	110.1301718	138.1674655
SLC18B1	2.017166373	8.20E-14	8.00E-12	9.247028293	8.70061541	26.36612225	31.47635792
RAB40B	2.012704412	0.000107861	0.001059597	1.646301707	1.846277444	5.172808511	6.026452528
SULT1C2	2.009392788	0.004189909	0.021947216	0.00078358	0.616011656	0.820610154	1.064075432
MARCKS	2.007903694	6.74E-13	5.52E-11	782.7972639	370.1351289	1669.983293	2020.751554
GRAMD1B	2.007565996	2.96E-09	1.05E-07	7.209720136	6.240083833	18.79708163	24.31635668
MGC16275	2.005560644	0.00366188	0.01959384	2.116449743	1.319020678	5.046657834	5.955561427
C3orf38	2.002708253	9.57E-16	1.32E-13	26.87757965	18.27911334	65.78820882	78.40626704
MX2	1.998863347	0.000742761	0.005329956	587.3723969	164.1534854	1116.686424	1272.56687
FOLR2	1.996567715	9.21E-09	2.89E-07	43.25440291	17.57610431	95.05516588	98.04310212
IFI35	1.994638977	0.001166566	0.007704674	491.9323455	133.7483452	1045.600517	932.9984942
EPSTI1	1.994286245	1.48E-08	4.31E-07	298.1529966	98.15851347	609.3084007	645.9604247
TAGAP	1.991724295	4.97E-13	4.20E-11	20.76565518	13.0944218	54.43464788	52.67279724
MNDA	1.991645068	4.10E-10	1.76E-08	183.7503077	77.94700409	391.2569555	436.6190023
HLA-C	1.990032126	4.26E-19	1.21E-16	2168.558601	1611.385434	6519.782996	5386.023027

SERPINB9	1.989608338	2.20E-09	8.06E-08	30.16861591	13.88530695	59.41759963	80.17854457
LOC101928101	1.987841975	0.003732808	0.019929022	0.627647628	1.143268422	2.775945648	2.765461865
WARS	1.986188006	3.70E-29	7.62E-26	163.7690162	132.0786988	441.0864729	491.2051504
ABHD17C	1.985738302	0.006217114	0.030208474	0.78436364	0.4402594	1.892890909	1.98565975
C1R	1.984792843	0.000145044	0.001362711	2.429881767	2.461410339	7.443520697	7.940512265
RNF138	1.984494962	8.71E-10	3.45E-08	16.84775488	10.72176635	40.74729943	46.15081591
CHST12	1.97649293	7.37E-17	1.27E-14	24.52683947	19.94875976	74.61875621	64.29893786
ALDH1A1	1.96791377	3.17E-12	2.12E-10	63.94091651	33.6574357	129.6835267	174.7472738
PARP10	1.958872922	2.92E-08	7.85E-07	310.2201295	116.6125003	738.8020707	576.3453631
CD2AP	1.954189101	2.40E-09	8.71E-08	23.50818539	15.02769661	51.02857961	68.05616623
RIN1	1.952995386	2.24E-11	1.27E-09	36.67233041	21.70628232	98.27200815	81.10012889
TMEM173	1.951277877	2.80E-17	5.46E-15	15.75074279	13.62167856	43.648765	46.71794472
TRANK1	1.950073446	1.25E-08	3.75E-07	21.78430926	9.843005071	47.81173734	49.48269767
PDCD1LG2	1.948817756	6.94E-05	0.000738172	12.85149657	2.900790978	23.46465668	25.0252677
UBE2S	1.945321076	8.67E-13	6.91E-11	69.89612497	50.09027159	197.552591	169.0759857
DAPP1	1.945216012	1.79E-09	6.66E-08	23.11639536	13.27017405	50.01937419	61.67596711
DOPEY1	1.943511833	1.12E-05	0.000155684	5.172411979	4.746189661	13.30952718	17.08546434
PRKCE	1.94347941	0.001073763	0.007190476	2.351523761	1.494772933	4.542055126	7.302492353
ATP13A2	1.941420815	8.52E-13	6.83E-11	139.3996763	72.93806481	349.9426088	297.1053148
HIST2H2BE	1.938759612	1.35E-06	2.47E-05	30.71712195	12.30353665	78.52942719	52.10566842
ST3GAL5	1.935501513	2.72E-15	3.48E-13	69.42597693	42.35717234	171.1870995	168.6506391
SLC6A12	1.935284652	0.000102125	0.001010817	5.094053973	2.637162594	12.80492447	10.70526522
ADGRG6	1.934593034	0.000896463	0.006178062	0.941079653	0.791763911	2.712870309	2.552788561
CBWD5	1.933019651	1.24E-12	9.41E-11	19.27685307	12.56716503	49.01016877	47.71042014
RGS1	1.931720526	4.49E-16	6.41E-14	76.71327149	105.0128514	318.4049395	230.3967884
RASGEF1B	1.930905882	1.54E-19	4.85E-17	37.8477005	47.2782355	121.7991094	136.1825147
PAPD7	1.927313182	1.04E-19	3.40E-17	36.67233041	29.26362931	97.7043301	101.7294394

ADIRF	1.924869053	1.03E-05	0.000145047	12.38134853	6.415836088	31.28599865	25.3797232
SENCR	1.918775693	6.62E-05	0.000708994	4.780621949	1.934153572	9.651157545	10.63437412
RICTOR	1.916549264	7.26E-09	2.35E-07	14.10522467	8.70061541	29.64603985	39.06170577
UBE2L6	1.914611324	0.001094047	0.007310323	456.2010947	125.1364847	885.5153081	855.0182826
FAM89A	1.908442657	0.000289653	0.002448729	1.019437659	2.549286466	5.172808511	5.388432616
OSBPL1A	1.905085523	1.04E-12	8.05E-11	36.82904642	26.27584096	78.08789983	110.4490449
FBXO6	1.90207675	2.64E-07	5.79E-06	59.70958418	20.73964491	122.9344655	115.9785508
TFAP2C	1.902026792	0.001622614	0.010041139	0.627647628	1.143268422	2.523644294	2.694570763
ATF5	1.900995141	4.81E-17	8.78E-15	444.6041099	277.4258141	1085.401056	1056.349011
NAP1L5	1.900001352	0.009169195	0.040907196	0.78436364	0.791763911	1.451363539	3.261699574
NCF1B	1.899293801	2.20E-10	1.03E-08	215.6420162	117.5791377	584.3305667	399.6138474
STAMBPL1	1.897243979	3.41E-05	0.000394685	1.097795665	1.846277444	3.785151064	4.892194906
SLC15A4	1.891802737	3.07E-25	2.59E-22	42.39246485	45.25708456	121.6729587	136.9623168
PAQR8	1.890859838	9.96E-06	0.000141327	28.36638177	7.90973026	53.99312052	53.02725274
NRIP1	1.889143275	2.09E-11	1.20E-09	27.03429566	20.12451202	58.91299692	80.39121787
GIMAP4	1.887204442	6.20E-08	1.56E-06	91.75800865	32.60292216	175.5392978	190.4142072
LGALS3BP	1.88630169	1.60E-09	6.02E-08	570.9172156	228.2151826	1215.083952	1130.288429
ENPP4	1.886144326	1.11E-07	2.66E-06	17.70969295	9.227872177	33.61978618	45.8672515
NCOA2	1.882918312	4.26E-09	1.46E-07	17.47461893	14.0610592	37.65660784	55.15398578
RPH3AL	1.882121321	0.006670596	0.031801577	1.489585695	1.055392294	2.965171663	4.537739399
SASH1	1.880897399	2.06E-11	1.19E-09	20.68729717	29.35150543	60.93140775	85.84983268
STAT1	1.880810363	1.28E-07	3.01E-06	787.5771023	260.9929782	1366.65399	1707.909123
GRK5	1.878538819	0.00085622	0.005940437	1.489585695	1.582649061	4.226678433	4.750412703
NUDT7	1.875507013	0.000113798	0.001107681	1.489585695	1.23114455	3.974377079	3.970610588
LINC01504	1.870485142	4.70E-05	0.000523726	5.407485997	2.81291485	13.05722582	10.84704742
ID1	1.866472677	0.002055212	0.012170193	4.232115906	4.921941916	21.13086915	5.033977109
ZNFX1	1.865096952	1.91E-12	1.37E-10	201.3808591	108.3521443	396.1137566	502.5477266

PPA1	1.862988398	9.26E-13	7.30E-11	21.39251923	13.62167856	50.5239769	50.8296286
ACSM5	1.858694509	0.008478972	0.038490104	3.448535846	1.055392294	6.371239943	6.664472441
PDE4B	1.84418274	6.55E-05	0.000702375	6.7395721	2.461410339	11.60649304	14.8169491
CASP1	1.836630257	9.67E-12	5.96E-10	122.787779	63.00806237	263.340169	264.0700615
EIF4E3	1.833211451	2.56E-14	2.84E-12	27.11265367	22.76079585	63.76979798	77.76824712
PSMA4	1.831203297	1.40E-12	1.05E-10	174.8174951	93.67683096	378.2634358	380.9694877
AIDA	1.828305349	1.72E-13	1.57E-11	23.97833343	23.81530939	56.38998338	78.90250475
NAMPT	1.828103222	2.79E-13	2.44E-11	144.1011567	91.12842325	301.3115228	363.6011679
MYO10	1.827358038	4.33E-08	1.13E-06	6.504498081	10.19450958	24.78923879	22.11873254
C17orf67	1.826798408	0.005499888	0.027312074	3.448535846	1.319020678	6.749691974	6.735363542
ZNF684	1.821753665	0.006285168	0.030457105	2.664955785	0.879640039	3.280548356	6.806254643
LOC101927181	1.821641262	0.003138443	0.017352825	1.332869683	1.582649061	3.722075725	4.466848298
PLEKHN1	1.821181709	0.001971771	0.011743553	1.411227689	3.955304511	8.831178144	6.09734363
SNX10	1.81861437	3.29E-12	2.18E-10	44.74320503	36.2058434	95.37054258	132.2835041
RAB38	1.81615109	1.62E-07	3.71E-06	3.683609864	1.494772933	7.001993328	7.65694786
C2	1.815946793	2.00E-12	1.41E-10	77.96699959	44.81770392	173.0162843	170.5646988
ARHGEF19	1.814115736	0.001325056	0.008570994	3.76196787	1.934153572	8.831178144	7.089819049
LY86	1.80727362	9.06E-09	2.85E-07	84.5490721	37.69973757	174.0254897	165.8858861
PANX1	1.806594197	6.16E-13	5.08E-11	18.88506304	14.6761921	43.58568966	49.90804428
MT2A	1.804769994	1.34E-10	6.48E-09	129.5265676	118.5457751	394.5999485	291.8593733
MFN1	1.804707551	1.98E-10	9.28E-09	10.42239838	9.667252815	24.78923879	31.12190242
GTPBP1	1.802786887	4.08E-14	4.26E-12	75.61625941	47.2782355	159.7704632	181.4819284
CASP7	1.799994459	7.12E-11	3.63E-09	44.27305699	30.14239058	87.10767323	119.5231058
GPR155	1.799347622	2.92E-07	6.34E-06	18.33655699	8.349110899	36.45817641	37.50210154
PSMB9	1.797257953	0.004598377	0.02360487	144.8847367	41.47841107	285.4165375	228.057382
TMEM62	1.797005722	1.21E-08	3.67E-07	32.12756606	16.2579624	70.89731124	62.66844253
HS3ST3B1	1.794996676	0.008938135	0.040119627	0.314215604	1.934153572	3.406699033	2.694570763

LNPEP	1.793550554	7.33E-06	0.000108271	7.444794154	4.921941916	12.55262312	21.76427703
KIAA1522	1.788908922	9.68E-14	9.25E-12	17.39626092	13.44592631	42.57648425	42.18091423
CD80	1.78879345	0.000160233	0.001484159	18.25819899	4.658313533	31.28599865	31.75992233
IRF1	1.788641016	9.57E-15	1.10E-12	74.83267935	49.73876708	158.7612578	183.8213348
MFSD14A	1.7867743	1.23E-12	9.41E-11	23.6649014	28.73637254	65.47283212	78.33537593
CPEB3	1.784876113	0.000224163	0.001966227	3.683609864	2.900790978	6.938917989	11.20150293
MOV10	1.779391176	1.16E-08	3.52E-07	118.2430147	49.91451933	235.8393214	222.6696583
HEXDC	1.778037642	4.40E-10	1.87E-08	29.77682588	24.69407066	88.24302932	59.62012517
FPR1	1.777297018	1.86E-05	0.000236522	14.18358267	4.921941916	23.96925939	28.28625836
IFI6	1.777296766	0.004914406	0.024927871	3359.443577	997.7464334	6652.178131	5183.558042
RFK	1.777214103	1.14E-09	4.44E-08	14.02686666	12.21566052	32.29520407	39.34527018
C19orf38	1.765487821	1.60E-06	2.87E-05	27.11265367	11.95203214	54.87617525	50.6878464
PPP2R2A	1.763291486	3.51E-14	3.81E-12	46.07529113	31.02115186	95.30746724	113.1429067
BLVRA	1.762105785	4.95E-11	2.58E-09	165.7279664	89.98603359	352.6548483	336.2372027
ELF1	1.755177821	7.55E-10	3.04E-08	37.8477005	38.31487047	88.99993339	115.7658775
SMYD2	1.754978068	3.09E-10	1.39E-08	13.79179264	10.45813797	30.59216993	34.59556638
GPR180	1.753584823	1.01E-07	2.45E-06	12.30299053	8.612739282	22.39237592	33.95754647
KAT2B	1.751343068	6.79E-10	2.77E-08	18.72834702	13.35805018	35.9535737	50.26249979
C18orf32	1.748354213	4.91E-10	2.06E-08	26.40743162	13.62167856	51.47010698	55.65022349
CYP2U1	1.748086346	0.000636151	0.004688608	5.250769985	2.0220297	9.84038356	9.641898698
AAED1	1.74758186	9.61E-11	4.78E-09	14.4970147	10.98539473	29.96141654	38.28190366
GGACT	1.745978726	6.07E-08	1.53E-06	6.661214093	4.306809022	15.45408869	13.82447368
TRAFD1	1.738154598	2.90E-20	1.10E-17	120.7504709	99.47665539	287.939551	295.9710572
MASTL	1.738038793	4.31E-07	9.01E-06	28.20966575	15.11557274	50.83935359	64.36982896
SYTL3	1.737398714	0.000279565	0.002378776	4.702263942	2.109905828	9.588082206	8.507641076
LMNB1	1.737342418	3.57E-11	1.91E-09	21.86266727	26.62734547	57.46226413	71.17537469
RNASET2	1.737261095	7.94E-09	2.55E-07	297.7612065	144.9964896	644.2521383	526.2253544

TRIM38	1.735049016	1.57E-07	3.61E-06	95.04904491	36.99672855	170.6194214	178.9298488
TFEC	1.734987807	1.18E-11	7.05E-10	105.9408077	65.38071782	204.4278029	249.679168
GIMAP5	1.733190573	3.64E-06	5.87E-05	87.84010835	27.59398288	157.9412784	146.887071
TMEM140	1.73245172	9.56E-15	1.10E-12	87.84010835	61.60204432	198.3725704	196.2272775
TMPPE	1.731384465	0.004317632	0.022501897	2.351523761	0.879640039	3.848226402	4.750412703
RUBCN	1.730664518	1.59E-12	1.18E-10	96.45948902	57.20823794	190.7404544	215.0843105
IGSF8	1.729081334	2.78E-10	1.26E-08	36.04546636	24.69407066	86.16154315	73.65656324
SELENOO	1.728014155	4.79E-10	2.01E-08	102.6497715	61.86567271	236.4700748	195.8019309
TMPRSS13	1.72776991	0.000624192	0.004616976	3.056745816	2.373534211	6.875842651	7.444274556
ATP8A1	1.726383007	2.78E-05	0.000331605	3.056745816	4.131056766	8.200424759	10.70526522
OSBPL5	1.72086854	3.37E-10	1.49E-08	21.86266727	17.8397327	53.55159315	50.40428199
TAP2	1.715777756	9.21E-06	0.000131704	222.2240887	61.77779658	373.3435594	367.7837429
VCPIP1	1.714709205	9.99E-08	2.42E-06	19.19849506	15.6428295	38.91811461	52.24745063
TTC9	1.713794956	0.000325094	0.002696224	12.30299053	3.779552255	21.50932118	20.48823721
GPRIN1	1.713480819	0.001062913	0.007137689	2.429881767	2.285658083	6.118938589	6.168234731
RBCK1	1.711955104	1.47E-11	8.64E-10	161.65335	96.04948641	343.7612256	326.7377952
KCNMB1	1.711278685	1.20E-05	0.000163789	8.855238263	4.218932894	17.91402689	16.16388003
PSME2	1.710472799	1.96E-06	3.43E-05	343.5222821	116.5246242	639.6476386	554.8653594
C19orf66	1.71014102	1.21E-09	4.66E-08	127.4892594	65.20496556	258.5464433	242.1647112
LYN	1.709557534	1.74E-17	3.59E-15	368.440128	266.8806788	736.1529065	918.2531451
EXOSC9	1.702166995	1.02E-12	7.93E-11	19.51192708	15.81858176	42.8287856	48.70289556
PAPD5	1.700886071	2.07E-06	3.59E-05	5.799276027	5.624950938	12.04802041	17.58170205
OAS3	1.699639301	0.00861109	0.03893065	654.44685	170.2169382	896.679643	1237.972012
CD86	1.697930361	1.69E-17	3.54E-15	134.854912	96.22523866	296.7700984	299.0193745
GIMAP6	1.6889617	3.22E-06	5.29E-05	51.48199355	19.50937913	75.37566027	107.2589453
OLFML3	1.68631583	4.56E-06	7.15E-05	3.840325876	6.064331577	9.777308222	15.59675121
SIGLEC11	1.684348837	4.25E-06	6.71E-05	23.5865434	10.10663345	39.86424469	46.43438031

STX11	1.682307601	3.58E-14	3.86E-12	54.92974581	41.9177917	123.8805956	123.138552
CNP	1.680338701	1.40E-09	5.30E-08	282.8731854	140.3390548	537.0871381	540.6871391
LINC00623	1.680066927	6.77E-06	0.000101281	4.075399894	5.888579322	13.62490387	11.62684954
FAM8A1	1.678335789	5.26E-08	1.34E-06	14.4970147	10.37026184	27.87993037	35.5880418
PCGF5	1.673056311	1.03E-13	9.70E-12	45.6835011	37.43610919	89.31531008	122.0042944
DYNLT1	1.670414207	5.94E-07	1.21E-05	224.1830389	91.47992776	414.2163787	383.5215674
PHLDA2	1.666748572	8.80E-07	1.70E-05	6.504498081	6.415836088	18.04017757	14.46249359
BCL2	1.663674523	2.22E-05	0.000274544	3.213461828	4.570437405	8.263500098	11.34328513
CLIC4	1.662784016	3.39E-10	1.50E-08	39.80665065	34.44832084	80.10631066	107.2589453
CPED1	1.659877666	0.000160336	0.001484159	5.329127991	3.691676127	8.831178144	13.96625588
WDFY1	1.657877361	4.80E-14	4.88E-12	39.80665065	36.64522404	86.91844722	105.1322123
BAG1	1.655297869	8.68E-11	4.35E-09	95.98934098	57.91124696	204.3647275	180.4185619
ABRAXAS1	1.653496705	0.001833734	0.011090784	5.642560015	2.109905828	8.57887679	10.91793852
IFI44	1.652248713	0.007013716	0.033109238	277.779915	72.49868417	434.0851104	440.7306862
LRRC4	1.651038724	3.62E-06	5.86E-05	7.209720136	5.53707481	15.01256132	16.87279104
SCLT1	1.650984221	3.08E-05	0.000361895	10.03060835	5.361322555	16.14791741	22.47318805
TRIM21	1.65059932	1.35E-08	3.99E-07	121.5340509	56.59310504	221.3319936	223.165896
DTX3L	1.647728815	1.94E-08	5.48E-07	130.3101476	67.2261165	211.8076175	281.4383814
RNF19B	1.646376721	2.96E-17	5.70E-15	134.4631219	105.1007276	295.950119	300.0118499
BBX	1.645431039	6.87E-07	1.37E-05	12.22463252	11.24902312	26.49227293	31.97259563
SEMA4D	1.644085341	2.32E-13	2.08E-11	32.91114612	24.69407066	66.86048957	76.4922073
APOL2	1.643097863	2.57E-09	9.32E-08	114.9519784	58.70213211	215.3398364	215.7223304
NAPA	1.642982842	5.57E-11	2.87E-09	422.4287941	236.9149192	821.3046135	814.5394637
DCK	1.642186926	3.12E-07	6.77E-06	18.49327301	13.53380244	32.23212873	47.56863794
PSD3	1.642171377	5.49E-06	8.43E-05	6.191066057	6.503712216	15.45408869	16.02209782
CSRNP1	1.639179333	1.14E-15	1.55E-13	45.6051431	44.55407554	115.1131235	107.8260741
CHRNB1	1.638660117	2.00E-06	3.50E-05	8.228374214	5.624950938	15.07563666	19.35397959

FPR3	1.636571273	2.97E-12	1.99E-10	1155.076151	683.7650289	2047.299968	2503.661736
NBN	1.633946473	2.15E-08	5.99E-07	28.20966575	20.47601653	48.50556607	72.09695901
SAV1	1.632130689	3.65E-07	7.81E-06	6.896288112	8.612739282	17.03097215	21.19714822
CPEB2	1.629449709	2.74E-06	4.62E-05	14.41865669	10.8975186	26.05074556	36.43873502
ADA2	1.62841431	5.06E-10	2.11E-08	325.5782987	170.744195	549.9545072	671.6230034
PHACTR2	1.627283462	6.33E-07	1.28E-05	15.20223675	18.19123721	37.53045717	44.52032058
HLA-H	1.62705962	5.72E-10	2.36E-08	345.2461582	212.2217273	774.1242602	590.240019
FMR1	1.626791924	3.27E-06	5.35E-05	26.01564159	13.00654567	39.35964198	56.85537222
SDC3	1.626308241	2.60E-11	1.45E-09	97.24306908	60.72328305	207.5815698	179.4260865
THRB	1.621848744	0.00289672	0.016208825	1.411227689	1.055392294	3.469774371	2.552788561
ZNF296	1.620820324	3.60E-05	0.000414854	7.679868172	5.712827066	17.53557486	15.17140461
DLAT	1.613640209	4.07E-11	2.14E-09	23.27311137	17.57610431	46.1087032	53.45259935
BCL2L13	1.612233396	5.11E-17	9.10E-15	66.3700147	53.6053167	139.1448275	152.6292502
EVL	1.611421007	1.34E-07	3.13E-06	62.13868237	27.94548739	110.0670964	108.7476584
TDRD7	1.610727752	2.82E-10	1.28E-08	58.22078207	36.99672855	113.2208634	118.0343927
ZC3HAV1	1.610384965	1.40E-13	1.29E-11	68.01553282	53.42956444	144.5693066	150.2189527
DDX60L	1.608667692	3.08E-05	0.000361531	164.2391642	51.58416576	240.4438211	283.4233322
ICAM2	1.605505341	0.000164657	0.00151929	16.14253282	6.064331577	32.29520407	21.26803932
FAM72A	1.605342497	3.56E-05	0.000410168	6.034350045	4.39468515	12.42647244	12.83199826
LAP3	1.604780526	2.83E-07	6.17E-06	509.8763289	204.7522565	809.5095252	919.4582938
NLRC5	1.604389772	2.94E-08	7.89E-07	52.42228962	27.15460224	91.9644743	100.5951818
LRRCC1	1.600182905	0.007092751	0.033400875	2.664955785	1.758401317	3.595925048	7.16071015
BISPR	1.599333838	1.22E-07	2.89E-06	20.06043313	11.68840375	34.18746422	42.53536974
PAPD4	1.599313453	1.74E-08	4.94E-07	105.0788697	60.37177854	179.7653455	219.4086677
CDK18	1.598089492	8.49E-09	2.69E-07	17.63133494	9.930881199	34.75514227	31.61814013
LOC101927021	1.597661431	0.009116289	0.040729775	1.176153671	1.494772933	3.280548356	3.119917372
PTMS	1.593774014	3.65E-08	9.61E-07	146.6869709	77.85912796	291.660996	245.9928307

RNF144B	1.593042141	1.30E-09	4.95E-08	15.43731077	13.62167856	33.30440949	36.43873502
ASPHD2	1.585439189	0.002872919	0.016094366	5.094053973	4.39468515	12.23724642	10.35080971
TSNAX-DISC1	1.585372331	1.78E-05	0.000228986	11.20597844	6.415836088	17.91402689	24.31635668
ITM2B	1.585176639	2.81E-16	4.19E-14	550.622492	406.4279697	1076.507433	1207.347056
UBE2F	1.585119481	1.01E-12	7.85E-11	107.5079679	71.35629451	212.6275969	213.8791618
ARHGAP17	1.583850907	3.88E-15	4.75E-13	83.76549204	74.16833059	184.9375233	191.3357915
NAGK	1.582636673	5.43E-07	1.11E-05	481.7458047	208.6188061	868.4218913	772.4301495
TGIF2	1.582574397	3.13E-09	1.10E-07	12.69478056	11.24902312	29.58296451	27.36467404
SPTLC2	1.582084744	4.43E-14	4.57E-12	84.15728207	61.77779658	174.7823938	172.4078675
SENP7	1.580117026	0.001572115	0.009778961	3.291819834	3.867428383	7.191219343	9.854572003
OGFR	1.579131627	2.21E-12	1.52E-10	251.843415	189.286058	573.5446838	471.5683153
RTCB	1.575379621	2.88E-08	7.77E-07	333.2573833	158.7930416	586.3489775	578.5429873
QSER1	1.571105418	1.72E-08	4.88E-07	13.63507663	11.86415601	26.42919759	33.95754647
TBC1D4	1.56908501	5.58E-05	0.00060712	5.720918021	3.428047744	8.011198744	13.75358258
CD209	1.568933155	1.78E-12	1.30E-10	38.39620654	27.3303545	70.8342359	84.50290175
ARHGAP15	1.562333039	1.85E-09	6.85E-08	18.49327301	14.93982048	40.24269672	38.21101255
LMO2	1.560260791	7.18E-17	1.25E-14	97.79157512	86.29523623	220.6381649	210.4763889
CRYZ	1.556081121	2.38E-06	4.08E-05	7.836584184	7.646101877	13.75105455	22.61497025
OPTN	1.554121403	1.57E-06	2.81E-05	100.4557473	46.92673099	172.4486062	171.4862831
DBF4B	1.550661725	0.00037483	0.003035449	4.1537579	10.10663345	14.25565726	18.92863298
MLKL	1.549108593	4.14E-07	8.75E-06	50.69841349	23.20017649	82.56624886	89.53616995
RAB8B	1.548274411	1.27E-08	3.79E-07	62.92226243	44.81770392	107.1025555	144.0514269
B3GNT2	1.547791776	6.59E-10	2.70E-08	51.09020352	66.78673586	112.3378086	162.3413311
CBS	1.544637697	0.003325575	0.018075847	1.881375725	4.043180638	7.065068666	6.593581339
EHD4	1.543977723	1.00E-13	9.54E-12	146.5302549	100.7069212	280.6858871	292.4265021
LOC101927027	1.543287405	0.000548126	0.004156599	12.22463252	5.888579322	18.98630764	23.11120796
NLK	1.540840862	4.15E-07	8.75E-06	11.04926243	10.19450958	21.19394449	28.07358506

BTN3A1	1.539182869	0.000101543	0.001005747	27.03429566	10.54601409	40.43192274	46.57616252
FMNL2	1.538310072	6.35E-09	2.08E-07	31.73577603	30.40601897	58.7237709	85.21181277
BARD1	1.536330161	0.000603666	0.004499746	4.937337961	3.6038	7.821972728	11.98130504
C15orf48	1.536256504	3.70E-11	1.97E-09	73.18716122	71.79567514	178.2515374	155.323112
HACD4	1.53501843	4.14E-07	8.75E-06	22.01938328	13.0944218	42.57648425	38.28190366
TATDN3	1.534533218	1.07E-09	4.21E-08	24.21340745	16.87309529	47.2440593	47.42685573
USP6NL	1.534200521	2.02E-07	4.56E-06	14.65373071	13.00654567	28.5737591	35.2335863
GAS6	1.532498825	0.000311769	0.002599156	32.7544301	9.403624432	54.30849721	42.46447864
TRIM22	1.532359893	1.18E-05	0.000162428	221.9890147	81.90142984	311.2774263	388.6966178
IL10	1.530708294	1.13E-07	2.71E-06	28.52309778	18.36698946	43.58568966	64.58250227
ESR1	1.53062911	0.007892666	0.036308275	1.567943701	1.23114455	2.839020986	3.616155081
PDGFB	1.530565682	0.000153956	0.001435295	6.112708051	18.71849398	26.23997157	30.69655581
GALM	1.528789608	1.35E-17	2.90E-15	171.9182488	138.5815322	331.6507606	381.182161
RAB39A	1.528648533	0.001180867	0.007781321	4.467189924	4.570437405	8.389650775	12.40665165
TMEM123	1.52813375	4.39E-07	9.15E-06	257.4851914	154.1356069	398.0060167	548.1307048
SOS1	1.526071625	4.14E-09	1.43E-07	23.27311137	26.10008871	49.07324411	64.15715566
KCNJ5	1.524889855	0.002548482	0.014610934	3.37017784	2.373534211	5.614335881	7.586056758
IL1R2	1.524145225	5.03E-08	1.29E-06	22.56788932	25.1334513	48.31634005	60.89616499
MYPOP	1.521870466	8.89E-05	0.00089932	8.698522251	5.624950938	18.79708163	13.82447368
RNF149	1.518048728	4.21E-16	6.08E-14	47.48573524	51.14478512	104.6426173	120.0193435
NHLRC3	1.513922483	2.18E-07	4.89E-06	47.40737723	23.02442424	72.09574267	88.18923903
CBWD3	1.513132547	4.68E-07	9.69E-06	10.73583041	8.70061541	23.14927999	20.91358382
MAML2	1.512392599	4.57E-07	9.50E-06	18.57163101	25.30920356	41.44112815	58.27319424
MICB	1.508496011	5.72E-09	1.91E-07	33.45965216	27.15460224	55.38077796	82.23438651
STARD4	1.507936665	1.75E-07	3.97E-06	23.19475337	17.92760883	40.68422409	52.53101503
TMEM60	1.507226148	1.40E-10	6.70E-09	33.14622014	24.60619454	68.62659905	61.74685821
RERE	1.50320136	6.74E-14	6.66E-12	69.81776696	58.61425598	138.9556015	150.5734082

TNFSF13	1.50145712	2.23E-08	6.16E-07	139.4780343	77.33187119	254.8249983	232.5235214
MRPL40	1.500985506	5.39E-07	1.11E-05	65.35136062	30.58177122	112.5270346	103.1472614
GNB4	1.499121178	1.26E-06	2.33E-05	71.30656908	39.80876464	102.1826791	148.3757841
PTBP3	1.499119735	9.73E-11	4.82E-09	72.79537119	69.86240033	136.3695126	184.9555924
PSME1	1.49900475	2.69E-07	5.91E-06	243.6158244	122.0608202	427.7775765	392.7374105
CYSLTR1	1.498834622	0.000254408	0.002178852	8.620164244	3.515923872	11.29111635	16.16388003
LPAR6	1.49307621	3.47E-10	1.52E-08	176.4630132	114.6792255	291.0302426	361.5453259
NREP	1.492955722	8.55E-05	0.000873855	5.799276027	5.185570299	11.29111635	13.32823597
GIMAP2	1.492945091	1.45E-06	2.62E-05	43.72455095	20.73964491	73.48340011	70.75002809
ARHGAP25	1.491911514	1.71E-08	4.86E-07	114.5601884	61.77779658	193.1373173	201.1896546
CSF3R	1.486710225	5.18E-09	1.75E-07	58.06406606	34.36044472	104.0749393	101.8003305
PELI1	1.48497597	3.20E-06	5.25E-05	20.76565518	19.421503	37.0889298	52.60190613
RNF217	1.482381267	9.82E-06	0.000139451	10.10896636	6.415836088	17.85095155	18.92863298
ENTPD5	1.482026003	5.49E-05	0.000598609	14.5753727	7.733978005	20.43704043	29.34962488
PARP9	1.480809366	2.19E-05	0.000271157	282.1679633	99.65240765	400.5921056	447.0399942
TYMP	1.479418903	1.75E-06	3.08E-05	2593.494067	1246.347999	4677.289282	3811.035428
HERPUD2	1.477115583	2.74E-10	1.25E-08	29.14996183	27.50610675	56.38998338	69.19042386
USP25	1.475524617	2.14E-06	3.70E-05	33.14622014	19.86088364	51.97470968	65.50408658
GPBP1	1.474198413	1.24E-13	1.15E-11	55.71332587	49.21151031	108.1748363	123.7765719
SUSD1	1.473196993	2.23E-10	1.03E-08	32.91114612	46.75097873	78.46635186	97.47597331
TRIP4	1.472114278	2.74E-12	1.85E-10	35.41860231	30.40601897	68.12199634	77.20111831
CCNJ	1.470379646	2.74E-05	0.000328522	11.12762044	8.173358643	18.48170494	24.17457448
LMBR1	1.469519683	8.49E-07	1.65E-05	11.20597844	9.315748304	20.94164314	24.31635668
SNN	1.469442667	2.56E-15	3.30E-13	117.6945087	102.3765676	235.2716434	249.1829303
IFI16	1.467628234	2.38E-06	4.08E-05	242.1270222	111.1641804	347.8611226	430.097021
DGKG	1.466937383	0.000643185	0.004725968	7.679868172	3.867428383	11.92186973	13.54090927
BLOC1S3	1.465105918	3.02E-10	1.36E-08	36.35889838	26.80309773	68.68967439	69.97022597

UBXN2A	1.464565028	0.000786991	0.005572655	3.213461828	4.043180638	6.182013927	9.783680901
ACE	1.464077783	2.69E-11	1.49E-09	142.7690706	100.0917883	272.8014698	259.3203578
STK3	1.463304477	0.000389261	0.003124264	8.698522251	5.976455449	13.30952718	18.99952408
LARP7	1.461247104	1.42E-08	4.18E-07	37.76934249	25.7485842	63.51749663	75.78329628
MAX	1.460844371	1.29E-12	9.78E-11	92.93337874	69.24726744	165.7626203	189.4926229
FOXN2	1.460513068	6.88E-05	0.000732977	22.95967935	17.2245998	33.05210813	55.29576799
TMX1	1.458504959	2.14E-07	4.81E-06	34.79173826	25.39707969	53.99312052	77.98092043
DOCK8	1.456397428	2.19E-10	1.02E-08	93.95203282	93.58895483	177.7469347	232.1690659
SRD5A1	1.454161037	1.78E-07	4.05E-06	10.26568237	9.843005071	20.31088975	23.53655457
UBP1	1.453031173	1.96E-10	9.20E-09	48.66110533	39.54513626	82.18779683	110.3072627
RAB12	1.452139535	5.37E-09	1.81E-07	37.37755246	24.60619454	62.94981858	72.09695901
CDC73	1.450787891	1.09E-06	2.07E-05	25.62385156	24.07893777	45.35179914	62.95200693
C6orf62	1.446949653	1.46E-05	0.000193347	407.384057	148.6872869	542.1331652	664.7465665
GPD2	1.445479583	1.42E-07	3.32E-06	48.50438932	30.23026671	74.36645485	96.55438899
IL12RB1	1.44544129	7.24E-05	0.000765541	23.89997542	10.63389022	38.35043657	36.43873502
PARP12	1.444858386	6.59E-07	1.33E-05	260.9329437	117.75489	406.5842628	412.65781
RIMKLB	1.444767755	0.000468812	0.003649098	7.444794154	4.921941916	14.06643124	12.69021606
ACTN2	1.442218113	0.003586412	0.01926145	0.862721647	0.703887783	2.019041586	1.347639837
NR4A2	1.440767894	4.15E-05	0.00047186	9.011954275	15.02769661	24.78923879	27.01021854
ERAP2	1.438093858	1.17E-05	0.00016091	39.33650261	17.66398044	58.78684624	64.15715566
SYNGR2	1.437865243	2.03E-09	7.47E-08	402.7609346	291.2223662	837.0103727	652.9786437
GLRX	1.435145197	9.20E-14	8.85E-12	112.0527322	98.68577024	234.073212	218.3453012
VRK2	1.434431401	4.98E-06	7.76E-05	25.38877754	18.19123721	42.8287856	50.9714108
CXorf21	1.434348567	1.54E-07	3.56E-06	26.95593766	21.61840619	47.81173734	56.71359001
SP140L	1.433948742	6.11E-10	2.51E-08	38.63128056	28.20911577	67.68046897	76.06686069
CALCOCO2	1.433793901	1.39E-08	4.09E-07	83.295344	50.00239546	124.5744243	162.4122222
SGPP1	1.43361121	3.97E-05	0.000453922	11.67612648	8.173358643	19.68013637	23.04031686

PRR5	1.431246391	0.000136493	0.001295878	21.54923524	8.788491538	33.99823821	31.05101131
WDR44	1.431112956	9.19E-06	0.000131598	10.73583041	9.052119921	18.16632824	24.38724779
DEK	1.430016054	1.11E-06	2.09E-05	85.33265216	58.61425598	126.9712872	182.3326217
RHPN1	1.429727539	2.14E-05	0.00026633	14.10522467	13.53380244	34.81821761	24.10368338
LOC648987	1.427759655	2.95E-05	0.000349188	12.61642255	7.382473493	20.0585884	22.82764355
FLT3LG	1.427503231	0.000756187	0.005402242	8.071658202	5.097694172	16.58944478	11.48506733
DLGAP4	1.425008922	5.50E-15	6.51E-13	46.46708116	42.5329246	91.90139896	98.11399322
RNF114	1.421836396	3.27E-12	2.18E-10	128.1944815	92.97382194	239.1823144	231.531046
WASHC4	1.419531381	1.26E-05	0.000171279	55.55660986	36.2058434	70.0142565	126.1868694
EXOC6	1.418207031	1.13E-06	2.13E-05	11.75448449	10.80964248	22.32930058	25.66328761
TNF	1.416472424	0.000238887	0.002075485	36.43725639	109.9339146	167.7810312	141.9246939
CDK17	1.414872588	0.000282853	0.002402499	6.661214093	7.11884511	11.85879439	17.43991985
FCGR1B	1.412393698	3.24E-07	7.00E-06	13.63507663	13.88530695	32.23212873	25.80506981
GMDS	1.411331304	0.000335654	0.0027679	9.168670287	4.043180638	14.38180793	13.61180038
SHOC2	1.411008912	1.24E-08	3.72E-07	31.10891198	25.1334513	56.20075736	62.81022473
ERP44	1.406486043	2.55E-12	1.74E-10	125.0601612	88.84364393	219.2505074	231.6019371
IGSF6	1.397008076	8.77E-08	2.15E-06	76.71327149	85.94373172	164.3118876	176.1650958
FAM177A1	1.396698718	6.34E-07	1.28E-05	17.63133494	14.23681146	33.17825881	33.53219986
RGS2	1.395528387	5.91E-05	0.000637685	69.81776696	42.5329246	111.0763019	124.0601363
USP42	1.390710674	0.000196968	0.001769894	10.81418841	6.152207705	17.2832735	18.14883086
P2RY6	1.388896209	9.11E-09	2.86E-07	226.220347	143.941976	394.0322704	375.7944373
NECTIN2	1.385248877	9.76E-09	3.02E-07	230.4516793	153.8719785	439.3834388	356.5829488
DDX60	1.38119009	0.001931845	0.011563028	42.62753887	12.91866954	46.1087032	69.40309716
TRPC2	1.381139218	0.003301852	0.017980763	1.803017719	2.285658083	4.857431818	3.54526398
PAX8-AS1	1.37891209	4.07E-06	6.48E-05	51.63870956	24.25469002	78.40327652	78.54804924
CREG1	1.378138822	3.40E-10	1.50E-08	887.013412	578.4015517	1445.498163	1583.211676
GOLGA4	1.377977403	2.55E-05	0.000308158	26.64250563	21.70628232	39.67501867	60.61260059

SFT2D2	1.376893926	4.74E-06	7.42E-05	14.4970147	13.35805018	32.29520407	25.0252677
GTF2B	1.372783868	2.02E-12	1.41E-10	42.31410684	38.49062272	84.269283	81.9508221
PAX8	1.372392396	5.29E-06	8.18E-05	49.05289536	25.48495581	81.55704344	71.6716124
CENPN	1.371960853	1.37E-06	2.50E-05	14.73208872	10.28238571	26.1138209	25.3797232
STOML1	1.371770482	8.63E-05	0.000879906	9.403744305	7.206721238	17.03097215	17.15635544
LMO4	1.371537247	2.66E-12	1.80E-10	36.20218237	31.02115186	63.01289392	75.49973188
BTN3A3	1.370700312	9.97E-05	0.000992115	40.59023071	16.43371465	57.14688744	60.18725398
SRGN	1.369000661	1.19E-13	1.11E-11	670.3535253	499.4009128	1123.813937	1279.514198
KIAA1328	1.367203575	0.001939159	0.011592379	1.881375725	1.406896806	3.09132234	3.687046183
LCP2	1.361887404	8.19E-09	2.61E-07	166.0413984	111.7793133	253.7527176	314.8280901
TIPRL	1.357118621	4.50E-08	1.17E-06	31.65741802	21.09114942	49.51477148	58.13141204
RRBP1	1.356357975	4.01E-10	1.73E-08	216.5039543	142.9753386	347.7349719	384.3013695
ZNF277	1.351207589	2.46E-05	0.000299338	24.05669143	15.46707725	37.40430649	42.88982524
MYD88	1.350152537	1.33E-08	3.94E-07	213.604708	130.145424	310.8358989	386.9952313
PTP4A1	1.347990496	7.74E-10	3.09E-08	92.46323071	77.33187119	154.2198334	189.9179695
KLF6	1.347371236	1.26E-08	3.77E-07	269.0038183	392.8071699	659.8948222	678.0032025
GPR160	1.347293447	8.00E-05	0.000829802	8.698522251	6.767340599	12.17417109	19.28308849
SCAMP1-AS1	1.344990362	0.000138828	0.001314585	11.51941047	7.11884511	19.36475968	18.29061307
UTRN	1.344828834	2.54E-06	4.33E-05	36.28054038	30.66964735	56.20075736	79.39874245
GIMAP7	1.343410736	0.002535862	0.014573885	21.15744521	6.32795996	25.60921819	29.98764479
RCSD1	1.343212844	1.63E-07	3.73E-06	63.62748448	39.369384	89.50453609	118.8141948
CBWD6	1.342065923	0.000158255	0.001466785	7.836584184	6.064331577	15.07563666	12.90288936
SCARNA9	1.341436648	0.009673256	0.042794225	11.9895585	4.306809022	18.73400629	14.03714698
FAM126B	1.340965888	0.002823141	0.015858604	7.444794154	6.064331577	11.41726702	15.88031562
ABCD1	1.33879883	2.83E-15	3.59E-13	102.9632035	107.2097546	211.6183914	210.6181711
DUSP4	1.336913216	6.78E-05	0.00072351	2.116449743	3.779552255	4.920507157	6.877145745
CYBA	1.336877672	9.84E-07	1.88E-05	848.382915	542.7238438	1636.237987	1145.600907

C2CD2L	1.334893717	2.56E-07	5.66E-06	71.85507512	42.18142009	120.0960753	108.3223118
ZBED1	1.333781889	7.57E-08	1.87E-06	94.73561288	58.08699921	148.732279	157.5916273
DRAP1	1.332954018	4.44E-05	0.000498405	316.0969799	129.0909104	488.0145248	401.3152338
SRGAP2	1.331112008	1.68E-10	7.94E-09	156.0899316	108.6157727	249.7158959	280.0914505
TRIM26	1.330830651	1.04E-08	3.20E-07	73.73566726	50.09027159	117.3207604	130.5112265
LINC01410	1.330322894	0.002766509	0.015576857	6.347782069	3.955304511	10.40806161	10.20902751
WIPF1	1.3297928	2.65E-09	9.57E-08	242.9106023	179.1803033	372.7758813	472.1354441
ODF3B	1.329780869	2.00E-05	0.000250915	53.67601772	29.70300995	96.50589867	69.54487936
CEP57L1	1.329661277	0.003362022	0.018222435	5.485844003	2.988667105	7.380445359	9.641898698
MED13	1.328567588	1.59E-07	3.66E-06	29.22831983	31.4605325	49.7039975	71.81339461
ACSS1	1.327171256	7.58E-09	2.45E-07	18.25819899	16.34583853	36.64740243	32.25616004
HES6	1.327082365	0.005771413	0.028396613	2.97838781	2.637162594	7.75889739	3.332590676
CTNNBL1	1.326308979	1.12E-11	6.74E-10	96.06769899	75.134968	162.4827027	179.0007399
C4orf33	1.325992301	0.000292723	0.002470341	17.47461893	7.733978005	23.71695803	26.65576303
PHACTR4	1.325307113	2.13E-08	5.94E-07	36.98576243	28.73637254	57.58841481	73.65656324
EMP1	1.325002508	5.64E-09	1.88E-07	266.1045721	397.8161092	553.7390275	771.7212385
PLEKHO1	1.321593461	8.29E-09	2.64E-07	577.2642141	393.9495595	1046.357421	878.7668016
TLE4	1.319258572	2.59E-11	1.45E-09	53.83273373	49.56301482	93.09983039	112.2922135
SAMSN1	1.314076966	1.18E-06	2.21E-05	112.8363123	68.63213455	156.1120936	203.3872788
HLA-B	1.311591428	8.31E-08	2.05E-06	4467.033992	3198.252549	8503.25009	6572.810955
PDE7A	1.311050277	1.24E-07	2.92E-06	24.60519748	32.86655055	50.90242893	62.52666033
TNK2	1.310057681	2.82E-06	4.73E-05	105.4706597	57.47186632	168.4117846	152.3456858
DMXL1	1.308598506	1.06E-05	0.00014929	33.77308418	30.84539961	52.41623705	75.21616747
C3orf58	1.308287937	0.000134361	0.001278161	10.50075639	16.17008627	21.88777322	30.69655581
CLEC2B	1.308062434	5.00E-08	1.28E-06	51.40363554	41.47841107	95.11824122	87.48032801
FAM72B	1.307903687	0.00405543	0.021366889	5.094053973	3.955304511	9.02040416	8.791205482
OGFRL1	1.307814511	5.45E-08	1.39E-06	75.92969143	53.42956444	118.2038151	136.6787524

TNFSF8	1.307031493	1.43E-08	4.19E-07	50.93348751	36.99672855	75.50181095	97.90131991
STAC3	1.306715939	2.50E-05	0.000303489	81.33639385	38.13911821	128.4850953	105.9829055
MYL12A	1.305140491	2.77E-10	1.26E-08	665.7304029	449.6630245	1125.580046	1062.800101
CAST	1.304758412	8.81E-09	2.79E-07	136.6571461	116.7882526	215.9075145	282.8562034
ADAP2	1.304370813	1.64E-14	1.85E-12	290.708986	244.6480185	526.6166319	523.7441659
CASP4	1.304008316	3.45E-11	1.87E-09	101.1609694	77.41974732	172.7009076	177.7955912
EREG	1.303202704	0.003616544	0.019401615	13.00821258	43.14805749	56.32690804	53.59438155
SCARB2	1.302328056	8.98E-13	7.12E-11	436.3765192	343.0692816	716.8518529	812.2000574
PATL1	1.3013529	2.80E-06	4.70E-05	96.85127905	63.44744301	136.6848893	178.0791556
SLC25A28	1.294635359	8.65E-07	1.67E-05	59.08272013	36.55734791	96.0643713	90.31597207
CLDN23	1.293842257	1.50E-09	5.69E-08	60.57152225	44.72982779	102.6242065	102.5801326
UNC93B1	1.292453046	2.24E-09	8.17E-08	555.0105404	392.3677892	979.6237131	861.540264
FYB	1.29123468	5.10E-05	0.000563875	79.84759173	53.25381219	106.0302748	153.7635078
CCNK	1.289866168	1.23E-07	2.92E-06	46.23200714	34.09681633	68.8789004	87.62211021
USP15	1.287331032	1.45E-06	2.61E-05	88.93712044	53.69319283	132.7742183	144.122318
MFHAS1	1.287157474	0.001152713	0.007623648	6.347782069	7.90973026	12.3633971	15.31318681
CKAP4	1.286731384	2.04E-12	1.42E-10	69.66105095	73.5531977	133.3418964	144.4058824
ATG4C	1.286145988	5.41E-06	8.33E-05	24.68355548	15.90645789	37.27815581	41.54289432
LIPA	1.285416831	8.19E-06	0.000119056	750.9055555	346.5843267	954.8981804	1174.666259
HLA-E	1.282292793	2.80E-08	7.59E-07	2547.497918	1546.532851	4114.783414	3789.059186
CD164	1.282002033	6.57E-09	2.15E-07	246.4367126	176.1046388	360.4761903	457.8154416
CNTLN	1.281503869	0.001777481	0.010804897	9.717176329	5.449198683	12.3633971	17.08546434
KARS	1.281218528	1.11E-09	4.34E-08	158.8324618	107.8248875	246.8144303	268.607092
TTC21A	1.280136632	0.009434715	0.041854405	6.817930106	3.428047744	10.09268491	9.7127898
FCGR1A	1.279826968	1.16E-09	4.51E-08	45.29171107	42.97230524	85.59386511	84.64468396
HOXB2	1.279356926	0.007398657	0.03451989	2.429881767	3.164419361	5.172808511	5.60110592
SLC25A30	1.277728895	8.50E-06	0.000122729	18.49327301	19.06999849	30.33986858	42.25180533

43891	1.277243386	4.01E-05	0.0004574	119.8101748	67.92912552	160.9058193	201.473219
ACOT9	1.276599279	5.64E-09	1.88E-07	127.0974694	88.31638716	208.9061519	205.7266851
LATS1	1.276571917	5.34E-05	0.000584414	14.73208872	14.76406823	23.71695803	33.24863546
GOPC	1.275990325	3.39E-05	0.000392845	14.88880473	13.70955469	25.48306751	29.63318929
ADPRH	1.274824555	1.10E-06	2.09E-05	43.80290896	26.89097386	61.56216114	74.71992976
SIGLEC14	1.274530147	6.28E-06	9.48E-05	37.69098449	23.72743326	54.18234653	64.15715566
TMEM106A	1.273347485	1.54E-08	4.44E-07	120.3586809	78.38638473	177.1792566	205.1595563
CNOT4	1.27133793	0.000188065	0.001701712	14.18358267	15.99433401	24.9784648	33.03596215
CARD16	1.266545902	6.93E-06	0.000103486	71.6200011	38.66637498	115.4285002	95.06567586
NEDD1	1.265852584	0.001504496	0.009445291	8.933596269	6.240083833	12.04802041	17.08546434
ARHGAP12	1.265010865	5.34E-05	0.000584414	12.45970654	12.4792889	21.88777322	25.80506981
GNG5	1.264946364	2.67E-09	9.62E-08	175.3660011	123.5547144	279.9920584	291.0086801
RAP1GAP2	1.260403104	0.00015714	0.001459682	11.04926243	8.085482516	16.27406809	20.27556391
NRSN2	1.26032281	0.001324487	0.008570994	3.213461828	2.988667105	5.046657834	6.806254643
DNAJC15	1.260063156	5.43E-06	8.36E-05	43.33276092	24.34256615	62.94981858	66.00032429
DECR1	1.25923911	3.38E-06	5.50E-05	80.31773977	43.85106652	112.968562	123.4930075
ANXA4	1.256709737	5.24E-11	2.71E-09	195.8957987	146.9297644	303.1407076	348.6431455
DIAPH2	1.255889214	0.00024573	0.002118353	12.38134853	7.558225749	16.52636944	21.48071263
PLEK	1.255150877	8.53E-10	3.39E-08	327.3021748	230.7635903	495.7097161	563.9394204
STAT2	1.254816434	2.41E-05	0.000294001	360.9961174	158.353661	463.0997661	522.7516905
FANCA	1.253526984	0.00024807	0.002134704	14.88880473	8.173358643	23.14927999	20.55912831
MEF2A	1.253077047	3.57E-06	5.79E-05	70.366273	43.85106652	96.82127536	120.0193435
MIA3	1.253062368	1.17E-06	2.19E-05	42.94097089	32.51504603	65.22053077	77.98092043
ATP6V1G1	1.252321166	7.58E-13	6.11E-11	101.6311174	98.2463896	178.5038387	200.1971792
ZNF766	1.251704844	0.000840407	0.005847598	20.92237119	11.24902312	30.71832061	30.2003181
TMSB10	1.251087562	2.94E-05	0.000347689	2524.303948	1267.701898	4052.086527	3099.076097
SLC25A32	1.250918832	7.14E-05	0.000756445	7.053004124	6.503712216	10.84958898	14.8878402

RASA2	1.249533224	0.004679673	0.023922197	7.288078142	3.691676127	8.137349421	12.76110716
RBMS1	1.246612691	7.69E-07	1.52E-05	43.56783494	38.93000336	64.02209934	92.0882496
OCEL1	1.245537496	0.000171825	0.001577358	9.560460317	10.54601409	20.75241712	17.01457324
BMPR2	1.244981435	9.52E-05	0.000953346	31.89249204	18.8063701	39.73809401	56.1464612
TCF7L2	1.241627068	0.000425438	0.00336595	5.720918021	7.206721238	12.86799981	11.34328513
CIR1	1.241155505	1.39E-05	0.000187291	61.90360835	39.45726013	89.50453609	101.1623106
ARID5A	1.238225553	1.63E-10	7.72E-09	78.75057965	72.41080804	152.1383472	130.7238999
PPP2R5C	1.238136112	6.30E-09	2.07E-07	70.05284098	54.83558249	114.4192948	119.8066702
STX17	1.237792383	9.57E-06	0.000136251	17.63133494	15.81858176	28.63683444	34.17021978
AGPAT3	1.236926454	2.15E-11	1.22E-09	157.8921658	124.1698473	253.1219642	275.55442
LMF2	1.236757422	5.90E-09	1.96E-07	95.12740291	77.68337571	171.8178528	151.3532104
NUP205	1.236038116	8.22E-07	1.60E-05	38.55292255	24.86982292	57.20996278	61.67596711
COG5	1.235105726	1.98E-05	0.000249783	28.05294974	19.59725525	36.83662844	52.67279724
DENND1A	1.234550884	1.51E-07	3.50E-06	42.94097089	31.54840863	61.62523647	78.05181153
FKBP1B	1.233913803	8.12E-05	0.000839958	13.63507663	10.98539473	24.28463608	21.69338593
UBA7	1.233800486	4.33E-05	0.000489347	147.862341	66.96248812	208.0861725	193.1080691
SLC35A3	1.232643398	0.002735848	0.015452453	4.1537579	4.39468515	8.074274082	7.869621164
SMIM14	1.230776521	1.10E-07	2.64E-06	18.88506304	21.0032733	36.83662844	37.50210154
NUDCD1	1.230613343	0.000346126	0.002846126	8.385090226	6.591588344	12.42647244	15.59675121
NUP160	1.230034239	5.98E-05	0.000644142	15.51566878	14.50043984	22.64467728	33.53219986
BAZ1A	1.229936661	6.32E-07	1.28E-05	59.47451016	54.5719541	91.1444949	122.0042944
C2CD5	1.229185646	1.71E-05	0.000221572	21.15744521	15.99433401	30.84447128	38.56546806
ZBTB41	1.228583908	0.006284291	0.030457105	4.702263942	2.725038722	6.245089266	7.65694786
SLC35A5	1.227084234	8.79E-06	0.000126375	21.94102527	14.58831597	30.52909459	37.64388374
RIC1	1.2268398	2.44E-05	0.000297532	24.05669143	23.63955713	38.7288886	50.19160869
MSR1	1.22314749	1.20E-08	3.64E-07	558.0665026	514.2519784	800.9943545	1195.437351
CRTAM	1.222853561	0.003554627	0.019103941	7.91494219	8.349110899	16.33714343	13.75358258

NXT2	1.22203856	0.002144502	0.012626402	6.7395721	6.767340599	9.903458899	15.24229571
KCTD12	1.22144893	5.70E-06	8.72E-05	206.8659195	187.7921638	276.9644421	457.6736594
TNFSF12	1.218734364	3.54E-06	5.75E-05	46.38872316	29.17575318	72.09574267	67.63081963
PTGER2	1.217757565	3.05E-06	5.05E-05	28.05294974	33.6574357	61.24678444	52.53101503
NOD1	1.216530825	3.94E-05	0.000450401	21.86266727	14.85194435	36.26895039	31.47635792
RALB	1.216193554	2.33E-11	1.31E-09	93.40352678	84.09833303	146.3984914	181.9781661
SDHAF4	1.214869549	0.004450021	0.023017294	3.526893852	2.637162594	5.109733172	6.310016934
TMEM9B	1.214320167	2.08E-08	5.82E-07	68.87747089	50.4417761	108.9948157	111.0870648
CFD	1.213541229	0.000751401	0.005373375	71.93343312	31.63628476	110.2563225	80.03676237
PSMB10	1.211918607	8.04E-05	0.000833791	216.4255963	126.6303789	356.6285947	273.0732314
SP100	1.210895579	7.54E-08	1.87E-06	151.2317352	101.0584257	233.3163079	230.6803528
ATF3	1.208254575	2.46E-05	0.000299338	69.19090291	109.9339146	160.5273673	168.2961836
MIER3	1.206859286	0.004169606	0.021849967	4.780621949	5.449198683	7.75889739	11.05972073
AZI2	1.20600197	9.56E-07	1.84E-05	38.23949053	27.94548739	53.48851781	68.19794844
TTC39B	1.205431524	0.001820696	0.01102579	4.858979955	7.821854132	9.651157545	13.61180038
DNAJA1	1.204198289	4.14E-08	1.08E-06	288.9067519	183.2226052	414.9102075	450.0883116
SUB1	1.203652512	8.85E-11	4.42E-09	69.50433494	59.05363662	118.0145891	117.2545906
ARHGEF3	1.2034034	0.000167403	0.001542649	12.69478056	9.930881199	16.58944478	25.0252677
ARPC5	1.20063303	3.38E-12	2.22E-10	361.7796975	330.0636146	585.7812995	679.2792423
BLNK	1.200470212	0.000234537	0.002045075	33.53801017	16.52159078	44.34259372	47.21418243
C1GALT1C1	1.198379305	6.29E-06	9.49E-05	39.10142859	22.84867198	56.01153135	57.06804552
SLC37A1	1.198368948	1.44E-05	0.00019147	16.37760684	14.76406823	28.69990977	28.07358506
LPCAT2	1.196869408	5.25E-05	0.000578048	28.8365298	33.74531182	46.86560726	67.48903742
UBE2Z	1.196690891	9.35E-13	7.33E-11	176.5413712	172.9410982	306.6729266	330.140568
ZKSCAN4	1.196666513	0.000727151	0.00524676	34.32159023	14.23681146	44.15336771	44.37853837
C15orf57	1.19497942	7.49E-07	1.48E-05	30.87383796	24.25469002	47.62251133	52.81457944
KPNA1	1.194969295	1.57E-08	4.50E-07	46.38872316	44.02681877	70.45578387	94.49854705

C5orf22	1.194803102	1.11E-05	0.000154423	19.90371711	14.14893533	28.5737591	33.53219986
SPATA13	1.19291793	0.001068309	0.007163927	26.72086364	14.6761921	32.16905339	43.31517185
PARP14	1.19221074	0.000708143	0.005135283	357.3916491	121.4456873	395.4199279	475.4673259
SCO1	1.191983145	2.82E-06	4.73E-05	20.29550714	15.73070563	30.59216993	34.95002189
PARP8	1.191915075	3.68E-05	0.000422769	24.52683947	20.03663589	37.78275852	43.24428075
PTPA	1.191782524	1.44E-05	0.00019183	244.6344784	143.7662238	357.8270261	346.870868
IMPA1	1.191451503	0.000414096	0.003297914	9.482102311	9.139996049	14.06643124	19.8502173
CASP10	1.190445183	1.09E-06	2.07E-05	46.31036515	30.75752348	69.32042777	70.53735478
SGF29	1.189137766	2.39E-05	0.000291979	27.34772769	18.8063701	45.85640185	37.64388374
CCL7	1.18766742	6.87E-06	0.00010271	123.1012111	153.9598546	254.951149	246.1346129
SMAD1	1.185632065	0.008943583	0.040119627	4.232115906	3.515923872	5.235883849	8.862096583
TMEM50A	1.184628596	2.85E-09	1.02E-07	240.3247881	174.171364	360.1608136	388.9801822
IQSEC2	1.184163345	7.88E-06	0.000115031	28.20966575	19.33362687	43.08108695	42.81893414
FNDC3A	1.181893122	0.000156924	0.001459126	20.06043313	27.68185901	36.01664904	50.26249979
MCUB	1.18003601	3.06E-09	1.08E-07	60.49316424	51.32053738	96.50589867	104.9904301
TGFB1	1.177214597	1.11E-08	3.38E-07	485.428631	332.0847656	742.4604403	725.925587
RNASEK-C17orf49	1.176665756	0.003656258	0.019571015	14.88880473	17.48822819	38.98118995	18.71595967
FYTTD1	1.176550254	1.22E-05	0.000166982	41.7656008	29.35150543	55.12847661	73.01854333
APOBEC3C	1.176169613	0.000875263	0.006052187	24.76191349	10.37026184	29.51988917	33.74487317
GGPS1	1.175136991	1.08E-05	0.000151782	16.29924884	11.60052763	23.40158134	26.79754523
MS4A6A	1.17256008	4.01E-05	0.0004574	84.47071409	42.5329246	115.7438769	111.7250847
ICE2	1.170602475	0.000108586	0.001065986	8.855238263	10.37026184	17.66172554	16.66011773
RAB43	1.170376432	4.55E-08	1.18E-06	36.12382436	39.89664077	64.08517468	71.95517681
PSMB8	1.170184319	0.00032585	0.002700946	244.9479105	109.1430294	345.3381091	286.6134318
GNGT2	1.169412381	0.002915421	0.016281905	9.482102311	7.470349621	16.90482147	13.32823597
CMTR1	1.16807482	1.19E-06	2.23E-05	88.54533041	55.187087	123.6913696	133.2050884
CCDC134	1.16790794	0.003353766	0.018194811	6.034350045	5.449198683	11.98494507	8.436749975

CYTH1	1.16779652	3.12E-13	2.71E-11	146.8436869	148.9509153	269.3954015	258.2569912
PLAA	1.166363164	1.34E-06	2.47E-05	42.15739083	29.26362931	60.17450369	67.41814632
CREM	1.166244889	2.24E-05	0.000276249	30.56040594	18.89424623	48.31634005	39.77061678
DCP1A	1.165833087	7.28E-05	0.000769426	17.1611869	19.86088364	33.05210813	32.89417995
RPS6KC1	1.164884445	1.49E-05	0.000197029	30.40368992	25.92433645	42.8287856	57.84784764
MBNL1	1.164492963	9.84E-07	1.88E-05	98.41843917	83.48320014	137.378718	187.5785632
SCPEP1	1.164339686	7.38E-07	1.46E-05	162.6720041	105.8037366	232.7486298	245.6383752
REEP3	1.162306596	1.17E-06	2.20E-05	29.38503585	21.26690168	42.07188154	48.20665785
RUFY3	1.158167915	9.24E-06	0.000131925	16.29924884	13.18229793	24.66308811	27.71912955
NSL1	1.15800051	0.000130404	0.001247086	29.85518388	15.6428295	36.14279972	44.80388498
TMEM179B	1.157845413	1.44E-06	2.60E-05	79.22072769	55.27496313	125.7097804	112.5048868
C5orf15	1.157155223	1.23E-07	2.91E-06	30.95219597	33.13017893	53.93004518	59.69101627
UBE2D1	1.156950469	0.000116418	0.001129053	26.2507156	16.96097142	35.00744362	41.75556762
FHL3	1.156364378	3.00E-06	4.99E-05	34.08651621	26.45159322	53.04699044	54.23240147
EMB	1.153513111	2.98E-05	0.000351323	17.55297693	15.73070563	24.60001277	34.45378418
ACTR10	1.152871936	1.92E-09	7.10E-08	34.87009627	38.57849885	66.10358551	63.51913575
MIIP	1.151112147	7.65E-05	0.000800663	81.57146787	59.75664564	134.2880264	114.7025109
CCR1	1.151051633	3.84E-09	1.33E-07	399.7833304	369.7836244	570.6432182	791.3580736
PIK3AP1	1.150338843	3.46E-08	9.16E-07	257.6419074	198.249423	346.5365405	459.6586102
RPF1	1.149471518	1.74E-07	3.96E-06	22.88132134	20.65176879	39.67501867	37.00586383
TRAM1	1.148760106	2.48E-06	4.23E-05	98.18336515	87.87700653	141.1632383	187.5076721
PRRG4	1.148149714	0.008924414	0.040095958	4.388831918	4.921941916	6.938917989	9.500116496
UVRAG	1.147539282	5.21E-07	1.08E-05	39.57157663	29.70300995	57.9037915	64.22804676
RB1	1.146698255	0.000191743	0.001729295	100.6124633	61.86567271	108.7425143	178.5045022
ZBTB33	1.145017725	0.000439682	0.003454031	12.77313856	9.227872177	16.02176673	22.82764355
LGALS9	1.143032781	8.35E-06	0.000120836	1163.068667	623.1305007	1585.966942	1546.986323
CCDC50	1.140787758	1.14E-07	2.71E-06	22.09774128	22.5850436	39.86424469	38.35279476

UBE2A	1.140581818	1.31E-10	6.33E-09	78.90729566	70.56540936	121.9883354	140.2233074
NEK3	1.139164582	0.00017933	0.001633783	11.67612648	10.63389022	21.44624585	17.51081095
ST8SIA4	1.136676861	5.44E-07	1.11E-05	75.38118539	54.92345861	107.1025555	120.8700368
ARHGAP5	1.135749861	0.001717219	0.010500502	9.482102311	7.646101877	12.04802041	18.00704866
VAV2	1.134074971	0.001147339	0.007605534	9.090312281	6.064331577	12.74184913	13.75358258
TLK2	1.132860456	2.80E-07	6.12E-06	33.14622014	30.84539961	49.3886208	62.38487812
HEIH	1.13227722	6.93E-07	1.38E-05	80.08266575	57.55974245	125.2051777	114.3480554
SQOR	1.132010171	2.74E-08	7.47E-07	347.6752564	281.468116	563.7680063	530.9750582
FCGR1CP	1.129983612	8.95E-06	0.000128408	15.35895276	13.70955469	25.86151954	24.67081219
PILRA	1.129410681	3.52E-09	1.23E-07	335.7648395	266.0897936	550.7114113	494.1825766
FECH	1.127490744	1.87E-05	0.000238267	27.73951772	19.24575074	35.70127235	46.15081591
B2M	1.127275744	1.20E-07	2.86E-06	6559.976333	4281.237947	9569.475612	9236.473199
MRPL1	1.127236385	0.000427656	0.003376126	10.6574724	6.855216727	14.12950658	16.37655333
ZNF800	1.125074906	0.006644394	0.031711674	4.858979955	6.32795996	8.831178144	10.56348302
ZNF124	1.124917477	0.000743835	0.005332484	8.541806238	6.415836088	11.35419168	14.6751669
SGMS1	1.123233775	2.22E-06	3.82E-05	39.33650261	30.58177122	55.75922999	65.50408658
ERBIN	1.122505084	0.000169441	0.001557457	36.43725639	44.46619941	53.99312052	86.55874369
ADAR	1.120054797	2.81E-05	0.000335412	414.5929935	211.2550899	500.8188185	581.5204135
ST6GAL1	1.119869494	0.000439923	0.003454041	16.45596485	8.70061541	19.04938298	24.59992109
NADK	1.118862789	1.16E-08	3.52E-07	323.4626325	258.796075	525.4812759	478.1611877
RABAC1	1.118086339	0.000510998	0.003926629	138.9295283	89.28302457	232.0548011	160.0728158
GRIN2D	1.117168444	0.008841334	0.039759829	11.51941047	4.658313533	14.25565726	13.68269148
RAD9A	1.11626847	0.000234263	0.00204392	21.54923524	15.73070563	35.25974498	28.85338717
COQ10B	1.115787005	1.11E-06	2.09E-05	26.1723576	25.30920356	40.11654604	48.70289556
RILP	1.115475276	8.91E-05	0.000900732	42.78425488	31.63628476	73.10494808	54.65774807
FAM208B	1.115263612	0.003244383	0.017775025	9.168670287	9.227872177	13.87720522	17.86526646
ZC3H12C	1.114404101	0.008241714	0.037638028	8.071658202	15.20344886	14.94948598	25.237941

PSMA2	1.114008492	1.69E-06	3.00E-05	120.8288289	84.88921818	177.6838593	176.0942047
LIN7C	1.113863476	0.000616112	0.004566573	14.18358267	9.667252815	15.9586914	25.3088321
IGF1	1.113705252	0.005516009	0.027363878	28.44473977	11.68840375	22.64467728	46.93061802
HTATSF1P2	1.113156604	0.005921352	0.029035348	3.918683882	5.53707481	8.263500098	7.940512265
PLEKHF2	1.111652881	6.52E-05	0.000699668	18.414915	19.24575074	29.45681384	35.3044774
BPGM	1.111473676	3.29E-05	0.000383064	14.73208872	16.17008627	23.65388269	29.49140708
ABI1	1.111362672	5.94E-09	1.97E-07	98.65351318	101.4099302	158.5720318	185.3100479
ZNF107	1.110002573	0.00857342	0.038833343	12.69478056	6.943092855	14.06643124	19.7793262
COA6	1.109424476	7.16E-05	0.000758134	36.43725639	21.17902555	51.28088096	47.49774683
LY96	1.10857344	1.14E-05	0.000157746	53.20586968	36.99672855	78.27712584	76.27953399
C3AR1	1.107803479	1.99E-12	1.41E-10	290.94406	299.6584744	495.2681887	516.2297091
RBM34	1.107161629	0.000203377	0.00181505	36.51561439	18.98212236	46.36100456	48.77378666
CMC2	1.105372718	2.40E-05	0.000293769	31.10891198	22.05778683	45.16257312	45.7963604
ARL8A	1.104601259	2.43E-10	1.12E-08	126.7840374	121.5335635	197.931043	226.8522333
CISD1	1.10370637	0.00016118	0.001490063	12.69478056	8.436987027	16.71559546	19.49576179
CCDC85B	1.103423024	0.006276158	0.030433883	59.47451016	25.22132743	91.39679625	52.46012393
RAB2B	1.103131478	0.000148462	0.001390439	16.37760684	11.24902312	20.68934178	26.65576303
STYX	1.10271473	0.000420079	0.003330855	20.29550714	13.35805018	23.40158134	34.31200198
ANKIB1	1.102105058	0.000457898	0.003573781	19.35521107	14.0610592	23.78003337	33.46130876
ZCCHC10	1.101535725	0.002616275	0.014905432	13.40000261	7.90973026	17.2832735	19.14130628
PSMF1	1.100754831	1.00E-10	4.96E-09	123.0228531	109.3187817	195.8495568	200.1262881
NOD2	1.099211071	8.42E-07	1.64E-05	30.16861591	29.43938156	49.26247013	52.24745063
N4BP1	1.09890076	1.28E-07	3.00E-06	97.32142708	87.1739975	141.478615	173.1167785
TINF2	1.097744596	6.81E-09	2.22E-07	81.25803584	67.66549714	124.1328969	129.2351867
GNA13	1.097247236	5.07E-06	7.88E-05	86.50802225	84.97709431	122.6821641	169.7140056
YEATS2	1.096090786	3.72E-07	7.95E-06	59.31779415	53.51744057	90.76604286	101.0914195
RPAP3	1.095586624	0.001110898	0.007401368	14.4970147	8.436987027	18.35555426	20.70091051

NOC3L	1.094727171	0.003985869	0.021038797	4.702263942	4.746189661	7.001993328	9.074769887
LOC100419583	1.09416036	0.000372357	0.003018029	70.67970503	32.60292216	86.16154315	89.18171444
TMEM219	1.093227616	0.000115352	0.001120223	204.4368213	127.8606447	310.7728236	251.3096633
IL10RA	1.091315012	3.80E-11	2.01E-09	352.0633047	307.0400692	544.7823294	571.382986
BTK	1.089913038	2.80E-08	7.59E-07	99.12366122	77.33187119	137.0633413	161.9159845
ETNK1	1.085764394	8.20E-06	0.000119056	39.57157663	37.61186144	56.70536007	73.79834545
SDS	1.085630276	1.08E-05	0.000151782	200.675637	121.5335635	276.5859901	266.5512501
MCL1	1.08560561	2.41E-10	1.11E-08	324.0894966	297.8130758	472.8133682	577.7631851
HIVEP3	1.082579048	0.000114715	0.001114783	15.59402678	12.21566052	22.96005397	23.89101008
RCN1	1.082432292	1.48E-06	2.67E-05	33.06786213	24.95769905	45.79332651	52.03477732
POMP	1.081724835	1.35E-06	2.47E-05	121.2206189	90.952671	175.6654485	181.2692551
RNF213	1.079400234	2.30E-05	0.000282128	579.0664482	315.1246729	679.7635538	824.2515446
SSR1	1.078353361	1.65E-08	4.71E-07	115.5788425	88.66789168	160.2119906	182.9706416
PLCL2	1.078101565	0.00081379	0.005703647	9.403744305	8.788491538	15.138712	15.38407791
CD37	1.077660738	4.36E-08	1.13E-06	235.7800238	186.561898	383.3725382	323.6185867
SERPINB1	1.077610688	2.94E-09	1.05E-07	153.5041174	125.1364847	228.1441301	239.3290672
LENG1	1.076727313	0.000640085	0.00470559	16.69103887	14.93982048	28.19530707	24.74170329
MZT1	1.076667516	0.001399727	0.008941772	6.112708051	4.39468515	8.515801452	9.145660989
IFRD1	1.076433393	1.83E-05	0.00023452	22.2544573	22.14566296	40.68422409	33.53219986
CITED2	1.076386627	3.52E-07	7.57E-06	52.03049959	69.24726744	97.51510409	105.6993411
SETX	1.074497096	1.25E-06	2.33E-05	82.27668992	67.92912552	116.9423083	134.7646926
GIMAP8	1.073768651	0.005233618	0.026224426	51.63870956	20.65176879	52.66853841	68.62329505
KDM6A	1.073015183	0.002506404	0.014433328	10.34404038	17.31247593	21.88777322	24.31635668
RNASEH2B	1.071109985	4.36E-06	6.85E-05	26.1723576	21.09114942	38.09813521	40.90487441
TREX1	1.070494657	0.003483591	0.0187651	144.7280207	55.62646764	190.4250777	142.9880604
CTSO	1.069846697	0.000361903	0.002947302	21.0790872	17.04884755	28.32145774	35.4462596
NUDT3	1.06878605	2.86E-05	0.000339344	45.21335307	30.93327573	62.57136655	64.44072006

ZNF518B	1.06712726	0.002039448	0.012091732	8.228374214	7.821854132	12.23724642	14.53338469
RRAGC	1.067014351	2.33E-09	8.49E-08	81.9632579	78.03488022	121.6729587	145.1147934
ANKMY2	1.066797948	0.000560933	0.004226001	10.97090443	7.733978005	13.68797921	17.58170205
TBK1	1.065366145	0.000124037	0.00119504	36.04546636	30.58177122	46.99175794	64.15715566
METTL14	1.065338658	0.001000139	0.006792006	20.06043313	11.16114699	25.35691683	26.65576303
TMEM19	1.063763612	6.18E-06	9.35E-05	23.42982739	23.99106164	34.50284092	44.44942948
OTUD4	1.063671445	0.000283037	0.002402653	18.88506304	20.30026427	30.40294391	34.73734859
DIP2B	1.062099867	0.000382517	0.003085577	59.00436213	30.31814284	63.76979798	84.92824836
RGL1	1.062071289	1.62E-09	6.10E-08	328.1641129	299.3069699	461.0813552	582.0875423
PCMT1	1.061010114	5.59E-08	1.42E-06	106.8811038	84.53771367	157.121299	160.2854891
H1F0	1.060300802	0.000168709	0.001551954	320.7984603	152.9053411	401.601311	382.8835475
CCNYL1	1.060297626	3.32E-06	5.43E-05	38.70963856	34.71194923	50.77627825	71.3171569
DNASE2	1.056568388	2.54E-07	5.62E-06	88.78040442	83.0438195	152.2644979	131.2201376
OSCAR	1.054612092	0.000103876	0.001026034	64.64613856	113.7125881	158.5089564	135.1900392
PNRC2	1.053110149	1.15E-06	2.17E-05	101.2393274	86.2073601	144.0647039	165.3896484
NAPRT	1.049722394	1.77E-05	0.000228105	45.21335307	51.49628963	90.19836482	68.33973064
SLC25A24	1.049544118	0.001495526	0.009403895	38.39620654	38.93000336	49.32554547	78.40626704
GCA	1.048791002	0.001215731	0.007967403	38.39620654	19.50937913	38.98118995	56.64269891
MRPL42	1.048717798	0.000357788	0.00292204	15.12387875	9.930881199	18.04017757	23.32388126
AES	1.048637661	1.14E-05	0.00015765	353.7871809	230.6757141	529.1396455	429.38811
FKBP5	1.048141712	0.000435765	0.003426986	28.36638177	21.79415845	35.1335943	47.56863794
SUCO	1.04798785	0.006074021	0.029632925	7.366436148	8.085482516	11.79571905	13.61180038
SGTB	1.047566114	0.000693798	0.005043928	76.32148146	49.73876708	84.90003638	122.9258787
PTGIR	1.047201487	0.00102816	0.006946334	29.85518388	16.87309529	39.86424469	36.86408163
TOX4	1.046338137	1.59E-08	4.56E-07	73.26551923	62.4808056	106.9133295	116.0494419
AGPAT5	1.045242537	0.000292199	0.002467367	15.75074279	11.42477537	20.31088975	24.38724779
RHOB	1.044932141	2.01E-06	3.50E-05	96.77292104	73.11381706	138.0725467	140.5068718

FEM1C	1.044590027	0.000581048	0.004349119	14.88880473	16.69734304	23.21235532	28.64071387
EBAG9	1.043694923	0.002062582	0.012198821	16.14253282	10.19450958	20.75241712	22.47318805
MSMO1	1.043615764	7.31E-06	0.000108122	37.53426847	24.69407066	47.30713463	54.87042138
PECAM1	1.043002573	7.11E-06	0.000105546	149.5078591	97.54338058	199.3817758	205.2304474
TRAF5	1.042249323	0.001100883	0.007344818	9.795534335	11.16114699	16.02176673	18.29061307
FNIP2	1.042091163	0.00069253	0.005037249	43.33276092	72.32293191	71.6542153	118.3888482
USPL1	1.04177813	0.000982231	0.006697811	19.12013705	15.20344886	25.10461548	31.19279352
ITGAL	1.040671149	0.000491033	0.003795447	60.72823826	33.0423028	88.74763203	64.08626456
CYB561	1.040525007	0.000168735	0.001551954	20.53058116	19.50937913	34.37669024	30.98012021
NOC4L	1.038386406	8.34E-05	0.000858466	20.45222316	19.421503	36.45817641	28.42804056
ESCO1	1.038091287	0.002526159	0.014523904	7.758226178	6.503712216	9.02040416	14.39160249
TANK	1.036473492	9.00E-05	0.000909087	36.12382436	45.34496069	57.9037915	75.14527637
RDH14	1.036277948	0.000311444	0.002597943	9.952250347	10.45813797	14.8233353	18.50328637
MRPL32	1.034939178	1.09E-05	0.000152243	24.37012346	20.03663589	34.81821761	37.57299264
PFKP	1.03492972	1.37E-06	2.50E-05	64.56778056	93.85258321	128.2958693	129.44786
STK17A	1.034763907	0.000122715	0.001184546	18.10148298	13.88530695	26.42919759	25.66328761
RIF1	1.03413891	0.007650319	0.035368276	11.12762044	12.12778439	14.0033559	24.03279228
ZNF496	1.033937166	3.07E-05	0.00036111	34.94845427	24.95769905	46.23485388	51.39675741
ANKFY1	1.033130356	3.25E-06	5.32E-05	134.854912	87.87700653	175.9808252	186.4443055
GOLT1B	1.02955092	1.30E-06	2.40E-05	21.86266727	24.25469002	34.94436829	39.91239899
NDUFC2	1.02815086	1.83E-05	0.000234499	68.48568086	49.29938644	97.45202875	93.36428943
RNF19A	1.027716025	0.00024623	0.002121397	27.03429566	28.47274416	38.4135119	51.75121292
LILRB1	1.025019582	1.57E-05	0.00020633	163.1421522	119.6002886	220.0074115	237.4858985
SH3GLB1	1.024366128	5.36E-09	1.81E-07	108.60498	115.6458629	170.4932707	192.2573758
NT5C2	1.024245702	1.39E-06	2.52E-05	80.31773977	65.99585071	103.3811106	133.7013261
PROCR	1.022983116	2.25E-05	0.000276785	14.81044672	14.41256371	22.96005397	24.24546558
ZNF101	1.022920686	0.003280651	0.017901329	10.97090443	7.90973026	12.42647244	18.21972197

C8orf59	1.022236468	0.000615207	0.00456233	16.37760684	10.28238571	21.13086915	21.97695034
COX17	1.021915222	1.46E-05	0.000193319	29.93354189	27.41823062	50.65012757	41.68467652
MFSD14B	1.019216715	7.82E-06	0.000114399	59.70958418	50.09027159	74.55568087	102.863697
INTS12	1.018748066	7.37E-05	0.000776553	19.27685307	17.8397327	28.88913579	30.90922911
ADPRHL2	1.018233707	4.72E-05	0.000525442	80.08266575	51.93567027	107.6071582	104.8486478
IKZF5	1.017555881	0.002058054	0.012182026	9.247028293	9.139996049	13.94028056	15.66764232
ARMCX1	1.016998791	7.44E-05	0.000781616	19.82535911	24.1668139	32.98903279	37.78566595
ATXN7	1.016831409	0.003281063	0.017901329	15.12387875	13.00654567	18.54478027	26.86843633
HEBP2	1.016214232	7.96E-05	0.000827273	18.57163101	26.80309773	36.64740243	36.15517061
CUL4B	1.016172802	5.26E-05	0.000578048	24.76191349	18.10336108	30.2137179	38.91992357
TMED7	1.015961216	0.000919198	0.006313636	25.31041953	28.38486803	34.56591626	52.03477732
SMARCA5	1.01475056	4.90E-05	0.000543538	63.39241047	51.93567027	77.33099576	108.464094
FUOM	1.0139262	2.00E-05	0.000250915	47.40737723	37.08460468	68.43737303	67.13458192
ZNF22	1.013836909	0.000447125	0.003499163	16.14253282	13.62167856	20.62626645	27.29378294
SIAH2	1.013185743	2.43E-05	0.000297158	24.68355548	21.53053006	34.88129295	39.34527018
ARID4B	1.012506054	0.003289091	0.017938349	16.76939687	16.34583853	20.37396509	32.96507105
CERS6	1.012182972	4.58E-05	0.000512149	36.28054038	30.58177122	46.23485388	61.2506205
RAB9A	1.011470715	5.55E-06	8.52E-05	68.72075488	49.29938644	92.21677565	96.9797356
IDI1	1.011345113	4.30E-07	9.01E-06	51.56035155	51.84779414	86.91844722	78.47715814
KLF4	1.010645685	0.000656249	0.004799961	20.84401319	29.70300995	46.67638125	33.88665537
MFSD12	1.010615482	6.08E-08	1.53E-06	246.9068606	232.5211128	410.4949338	355.7322556
LINC00998	1.01010899	1.89E-05	0.000240726	26.32907361	22.05778683	36.64740243	40.90487441
MESDC1	1.009878885	2.24E-07	4.99E-06	54.30288177	53.95682121	85.59386511	87.62211021
PRKAA1	1.009274626	0.000749108	0.005362289	22.41117331	16.2579624	24.72616345	37.43121044
GART	1.008956888	3.85E-07	8.20E-06	33.53801017	34.88770148	51.72240833	57.77695653
PPP2R3C	1.008548463	6.52E-06	9.79E-05	29.54175186	35.85433889	51.65933299	52.81457944
GPATCH1	1.00839133	0.007235537	0.033889909	6.896288112	5.361322555	10.02960958	9.571007597

CASP8	1.006923843	0.000801305	0.005640794	30.0902579	18.63061785	34.3136149	43.74051846
ELMO1	1.006254967	2.48E-07	5.52E-06	40.27679868	39.19363174	60.17450369	66.78012641
CALM1	1.004559306	1.66E-09	6.23E-08	402.8392926	339.9936171	552.6036714	632.9873531
PAIP1	1.004440822	1.79E-05	0.000229474	48.19095729	33.74531182	60.55295572	70.32468148
PRKRIP1	1.002181192	0.000384172	0.003097202	19.6686431	20.47601653	32.23212873	31.61814013
LRP10	1.001498147	1.05E-08	3.20E-07	253.4889331	256.7749241	410.6210844	400.6772139
MEMO1	1.000618979	1.12E-06	2.11E-05	32.44099808	29.96663833	45.79332651	53.59438155
TLR1	1.000563584	3.03E-06	5.03E-05	50.77677149	57.82337083	85.53078977	87.0549814
RPS18	-1.000103209	1.62E-05	0.00021214	599.0477398	653.1841364	273.5583739	222.7405494
PDXDC2P-NPIPB14P	-1.00035046	0.009867119	0.043465322	9.403744305	15.29132499	5.235883849	4.537739399
SLC35E1	-1.000579593	4.31E-05	0.000487297	33.77308418	44.81770392	13.56182853	17.72348426
SDHAP1	-1.002393767	0.000242865	0.002099929	25.15370352	26.36371709	9.272705514	11.20150293
IPO4	-1.005295744	0.000241075	0.00209052	19.43356908	19.94875976	8.452726113	7.089819049
CEP131	-1.006188494	0.009324584	0.041467817	14.41865669	16.60946691	7.380445359	4.821303805
SLC36A1	-1.007317255	7.67E-05	0.000801682	142.1422065	235.6846534	64.96822942	84.71557506
TP53BP1	-1.010322395	0.000776638	0.005523739	15.28059476	19.59725525	5.992787912	7.798730063
SPRY2	-1.011070454	0.001375966	0.008830838	11.12762044	15.02769661	4.920507157	5.388432616
UNG	-1.01148915	0.002461071	0.014217662	9.795534335	9.403624432	3.406699033	4.183283892
TTLL12	-1.011531309	0.000561085	0.004226001	33.30293615	62.65655786	18.04017757	19.7793262
FAM193B	-1.011871392	0.000220143	0.001934769	49.28796938	45.16920843	21.95084855	15.1005135
NFKBIA	-1.011952545	0.005376337	0.026781469	296.1940464	883.4195912	267.9446687	195.2348021
WDR19	-1.013782401	0.008423507	0.038298559	9.717176329	11.68840375	4.163603095	4.254174994
CARD19	-1.014722953	4.42E-05	0.000496859	123.2579271	155.7173772	60.3637297	49.05735106
SIGLEC9	-1.015656732	5.97E-07	1.21E-05	95.51919294	76.80461443	33.43056016	34.31200198
SETD7	-1.0220496	0.000862581	0.005978821	24.84027149	41.03903043	11.29111635	14.53338469
NBPF8	-1.023130269	9.43E-05	0.000946029	37.22083645	50.4417761	17.15712283	17.08546434
FBXL20	-1.023332302	0.000535739	0.004091209	20.76565518	25.66070807	7.443520697	10.77615632

SGSM2	-1.023479323	5.35E-05	0.000584933	56.49690594	89.98603359	29.51988917	27.64823845
ACTN1	-1.023977443	0.001065632	0.007149293	73.57895125	168.8109202	42.19803221	52.67279724
LOC148413	-1.024559993	0.001793495	0.010887049	18.57163101	23.02442424	8.957328821	7.231601252
MEGF8	-1.025087532	1.63E-05	0.00021333	28.8365298	33.74531182	11.48034236	12.97378046
PHC2	-1.025139138	3.87E-06	6.21E-05	177.8734573	251.7659848	85.02618706	82.58884202
GBE1	-1.025157506	0.001613107	0.009986592	21.23580322	44.20257103	9.777308222	15.88031562
AK1	-1.027056625	0.000791296	0.005590514	9.011954275	9.667252815	3.659000387	3.616155081
MAST2	-1.028459383	0.000306364	0.002564468	31.50070201	49.65089095	13.87720522	17.79437536
IMPA2	-1.02921658	0.000747413	0.005352815	27.89623373	29.8787622	12.42647244	9.996354205
TM6SF1	-1.030202569	0.000733984	0.005277588	17.31790291	29.17575318	7.506596036	10.63437412
HADH	-1.030295151	0.001699657	0.010428467	22.17609929	13.00654567	7.632746713	6.026452528
PLXNA3	-1.030387541	0.000779908	0.005541533	30.16861591	56.24160053	17.72480087	15.80942452
BHLHE40	-1.031425456	0.003026139	0.016835086	27.11265367	66.78673586	20.75241712	15.59675121
FCGR2C	-1.033404844	5.02E-05	0.000555116	66.05658267	44.37832328	21.13086915	21.76427703
C3	-1.034788097	0.001565968	0.009757551	53.91109174	128.9151582	38.60273792	32.11437784
SRSF8	-1.034802293	0.00037573	0.003041028	15.28059476	17.92760883	6.749691974	6.09734363
MKKS	-1.036469467	1.93E-05	0.000244567	33.61636817	46.75097873	15.70639004	15.38407791
SNX30	-1.036471469	0.001159445	0.007661147	30.79547995	54.13257346	13.37260252	19.63754399
CNTROB	-1.037184349	1.22E-05	0.0001661	36.20218237	39.28150787	14.06643124	15.17140461
RAB27A	-1.038413898	0.001073386	0.007190476	12.14627452	16.78521916	5.172808511	6.026452528
MZT2A	-1.040334203	0.003315905	0.018041997	27.03429566	28.73637254	13.94028056	7.444274556
PDPR	-1.041613376	1.86E-05	0.000237396	40.04172467	49.29938644	16.08484207	18.43239527
ARHGAP19	-1.043320724	0.00098743	0.006724679	14.88880473	19.86088364	6.245089266	7.16071015
BRD8	-1.044908134	0.00019826	0.001779289	30.01189989	43.93894264	15.01256132	13.39912707
ADAM28	-1.045287813	0.000727975	0.005250078	37.22083645	74.60771123	21.19394449	21.83516814
HMGB2	-1.045369352	5.69E-05	0.000615034	45.76185911	45.16920843	18.22940358	16.73100884
CCDC57	-1.045544732	0.003270884	0.017874183	15.98581681	20.03663589	8.389650775	5.388432616

CROCCP2	-1.047272985	0.002437343	0.014114484	17.55297693	12.65504116	5.740486558	5.884670325
CKAP2	-1.049457438	0.004169829	0.021849967	14.65373071	12.56716503	4.668205803	5.813779224
TBC1D14	-1.049716942	1.63E-06	2.90E-05	112.9146703	159.8475552	49.26247013	55.50844129
ARRB1	-1.049726107	5.65E-07	1.15E-05	170.8995948	244.7358946	83.57545428	75.71240518
SUMF1	-1.049760478	1.39E-05	0.000186545	42.78425488	59.22938887	18.60785561	20.55912831
RPS28	-1.049870507	0.000245006	0.002114644	324.4029286	437.4482428	174.7193184	116.4038974
DEF8	-1.052171869	1.51E-07	3.50E-06	97.71321711	114.1519687	42.89186094	38.14012145
RGL2	-1.05233532	3.45E-05	0.000398729	74.98939536	110.900552	38.09813521	32.96507105
ACACA	-1.053287119	0.000633212	0.00466933	15.59402678	18.36698946	5.866637235	7.16071015
AHNAK	-1.05347304	0.00065944	0.004820863	683.2825963	1379.040952	287.2457223	506.3758461
ELK3	-1.053946511	0.001941092	0.011596293	14.02686666	21.88203457	7.31737002	6.380908035
SLC7A6	-1.055005023	0.001317917	0.008532463	18.49327301	36.99672855	11.16496567	9.996354205
MFSD3	-1.055982967	0.003625627	0.019443115	11.28433645	13.62167856	5.362034526	4.112392791
ITGAX	-1.056812729	4.29E-06	6.75E-05	410.9885253	666.8049362	213.0691242	198.1413373
H2AFZ	-1.057705666	1.65E-08	4.72E-07	143.7877247	164.4171138	60.11142835	57.42250103
MYO19	-1.059459784	2.02E-05	0.000253493	30.87383796	30.58177122	10.84958898	12.61932496
SLC19A1	-1.060204222	0.007762821	0.035767968	7.52315216	10.8975186	2.460568955	4.608630501
NME1	-1.062013092	5.64E-06	8.64E-05	49.05289536	55.80221989	20.50011577	19.35397959
VCL	-1.06289558	0.001588831	0.009870178	22.56788932	44.81770392	9.588082206	16.16388003
CCNF	-1.064395762	0.00061189	0.004549294	11.36269446	11.24902312	3.53284971	5.10486821
REV3L	-1.06546669	0.006451098	0.030969991	11.44105246	17.40035206	4.668205803	6.310016934
PAN2	-1.068021211	0.00049981	0.003852971	15.12387875	20.3881404	6.371239943	7.089819049
FURIN	-1.068406587	1.98E-05	0.000249783	156.0115736	273.20776	85.65694045	76.7048806
TGM2	-1.068824371	1.73E-05	0.000224278	13.47836062	17.04884755	5.929712573	5.60110592
FAH	-1.069265585	6.39E-06	9.63E-05	76.86998751	80.14390728	26.68149894	32.89417995
DIP2A	-1.0702704	0.00013793	0.001307798	35.88875035	43.32380975	17.15712283	12.69021606
WDR6	-1.071092955	1.25E-05	0.000169859	103.1199195	155.3658726	52.79468908	44.73299388

TNKS1BP1	-1.071306201	0.005316022	0.026581953	14.96716273	42.44504847	11.10189033	10.56348302
SNHG7	-1.07177446	0.001709401	0.010470446	19.98207512	25.04557517	9.714232883	7.231601252
SIGLEC15	-1.07341697	0.002656808	0.015088702	3.76196787	8.876367665	2.334418278	2.411006358
IVNS1ABP	-1.074411526	1.52E-08	4.40E-07	118.3997307	128.6515298	44.02721703	49.27002437
TXNDC5	-1.07460412	7.94E-08	1.96E-06	60.25809022	72.76231255	23.90618405	26.30130752
MAN2A2	-1.075396611	8.11E-07	1.58E-05	58.37749808	77.06824281	25.10461548	25.94685202
CLEC4A	-1.077525809	6.76E-07	1.35E-05	47.0155872	42.62080073	16.40021877	17.36902875
GINS3	-1.078212187	0.008776039	0.039503595	8.071658202	8.349110899	2.523644294	3.687046183
SNHG6	-1.08057232	2.62E-05	0.000315642	36.67233041	30.84539961	13.68797921	11.62684954
PLD2	-1.080630719	3.23E-05	0.000377637	39.1797866	52.9901838	16.08484207	18.57417747
KCTD20	-1.080956468	5.75E-07	1.17E-05	83.68713403	105.8037366	30.46601925	40.90487441
RGS12	-1.081623127	6.48E-06	9.74E-05	44.66484702	42.88442911	18.16632824	14.60427579
MS4A14	-1.082955973	0.00117563	0.007753895	32.28428207	25.30920356	9.272705514	12.40665165
DCANP1	-1.083076907	0.00284968	0.015989011	15.12387875	19.15787461	7.001993328	5.813779224
BCAT1	-1.083558938	7.30E-05	0.000771062	52.03049959	98.33426573	29.26758782	27.01021854
SNHG1	-1.084091086	0.000285268	0.002417323	33.30293615	22.76079585	11.60649304	9.358334293
ANAPC15	-1.085978843	0.000364056	0.002959826	26.01564159	36.64522404	12.67877379	10.70526522
PLXNB2	-1.088418834	1.27E-09	4.88E-08	482.5293848	559.8596887	209.4107546	179.3551954
PYGL	-1.08917252	2.51E-07	5.58E-06	91.0527866	121.885068	35.57512167	44.09497397
PKD1P4-NPIPA8	-1.089230835	0.004143045	0.021749102	22.56788932	15.81858176	8.326575436	5.955561427
CCNE1	-1.089849157	0.005783437	0.028436379	7.91494219	6.064331577	2.334418278	2.907244068
ZMYND8	-1.089924228	0.000191851	0.001729295	27.97459174	43.58743813	14.57103395	12.05219615
SNRNP200	-1.090034696	5.67E-07	1.15E-05	122.552705	177.0712762	54.68694924	57.13893662
SPECC1	-1.091765106	1.90E-05	0.000241306	18.25819899	17.92760883	6.749691974	6.735363542
GOLGA7B	-1.092650912	0.008590416	0.03887372	8.30673222	17.40035206	4.605130464	4.963086007
DPM3	-1.093551027	0.002569057	0.014706094	12.38134853	17.31247593	5.109733172	5.955561427
TNFRSF9	-1.09461864	0.004691487	0.023974097	5.094053973	10.19450958	2.965171663	2.694570763

WRB	-1.095061223	0.000810503	0.005696169	13.55671863	12.30353665	4.731281141	4.892194906
PPARG	-1.097373513	0.006360947	0.030731445	16.84775488	32.16354152	9.714232883	8.436749975
SPTAN1	-1.097406761	2.79E-05	0.000333274	104.5303636	188.5830489	55.63307932	53.09814384
PLEK2	-1.098210001	0.000788101	0.005577778	9.168670287	13.88530695	4.415904449	4.112392791
MID1IP1	-1.098743247	1.08E-05	0.000151782	50.14990745	73.5531977	25.7984442	19.9211084
CUL7	-1.099277083	0.000138078	0.00130834	17.70969295	19.59725525	6.812767312	7.018927947
LOC100506585	-1.099824526	0.004744678	0.02422018	9.090312281	12.39141278	3.848226402	4.112392791
SNAPC1	-1.100006257	0.003205161	0.01764061	10.81418841	18.8063701	5.298959188	5.671997021
44085	-1.104199366	5.52E-05	0.000601475	44.19469899	44.99345618	12.11109575	21.05536602
P3H1	-1.105510552	1.64E-05	0.000213636	49.44468539	58.52637985	22.83390329	16.87279104
ATP13A3	-1.10615974	0.001236193	0.008076524	69.26926092	136.6482574	30.2137179	46.07992481
LRRC25	-1.107076953	3.27E-08	8.67E-07	254.1157972	237.0027953	100.1642683	80.53300008
CHAF1A	-1.109768112	0.000402828	0.003220618	25.23206152	26.53946935	11.10189033	7.869621164
SPR	-1.109908721	2.22E-05	0.000274446	21.31416122	27.3303545	7.948123405	9.996354205
SDC2	-1.11154117	9.66E-08	2.36E-06	173.485409	244.9116468	72.78957139	81.17101999
LOC100288778	-1.1122311	0.000862395	0.005978821	44.89992104	77.41974732	27.81685503	16.87279104
SDC4	-1.11502504	0.000994651	0.006761099	12.53806455	28.82424867	7.75889739	7.373383454
PIDD1	-1.115440025	0.005702146	0.028180711	10.10896636	14.58831597	5.046657834	3.970610588
NETO2	-1.116908342	0.00073196	0.00526829	16.06417482	18.45486559	4.983582495	7.727838961
RUSC2	-1.119657488	0.000368662	0.002993899	26.1723576	52.81443155	13.37260252	15.52586011
SSH2	-1.120295989	0.000112723	0.001099865	23.89997542	31.10902799	10.15576025	9.925463104
TPCN1	-1.127562951	4.37E-10	1.86E-08	166.8249784	174.9622491	65.47283212	58.62764975
SMPD2	-1.129450207	0.000791759	0.005590514	16.53432285	23.11230036	5.740486558	8.720314381
APBB3	-1.129938802	8.19E-06	0.000119056	52.57900563	56.94460955	22.32930058	17.29813765
DDB1	-1.131734606	5.22E-07	1.08E-05	149.2727851	233.2241218	67.86969499	70.82091919
MRPS34	-1.132505629	1.29E-06	2.38E-05	84.00056606	90.3375381	33.30440949	29.77497149
LAMC1	-1.132708386	7.49E-07	1.48E-05	39.96336666	32.69079829	11.66956838	14.746058

PTGR1	-1.134243433	8.84E-05	0.000897451	49.28796938	85.15284657	25.73536886	22.82764355
TNIK	-1.139629958	0.000330096	0.002733373	25.31041953	45.34496069	11.5434177	13.96625588
CLEC2D	-1.1414308	0.00080999	0.005696169	14.02686666	14.0610592	4.542055126	5.60110592
LPAR1	-1.141935947	0.007096326	0.033400875	5.564202009	11.77627988	2.839020986	3.403481777
ATP2B1	-1.144186916	0.001802622	0.0109301	19.19849506	37.7876137	7.885048067	12.69021606
FANCG	-1.144204599	0.001554866	0.009705138	13.2432866	11.42477537	4.605130464	4.254174994
PI4KAP1	-1.144706324	0.005855414	0.0287511	10.5791144	20.82752104	6.812767312	4.395957196
TGFBR1	-1.146777537	0.000423391	0.003354331	44.50813101	104.046214	22.26622525	31.19279352
EPAS1	-1.146878092	2.31E-05	0.000283461	69.58269294	115.1186061	28.51068376	37.92744815
EEF1E1	-1.148668204	0.002042281	0.012103553	12.45970654	8.612739282	3.53284971	4.04150169
CAD	-1.149975318	2.11E-05	0.000262962	25.70220956	25.39707969	10.21883559	8.011403367
ZNF692	-1.150371176	0.000128851	0.001234692	17.31790291	17.31247593	6.623541297	5.742888123
PCCA	-1.150650287	0.000384891	0.003101264	15.67238479	20.03663589	7.191219343	5.530214819
ATP6V0D2	-1.153385183	0.002549807	0.014610934	12.14627452	39.28150787	8.515801452	9.854572003
FADS3	-1.153416777	0.000199833	0.001790058	20.84401319	32.60292216	9.903458899	9.145660989
NSD2	-1.155773871	3.74E-06	6.03E-05	40.5118727	47.54186388	14.63410929	16.80189994
PIK3IP1	-1.155975275	8.43E-05	0.000863712	21.78430926	18.01548495	7.506596036	6.664472441
MAPKAPK5-AS1	-1.157246479	0.000385126	0.003101425	15.43731077	14.93982048	6.434315281	4.325066095
ENKD1	-1.15824686	0.002782705	0.015659909	12.22463252	18.89424623	5.803561896	5.246650413
NBPF15	-1.164643395	5.29E-05	0.000580918	31.10891198	42.79655298	14.0033559	12.12308725
NBPF20	-1.16641137	0.008758211	0.039472518	13.79179264	16.52159078	3.785151064	7.018927947
MKL2	-1.166692438	0.005902313	0.028961674	6.817930106	9.49150056	2.523644294	3.261699574
HMGB3	-1.167875755	0.000473384	0.003680717	11.83284249	22.76079585	6.245089266	5.955561427
PPIEL	-1.169933029	0.004776243	0.024350378	12.06791651	22.76079585	7.128144005	5.10486821
CDKN3	-1.170924264	0.009694054	0.042873091	7.679868172	8.70061541	2.334418278	3.474372879
LOC102724814	-1.172736205	0.004764626	0.024304811	8.855238263	13.79743082	4.605130464	3.332590676
ENOSF1	-1.17344287	1.40E-06	2.54E-05	45.05663705	63.62319526	18.16632824	20.1337817

FBLN5	-1.175115652	8.53E-05	0.000872043	34.0081582	44.81770392	10.84958898	17.01457324
SNHG8	-1.18163335	0.000875999	0.006054378	27.42608569	16.34583853	8.831178144	6.451799136
RFX2	-1.181695029	0.008898898	0.040006231	8.620164244	11.33689924	3.911301741	3.04902627
FAM129B	-1.181849787	9.80E-06	0.000139363	323.4626325	614.6943925	161.410422	167.161926
TXLNGY	-1.182468623	0.000377266	0.003048338	15.59402678	14.6761921	4.542055126	6.09734363
BDH1	-1.182837729	0.000739451	0.005308964	4.937337961	8.876367665	2.27134294	2.552788561
XPNPEP3	-1.183132791	0.001317027	0.008530526	9.247028293	7.382473493	2.649794971	3.190808473
NAV1	-1.185749386	0.004991428	0.025257346	11.9112005	34.79982536	7.191219343	9.145660989
GNA12	-1.186776765	2.72E-07	5.95E-06	119.9668908	184.2771187	54.93925059	51.113193
PDGFC	-1.188134139	0.008333789	0.037962406	5.799276027	9.667252815	2.019041586	3.403481777
NBPF1	-1.188512318	3.01E-05	0.000355145	22.64624733	18.8063701	7.75889739	6.664472441
KIFC2	-1.189397091	0.000263907	0.002250853	30.16861591	47.45398775	16.52636944	10.35080971
OLFML2B	-1.190065076	2.85E-08	7.69E-07	259.3657836	382.7892913	114.7346715	108.6767673
PRKAR2A	-1.190859328	2.26E-06	3.89E-05	25.46713554	34.97557761	10.78651364	10.20902751
ST14	-1.191922637	0.000955945	0.006534933	92.3848727	254.1386403	64.08517468	56.2173523
LINC01128	-1.192399326	0.003755593	0.020013651	8.228374214	16.69734304	4.79435648	3.828828385
CAPS	-1.192996139	0.002715471	0.015361411	10.50075639	19.50937913	6.245089266	4.112392791
CERK	-1.19430972	7.51E-06	0.000110315	43.64619294	76.10160541	19.61706103	21.97695034
ABHD2	-1.195932428	9.37E-09	2.92E-07	96.53784702	120.4790499	33.11518347	42.32269643
TUBGCP6	-1.197533286	2.85E-06	4.77E-05	34.39994823	48.94788193	14.31873259	14.53338469
MXI1	-1.200790047	0.00110858	0.007392752	10.42239838	20.30026427	5.551260542	5.033977109
ZMIZ1	-1.201965759	3.13E-05	0.000366431	140.1048984	265.2989085	59.98527767	80.39121787
ARHGEF9	-1.203383228	7.98E-05	0.000828831	10.50075639	10.28238571	3.09132234	4.112392791
ZAK	-1.204907557	0.000299175	0.002513037	15.98581681	24.69407066	6.245089266	7.798730063
DFNA5	-1.205921592	0.000252768	0.002168668	34.94845427	81.4620492	18.16632824	21.97695034
KLHL21	-1.209046599	5.67E-05	0.000613912	38.94471258	73.90470221	19.86936238	18.85774188
GNG2	-1.209551869	9.33E-06	0.000133068	13.00821258	14.14893533	3.848226402	5.530214819

PLEKHM3	-1.209766144	0.004955418	0.025118242	10.03060835	15.73070563	4.037452418	4.821303805
RCCD1	-1.209783737	0.001408403	0.008993215	13.79179264	13.35805018	3.974377079	5.388432616
FHOD1	-1.210164449	7.15E-09	2.33E-07	96.61620503	106.067365	33.61978618	36.01338841
PHLDB1	-1.211714955	9.38E-05	0.000942895	15.98581681	24.25469002	7.443520697	6.310016934
MAP3K4	-1.213742025	6.85E-05	0.000729992	19.12013705	19.15787461	7.254294682	5.813779224
TBC1D9	-1.214196833	9.72E-05	0.000970752	19.35521107	35.76646276	9.083479498	9.783680901
PRKDC	-1.214279887	2.47E-05	0.00029979	35.3402443	51.05690899	11.16496567	18.57417747
SOCS7	-1.216926375	0.002239417	0.013105042	7.993300196	10.98539473	3.217473017	3.261699574
ST6GALNAC6	-1.218333944	8.13E-05	0.000840058	19.82535911	36.82097629	9.335780852	9.996354205
TUBGCP5	-1.219581145	0.000911506	0.006269756	9.795534335	12.65504116	4.289753772	3.332590676
SLX1A	-1.219707493	0.000814287	0.005704355	38.86635458	38.66637498	7.632746713	19.07041518
PDP2	-1.221722041	0.003329518	0.018090467	4.858979955	6.152207705	1.766740232	1.98565975
THEM6	-1.22237977	9.95E-05	0.000991185	23.6649014	15.6428295	7.065068666	6.310016934
DNHD1	-1.22445611	0.003207951	0.017649236	11.12762044	8.436987027	3.659000387	2.978135169
PLCB2	-1.231013716	4.00E-14	4.21E-12	192.7614784	194.2071211	62.19291452	68.90685945
P2RX1	-1.234462482	0.003231177	0.017729703	8.855238263	10.63389022	3.848226402	2.694570763
CYP1B1	-1.235374987	0.000188083	0.001701712	205.9256235	425.4970894	83.4493036	130.5112265
ZBTB48	-1.237251482	2.91E-07	6.33E-06	56.57526394	57.47186632	20.56319111	17.79437536
CH17-340M24.3	-1.237333406	0.005746081	0.028312638	11.44105246	13.79743082	5.929712573	2.481897459
PCED1B-AS1	-1.237490976	0.006645146	0.031711674	6.347782069	11.60052763	2.586719632	3.474372879
HS6ST1	-1.241001507	1.28E-05	0.00017335	23.35146938	26.80309773	7.001993328	9.925463104
MXD4	-1.242582678	3.19E-06	5.24E-05	57.43720201	79.35302213	26.05074556	19.70843509
LTA4H	-1.245081281	1.22E-06	2.27E-05	56.57526394	95.25860126	23.78003337	27.15200074
LTB4R	-1.246271177	0.001348826	0.008680247	12.06791651	10.72176635	4.542055126	3.04902627
EMC9	-1.249655103	0.001754571	0.010692659	9.482102311	11.5126515	3.53284971	3.474372879
ALOX5AP	-1.250590047	6.15E-06	9.32E-05	64.88121258	99.30090314	25.7984442	29.06606048
HCCS	-1.252525106	6.98E-07	1.39E-05	29.14996183	25.30920356	9.02040416	9.145660989

SLC4A7	-1.253095873	0.000710102	0.005142036	24.84027149	35.59071051	7.695822051	12.54843385
ALDOC	-1.253410222	0.000282808	0.002402499	10.42239838	17.8397327	4.920507157	4.466848298
PAPSS2	-1.253729021	0.000572284	0.004294658	9.403744305	18.89424623	4.79435648	4.608630501
UCK2	-1.254682384	0.000198979	0.00178352	11.59776847	17.75185657	4.731281141	5.033977109
MYO15B	-1.254879739	1.55E-05	0.000204549	81.17967784	51.49628963	25.92459488	18.07793976
ZFPM1	-1.256732272	0.008497258	0.038551712	6.504498081	8.349110899	2.586719632	2.340115256
STAB1	-1.257355199	3.07E-11	1.70E-09	3440.465755	4185.980225	1306.03859	1226.558545
PPME1	-1.260364654	6.04E-06	9.17E-05	22.95967935	27.06672611	6.875842651	9.783680901
PELI3	-1.261180642	0.000183454	0.001666097	18.414915	21.0032733	6.938917989	6.09734363
NBPF11	-1.261461593	1.85E-05	0.000235766	28.52309778	39.01787949	11.16496567	11.20150293
HERC2P9	-1.265258437	0.000540525	0.004115873	14.73208872	21.70628232	5.803561896	6.239125832
PPIP5K1	-1.266892786	0.001136903	0.007560667	6.896288112	10.10663345	2.902096325	2.694570763
HACD3	-1.268313496	2.77E-09	9.95E-08	33.53801017	34.97557761	10.91266432	11.69774064
ACSF2	-1.26967762	1.05E-07	2.53E-06	41.68724279	45.43283682	15.51716403	13.11556267
TNFAIP8L1	-1.270047966	1.44E-05	0.000191637	24.9186295	35.67858663	11.22804101	8.649423279
CDKN2C	-1.271253366	0.00033176	0.002743624	12.06791651	15.02769661	5.046657834	3.828828385
ZHX3	-1.273262031	0.000296045	0.002491097	11.20597844	10.37026184	3.280548356	3.828828385
HOOK2	-1.274300177	0.001034489	0.006979292	11.83284249	12.21566052	4.731281141	3.119917372
FGR	-1.274556115	1.95E-05	0.000246249	127.4109014	273.9986451	62.44521588	69.47398826
SELPLG	-1.275339395	3.57E-11	1.91E-09	190.4890963	247.5479307	67.42816762	76.4922073
FILIP1L	-1.27689871	0.002659958	0.015100651	5.094053973	7.558225749	2.082116924	2.056550851
NPHP4	-1.280114417	0.000758855	0.005414132	12.22463252	18.19123721	5.109733172	4.821303805
ABCA7	-1.28070882	1.03E-05	0.000145047	50.77677149	47.62974001	18.92323231	13.11556267
HERC1	-1.281233744	6.21E-06	9.39E-05	39.10142859	58.3506276	13.24645184	18.71595967
ZNF767P	-1.283082835	0.003979774	0.021014314	7.444794154	7.11884511	2.145192263	2.623679662
STXBP5	-1.284529894	0.001716849	0.010500502	12.06791651	23.11230036	5.109733172	6.380908035
APMAP	-1.285704981	3.87E-09	1.34E-07	227.1606431	341.8390158	88.55840602	96.9797356

UBE2T	-1.290354193	0.009875096	0.043473918	4.1537579	5.712827066	1.703664893	1.48942204
CNNM3	-1.290862951	3.50E-05	0.000404593	19.43356908	20.56389266	7.065068666	5.884670325
PFKFB2	-1.295060818	0.000228068	0.001995909	13.40000261	10.28238571	2.775945648	4.963086007
USE1	-1.295478727	0.000793515	0.005596883	17.39626092	25.30920356	8.957328821	4.750412703
ZNF219	-1.295757049	2.46E-05	0.000299338	32.98950412	54.39620185	14.8233353	13.39912707
MPRIP	-1.295776942	1.34E-06	2.47E-05	42.00067482	68.54425842	18.4186296	17.29813765
PLAUR	-1.298794766	4.39E-06	6.91E-05	296.2724044	576.8197814	153.3367787	128.030038
FAM20C	-1.298948216	2.26E-05	0.000277435	232.8024195	518.9094132	127.2866639	115.1278575
ENDOG	-1.299898424	0.000783813	0.005561004	12.06791651	20.12451202	5.803561896	4.537739399
AHRR	-1.302351767	4.41E-05	0.0004966	89.7990585	201.676592	42.63955958	51.39675741
TUBB3	-1.303210212	8.88E-05	0.000898978	11.28433645	15.37920112	4.983582495	3.54526398
FAM83H	-1.3069316	0.003644356	0.01952179	8.071658202	23.28805262	4.478979787	5.60110592
SLC25A10	-1.307334148	0.001812702	0.010981988	9.873892341	7.470349621	3.406699033	2.127441952
HLA-DMB	-1.307346938	1.84E-06	3.23E-05	76.16476545	108.8794011	31.97982738	27.29378294
MOB3B	-1.307933446	0.003307423	0.018004309	7.13136213	3.867428383	1.766740232	1.772986445
CEP55	-1.310051698	0.007965787	0.036574699	8.150016208	10.19450958	2.965171663	2.907244068
FAM173B	-1.31009823	0.00267176	0.015155728	5.250769985	7.294597366	1.892890909	2.127441952
LPIN3	-1.310655889	0.001013593	0.006863992	6.817930106	9.052119921	2.523644294	2.552788561
HERC2P2	-1.312569609	5.94E-06	9.05E-05	51.56035155	82.25293435	20.68934178	22.11873254
IFFO2	-1.313257206	0.003212764	0.017662348	8.228374214	7.997606388	1.82981557	3.403481777
RCC1	-1.314006761	2.59E-08	7.07E-07	32.44099808	36.82097629	10.7234383	11.41417623
ASB13	-1.316005175	6.13E-07	1.24E-05	25.38877754	25.48495581	8.011198744	8.224076671
FADS1	-1.316385395	1.03E-08	3.17E-07	103.6684256	88.66789168	25.98767022	35.5880418
AKR1B1	-1.317911173	0.000563138	0.004239252	71.6200011	214.3307544	45.09949779	46.00903371
SLC11A1	-1.318739028	5.86E-06	8.94E-05	147.705625	310.379362	73.10494808	72.73497893
HERC2	-1.319855718	1.22E-07	2.89E-06	44.74320503	61.33841594	16.52636944	17.22724655
MCM6	-1.320949487	9.80E-08	2.38E-06	37.37755246	29.96663833	9.777308222	11.69774064

ATAD2	-1.321659899	0.005445432	0.027076286	8.30673222	8.70061541	1.703664893	3.757937284
LMF1	-1.322625746	0.000250074	0.002149962	12.92985458	22.67291972	4.478979787	6.877145745
PHF19	-1.324113252	7.66E-09	2.47E-07	49.67975941	53.6053167	15.6433147	17.15635544
LINC01089	-1.324603804	0.004121732	0.021676644	7.836584184	6.240083833	2.334418278	2.127441952
COL23A1	-1.327614455	9.57E-08	2.34E-06	47.64245125	50.09027159	17.15712283	13.68269148
NCAPD3	-1.328893854	7.87E-06	0.000115031	19.04177905	22.32141521	6.749691974	6.310016934
NSUN5P2	-1.32920258	0.004522227	0.023284958	6.269424063	5.273446427	2.208267601	1.418530939
PPIF	-1.330630265	3.13E-07	6.79E-06	285.6940736	474.7955971	128.989698	110.8035004
RTL6	-1.331426263	3.08E-05	0.000361652	17.94476696	28.73637254	6.875842651	7.869621164
MSC-AS1	-1.333930277	0.008001325	0.036691172	5.720918021	13.88530695	2.712870309	3.474372879
LSP1	-1.33491453	2.21E-05	0.000273487	394.533344	977.0076672	219.8812608	211.8233198
AFG3L1P	-1.335066046	8.10E-07	1.58E-05	32.91114612	38.05124208	11.60649304	10.70526522
KCTD7	-1.336894713	1.36E-05	0.000183284	20.13879113	28.03336352	5.803561896	9.429225394
NECTIN1	-1.339772629	0.001468229	0.009272654	5.329127991	8.964243793	2.208267601	2.269224155
PFKM	-1.340595788	8.49E-07	1.65E-05	16.53432285	19.94875976	5.425109865	6.026452528
CYFIP2	-1.340737494	0.007728547	0.035647848	3.840325876	4.834065788	1.766740232	0.922293229
KCP	-1.3414283	0.001576102	0.009799539	10.50075639	16.78521916	4.289753772	4.254174994
IRAK3	-1.342816609	0.000110336	0.001080964	18.72834702	34.09681633	6.749691974	9.854572003
PFAS	-1.34364143	0.000125006	0.001201844	10.89254642	10.80964248	3.722075725	3.04902627
SDHAP2	-1.344662666	8.08E-05	0.000836722	20.53058116	19.68513138	5.866637235	6.735363542
DUSP7	-1.34632775	7.06E-06	0.000105034	15.51566878	16.08221014	3.974377079	5.955561427
MEGF6	-1.346582567	0.00382091	0.020316711	4.623905936	11.86415601	2.649794971	2.481897459
S1PR1	-1.34698112	0.000157306	0.001459861	9.952250347	9.139996049	2.586719632	3.403481777
TRPV4	-1.347602683	4.88E-06	7.63E-05	15.98581681	19.59725525	6.434315281	4.608630501
LIG1	-1.349057	7.42E-06	0.000109541	28.05294974	24.60619454	8.200424759	8.224076671
KCNK13	-1.351126457	0.000737201	0.005295444	10.73583041	24.25469002	5.488185203	5.388432616
NBEAL2	-1.351952509	1.26E-07	2.96E-06	32.98950412	37.87548983	12.86799981	9.074769887

CDA	-1.353701215	0.008235859	0.037623197	2.351523761	8.349110899	2.145192263	1.134966533
SLC43A2	-1.355677936	3.27E-05	0.000381985	222.7725948	567.8564164	123.6913696	121.6498389
CYTOR	-1.358640904	0.002137903	0.012592684	9.952250347	7.030968982	2.775945648	2.481897459
ITGAM	-1.359083297	1.86E-13	1.68E-11	117.7728667	144.3813567	38.98118995	42.25180533
TTC3	-1.359377576	6.87E-07	1.37E-05	32.36264007	43.06018137	11.10189033	12.26486945
PDPN	-1.362938099	1.17E-09	4.54E-08	54.85138781	73.64107383	20.68934178	18.92863298
ZNF318	-1.363730697	0.000109902	0.001077446	13.87015065	26.7152216	5.866637235	6.664472441
LOC100129034	-1.363916579	2.13E-07	4.79E-06	86.42966424	148.072154	31.91675204	40.62131
NBPF26	-1.364234068	5.94E-06	9.05E-05	67.7021008	107.7370114	22.89697863	31.40546682
F13A1	-1.364491578	7.91E-09	2.54E-07	74.59760533	71.53204676	16.58944478	28.78249607
WDR76	-1.372167944	0.006434563	0.030942078	6.896288112	4.39468515	1.766740232	1.702095344
SLC9A7	-1.3722698	2.00E-05	0.000250915	12.06791651	23.37592875	4.79435648	6.09734363
GINS4	-1.372959525	0.006215549	0.030208474	5.799276027	5.361322555	1.388288201	2.056550851
NME4	-1.373349969	1.84E-09	6.83E-08	74.28417331	52.9901838	19.68013637	19.35397959
KRT10	-1.376901676	0.000111417	0.001089339	9.482102311	11.77627988	2.902096325	3.616155081
DISP1	-1.376979261	0.000101273	0.001004216	17.00447089	24.69407066	6.49739062	6.239125832
ISYNA1	-1.379434297	0.009069503	0.040558414	4.702263942	3.340171616	1.451363539	0.99318433
PHF10	-1.381032304	5.25E-06	8.14E-05	13.00821258	12.83079341	3.911301741	3.970610588
CENPO	-1.381433653	0.000176584	0.001609777	10.42239838	11.86415601	3.722075725	3.04902627
SKI	-1.38226948	7.04E-05	0.000748454	16.61268086	28.9121248	5.362034526	8.578532178
UACA	-1.382276242	0.000557054	0.00420882	10.89254642	15.20344886	3.974377079	3.970610588
TMEM106C	-1.383536854	2.39E-06	4.09E-05	21.31416122	23.28805262	7.506596036	6.026452528
RNF215	-1.384547041	0.00138495	0.0088738	6.347782069	9.49150056	2.334418278	2.481897459
NINJ2	-1.387403448	0.00314785	0.017391507	6.7395721	7.030968982	1.388288201	2.836352966
DNAAF5	-1.389162943	5.31E-05	0.000582652	11.59776847	18.89424623	4.289753772	4.963086007
SMAD7	-1.391012867	0.000605158	0.004507417	13.47836062	33.30593118	6.118938589	8.082294468
TMC8	-1.391297223	1.49E-08	4.33E-07	50.93348751	40.07239302	13.81412989	13.75358258

NUSAP1	-1.391348322	0.00060532	0.004507417	14.02686666	20.47601653	3.848226402	6.664472441
PRPF8	-1.39821083	8.92E-12	5.55E-10	174.8958531	239.9905837	58.34531887	66.85101751
GRAMD4	-1.401149703	8.03E-15	9.42E-13	163.5339422	177.7742852	49.89322351	52.81457944
SEC61A2	-1.402807855	0.00893708	0.040119627	4.937337961	4.834065788	1.514438878	1.418530939
TBC1D16	-1.404352925	3.96E-06	6.34E-05	19.19849506	31.72416088	8.011198744	7.231601252
KLHL22	-1.404749747	2.49E-05	0.000301403	20.92237119	32.0756654	8.957328821	6.877145745
SLC16A6	-1.409920456	0.000792225	0.005590514	14.5753727	41.47841107	6.749691974	10.06724531
OPRL1	-1.410095153	0.000713865	0.00516639	5.642560015	6.679464471	1.703664893	1.98565975
PRADC1	-1.410225212	5.46E-05	0.000595664	14.18358267	21.17902555	6.182013927	4.325066095
TMEM132A	-1.415880559	0.001904212	0.011416567	3.526893852	7.470349621	1.82981557	1.418530939
PCNT	-1.416084672	1.65E-05	0.000214861	23.5865434	38.49062272	9.651157545	8.791205482
DUSP14	-1.41833502	9.40E-05	0.000944252	7.836584184	12.21566052	4.100527756	1.772986445
SPSB2	-1.419604278	0.003271133	0.017874183	22.3328153	11.77627988	6.182013927	3.899719487
VEGFA	-1.421639299	0.000176177	0.0016081	13.71343464	32.16354152	6.308164604	7.302492353
S100A9	-1.422004815	2.84E-14	3.12E-12	394.611702	445.7964749	126.4036091	122.6423143
CABLES1	-1.422312273	0.003747111	0.019983205	10.10896636	4.218932894	2.397493617	1.843877547
CIDEB	-1.427106046	0.003226334	0.017709859	4.232115906	5.009818044	1.577514216	1.134966533
TBL1XR1	-1.429096695	8.23E-08	2.03E-06	48.19095729	50.26602384	11.60649304	17.58170205
PDLIM4	-1.431278888	0.003234182	0.017736401	3.056745816	9.227872177	1.82981557	1.772986445
STK36	-1.45403411	0.000242283	0.002097411	8.463448232	14.58831597	3.406699033	3.261699574
POC1A	-1.457254334	0.001677672	0.010319901	9.638818323	7.997606388	3.154397679	1.914768648
CHST15	-1.458484231	2.64E-07	5.79E-06	61.90360835	116.5246242	23.78003337	27.86091175
GK5	-1.458977856	0.00572013	0.028240576	7.836584184	2.988667105	1.577514216	1.560313141
GABBR1	-1.459399895	0.006776054	0.032197971	10.03060835	24.25469002	5.677411219	4.183283892
PSTPIP1	-1.461143212	3.26E-08	8.66E-07	44.351415	53.95682121	16.40021877	11.83952284
ORC1	-1.461853995	0.004499528	0.023198529	5.485844003	6.064331577	1.82981557	1.48942204
TSPAN15	-1.462073101	7.09E-12	4.49E-10	63.07897844	67.13824037	17.47249952	20.1337817

CTDSPL	-1.462295149	0.000320091	0.002662385	4.858979955	3.955304511	1.072911508	1.48942204
HAAO	-1.462801682	0.002029823	0.012050158	11.59776847	9.667252815	4.289753772	1.772986445
PRAM1	-1.46289196	2.70E-06	4.56E-05	19.35521107	14.93982048	4.79435648	5.10486821
EMILIN1	-1.463440266	2.72E-05	0.000326775	15.90745881	10.63389022	4.226678433	3.403481777
PNN	-1.464013617	2.77E-08	7.54E-07	60.02301621	72.85018868	17.2832735	21.05536602
IFT140	-1.467795967	0.001842736	0.01112546	6.582856087	9.403624432	2.649794971	1.914768648
P4HTM	-1.468651357	2.01E-05	0.000252757	21.94102527	28.2969919	8.326575436	6.026452528
MYO1B	-1.468806259	0.003122081	0.017268974	1.019437659	2.988667105	0.694459477	0.426055519
AATK	-1.471516166	1.49E-08	4.33E-07	64.09763252	84.09833303	21.50932118	20.91358382
IFT88	-1.472217699	0.001565327	0.009757551	6.817930106	7.821854132	2.397493617	1.772986445
C19orf35	-1.475065187	0.001387756	0.008884966	9.717176329	8.436987027	2.902096325	2.269224155
PLEKHH3	-1.477799826	0.003873552	0.020558746	7.444794154	6.415836088	2.839020986	1.064075432
RGCC	-1.479090546	2.84E-05	0.000336949	42.78425488	112.0429417	23.338506	20.70091051
HAGHL	-1.481377252	0.000131085	0.001251943	13.40000261	16.43371465	5.235883849	3.190808473
ARRDC4	-1.48576586	3.49E-10	1.53E-08	29.93354189	36.64522404	8.705027467	10.20902751
PITPNC1	-1.487427643	7.23E-06	0.000107116	19.43356908	41.82991558	8.011198744	9.358334293
GPR82	-1.489125755	7.87E-05	0.000819228	10.97090443	6.943092855	2.460568955	2.623679662
RNF125	-1.48919627	0.004309883	0.022469625	3.526893852	7.11884511	1.514438878	1.48942204
MICAL2	-1.491246538	2.78E-06	4.68E-05	13.00821258	20.47601653	4.163603095	5.317541514
CCNA2	-1.492185866	0.001624788	0.010045181	7.366436148	9.843005071	1.640589555	3.261699574
FEN1	-1.492229351	8.81E-08	2.16E-06	39.41486062	36.46947178	11.73264372	9.641898698
MRO	-1.492790815	1.03E-08	3.17E-07	41.45216878	48.15699678	10.91266432	14.46249359
DHRS13	-1.494406724	0.005345264	0.026663081	3.37017784	3.515923872	0.883685492	1.064075432
MXD3	-1.496229691	0.000719374	0.005200287	9.482102311	8.70061541	2.902096325	2.198333054
IFT22	-1.496863612	0.000481848	0.00373445	6.347782069	5.712827066	1.892890909	1.48942204
OTUD3	-1.500842598	0.004039432	0.021290372	8.620164244	6.679464471	1.766740232	2.552788561
ZSWIM4	-1.503773126	0.000208886	0.001853896	13.79179264	35.06345374	6.118938589	7.586056758

LMNB2	-1.505931875	1.45E-08	4.24E-07	35.96710835	52.0235464	10.91266432	13.75358258
GUSBP11	-1.506380596	1.56E-05	0.000205031	16.37760684	24.51831841	6.623541297	4.750412703
PC	-1.508264933	5.31E-05	0.000583098	17.55297693	44.20257103	7.632746713	9.641898698
ITGB2-AS1	-1.508995389	4.41E-05	0.0004966	11.12762044	11.86415601	4.289753772	2.056550851
SNHG3	-1.509923477	1.70E-08	4.86E-07	33.06786213	28.73637254	8.326575436	8.932987685
SGK494	-1.514395649	0.009721559	0.042955243	4.388831918	2.81291485	0.946760831	1.064075432
PTPRM	-1.515306083	0.000673344	0.004917523	5.877634033	18.27911334	3.028247002	3.687046183
NCAPD2	-1.517028644	6.73E-07	1.35E-05	29.69846787	49.47513869	10.09268491	11.91041394
SLC2A3	-1.517544546	5.28E-08	1.35E-06	65.82150865	121.7971918	27.438403	24.52902999
CHST2	-1.518222415	0.000778175	0.005531943	5.407485997	12.30353665	2.019041586	2.907244068
CD44	-1.522204043	9.50E-09	2.95E-07	369.6938561	717.7730903	138.5140741	162.6957866
IFT122	-1.525321077	7.06E-10	2.86E-08	47.87752527	43.14805749	11.22804101	13.96625588
PINK1	-1.526073099	1.04E-09	4.12E-08	80.47445578	117.9306422	28.76298511	25.87596091
CD163L1	-1.526499365	1.80E-09	6.68E-08	44.50813101	75.48647251	15.32793801	17.79437536
SLC22A5	-1.526639133	0.001541998	0.009633147	5.642560015	7.206721238	1.766740232	1.772986445
SWI5	-1.528095742	0.000223069	0.001958092	7.209720136	14.0610592	3.406699033	2.411006358
SIRPB1	-1.530176999	8.74E-08	2.15E-06	51.24691953	49.91451933	13.1833765	14.6751669
EXOSC5	-1.532766662	0.000143647	0.001354	9.717176329	11.16114699	3.09132234	2.623679662
NBPF14	-1.533804003	0.00021215	0.001875942	25.38877754	48.68425354	7.191219343	13.25734487
P2RY2	-1.533820122	0.008758298	0.039472518	5.015695967	7.558225749	2.145192263	1.276748736
TSPYL5	-1.535978173	0.000310132	0.002588628	9.011954275	10.19450958	2.902096325	2.340115256
PSPH	-1.538279253	1.42E-05	0.000189652	9.090312281	9.930881199	2.775945648	2.411006358
TBC1D30	-1.538574625	0.000146781	0.001376343	6.269424063	5.976455449	1.451363539	1.914768648
BMP1	-1.5415823	4.33E-05	0.000489256	7.053004124	11.5126515	2.902096325	2.127441952
CXXC5	-1.544657987	1.90E-08	5.38E-07	59.63122618	106.067365	22.07699923	23.04031686
FAM168B	-1.544962798	1.17E-06	2.19E-05	42.23574884	94.20408772	17.5986502	19.56665289
MSANTD3	-1.546096465	5.84E-06	8.92E-05	18.414915	43.23593362	7.506596036	9.287443192

NBPF10	-1.549261556	0.004832286	0.02458911	7.679868172	20.12451202	2.902096325	4.679521602
LINC00294	-1.550263249	2.15E-06	3.70E-05	13.40000261	19.59725525	4.605130464	4.325066095
C2orf74	-1.550497225	0.000502512	0.003871727	8.228374214	18.10336108	3.785151064	3.332590676
SH3YL1	-1.553917268	0.001765788	0.010751936	3.448535846	5.449198683	1.262137524	1.134966533
PTPN22	-1.554715258	0.000107188	0.001053697	4.623905936	7.11884511	0.946760831	2.269224155
ATG16L2	-1.556119773	4.26E-07	8.96E-06	59.63122618	84.0104569	24.78923879	13.75358258
PTGFRN	-1.558160788	9.23E-09	2.89E-07	27.89623373	42.44504847	9.525006868	9.429225394
LUCAT1	-1.562368997	0.001059979	0.007128756	7.679868172	13.53380244	3.53284971	2.127441952
LOC100288203	-1.56288392	0.0039072	0.020690526	6.7395721	7.997606388	1.640589555	2.340115256
DPCD	-1.567414968	3.32E-05	0.000386234	7.993300196	8.70061541	2.27134294	2.198333054
PEX6	-1.567968442	7.51E-05	0.000788222	14.88880473	23.81530939	5.992787912	4.325066095
ATP9B	-1.570006447	6.61E-07	1.33E-05	28.60145578	44.02681877	7.885048067	11.62684954
PCSK6	-1.580274958	2.18E-05	0.000271157	22.48953131	64.50195654	11.66956838	11.41417623
THNSL2	-1.580838547	0.001117164	0.007437889	7.209720136	10.80964248	2.839020986	1.914768648
SLC16A10	-1.582552126	0.000526244	0.004026628	4.467189924	10.28238571	1.451363539	2.481897459
RAD51	-1.58270012	0.002799435	0.015742865	6.896288112	4.746189661	1.199062185	1.914768648
SLC5A3	-1.589148275	2.16E-05	0.000268266	13.32164461	31.98778927	5.046657834	6.948036846
DDX12P	-1.589276249	0.001018956	0.006893275	6.426140075	9.052119921	1.892890909	2.198333054
AGPAT4	-1.595433024	2.53E-05	0.000306302	20.45222316	49.03575805	9.714232883	8.507641076
LINC01503	-1.597762227	7.10E-05	0.000753776	8.541806238	10.63389022	2.145192263	2.907244068
HIPK2	-1.598434576	1.02E-05	0.000144864	32.12756606	70.21390485	11.66956838	15.24229571
DANCR	-1.598906215	0.00083936	0.00584313	3.291819834	7.733978005	1.262137524	1.631204243
TMEM161B	-1.602487397	5.34E-05	0.000584414	6.896288112	6.943092855	1.514438878	2.127441952
TNNI2	-1.603352292	0.000337324	0.002780085	7.288078142	5.712827066	1.766740232	1.631204243
ANKH	-1.603447914	1.34E-09	5.12E-08	144.1795147	256.1597912	43.20723763	61.81774931
FBP1	-1.606327823	2.30E-08	6.33E-07	30.87383796	48.2448729	11.35419168	9.21655209
BAIAP2-AS1	-1.607667106	4.92E-11	2.57E-09	30.40368992	38.05124208	8.831178144	9.003878786

CDKN2B	-1.610574784	0.000423504	0.003354331	6.269424063	11.86415601	2.902096325	1.772986445
NSUN5P1	-1.612225502	7.59E-05	0.000795134	8.855238263	8.524863154	2.712870309	1.772986445
EHD1	-1.619261448	3.01E-14	3.29E-12	78.67222164	94.46771611	22.96005397	21.76427703
CCDC125	-1.619653736	0.000205027	0.001827512	5.329127991	7.11884511	1.451363539	1.772986445
TLR5	-1.621594883	0.000214099	0.00188855	8.698522251	8.964243793	1.955966247	2.623679662
IRGQ	-1.624295998	1.13E-10	5.49E-09	39.72829264	59.05363662	11.73264372	13.75358258
MIB2	-1.624741892	4.13E-06	6.56E-05	31.81413403	46.57522648	11.66956838	8.436749975
TRIM16	-1.626658281	8.00E-06	0.000116609	13.32164461	17.48822819	3.280548356	4.679521602
UPK3BL1	-1.630172386	0.000144207	0.001358384	49.6014014	35.85433889	15.89561606	5.813779224
SLC43A1	-1.633855284	1.44E-05	0.00019147	11.75448449	15.29132499	3.911301741	2.978135169
ADK	-1.636478586	4.22E-06	6.68E-05	18.80670503	44.20257103	7.191219343	8.932987685
IL16	-1.638084291	9.75E-12	5.99E-10	37.9260585	36.55734791	8.705027467	10.35080971
DLG5	-1.639120708	0.004067552	0.021421402	3.526893852	6.943092855	1.451363539	1.205857634
NBPF19	-1.641971899	3.62E-06	5.86E-05	47.79916726	88.05275878	16.58944478	18.00704866
PLXNA1	-1.644492108	3.95E-10	1.72E-08	47.09394521	49.65089095	14.12950658	10.35080971
TTLL4	-1.644716085	1.82E-11	1.06E-09	65.27300261	86.11948397	17.40942418	21.12625712
GADD45A	-1.646731916	0.000295688	0.002490237	4.075399894	5.624950938	1.577514216	0.851402128
MCM4	-1.652036702	1.56E-10	7.41E-09	34.63502225	29.00000092	6.938917989	9.21655209
WDR54	-1.657008518	0.0012363	0.008076524	8.620164244	7.030968982	1.892890909	2.056550851
ANKRD42	-1.657896243	0.000785503	0.005567579	6.974646118	20.73964491	3.154397679	3.828828385
FN1	-1.658463749	1.52E-09	5.73E-08	1622.873447	3166.617143	548.5668497	658.791714
GLIS3	-1.660190597	0.0072378	0.033889909	3.840325876	5.53707481	0.883685492	1.48942204
CDR2L	-1.663031936	0.000924592	0.006344643	5.955992039	6.064331577	1.325212862	1.702095344
MSC	-1.665872572	1.85E-05	0.000236522	34.32159023	97.98276122	17.53557486	15.52586011
TMEM198B	-1.667050697	1.60E-05	0.000208968	9.795534335	14.93982048	2.460568955	3.757937284
FAM212B	-1.66706713	1.65E-06	2.93E-05	15.90745881	21.44265394	4.857431818	4.466848298
TEN1-CDK3	-1.667576195	4.58E-05	0.000511922	11.44105246	19.06999849	3.659000387	3.970610588

PIM1	-1.668124791	1.21E-05	0.000165516	29.77682588	71.26841838	13.43567786	11.76863174
TFPI	-1.668761383	8.80E-06	0.000126375	16.69103887	35.15132987	5.677411219	7.302492353
DNAH1	-1.673050069	0.000112989	0.00110172	10.03060835	14.93982048	3.028247002	3.190808473
CD101	-1.68051218	1.08E-05	0.000151782	11.75448449	16.43371465	2.902096325	4.112392791
ADCY6	-1.682581618	0.000356054	0.00291117	5.720918021	3.428047744	1.135986847	1.134966533
MRC1	-1.682812436	3.76E-07	8.00E-06	93.48188479	210.2005764	28.5737591	46.93061802
CBX5	-1.689594811	2.72E-10	1.25E-08	26.01564159	25.48495581	5.235883849	7.515165657
ZNF584	-1.691570478	0.000826559	0.005767925	4.623905936	7.90973026	1.451363539	1.631204243
PDE4DIP	-1.694756183	0.004478216	0.023121693	79.61251772	291.0466139	41.75650485	49.34091547
PPARD	-1.701381783	1.27E-09	4.87E-08	30.63876394	49.29938644	8.515801452	11.05972073
PSMC3IP	-1.708906838	0.008642435	0.039035676	2.900029804	3.955304511	0.946760831	0.709619925
IRS2	-1.714590206	0.000895799	0.006176432	6.661214093	18.89424623	2.586719632	3.616155081
YPEL3	-1.716287867	3.02E-08	8.07E-07	34.16487421	61.33841594	11.92186973	11.13061183
LOC284454	-1.718507332	9.67E-05	0.000967079	11.9112005	34.79982536	5.929712573	5.317541514
SLC39A8	-1.719733741	7.47E-10	3.01E-08	176.3062972	357.3052143	58.7237709	70.18289928
TLE3	-1.727583385	3.24E-06	5.31E-05	32.6760721	77.68337571	12.04802041	14.46249359
TONSL	-1.729392297	2.46E-05	0.000299338	10.97090443	8.964243793	1.703664893	3.119917372
PLEKHF1	-1.729694388	0.003090745	0.017121898	1.489585695	2.461410339	0.252932107	0.709619925
KLHL17	-1.731542284	8.88E-05	0.000898978	10.73583041	10.72176635	2.712870309	2.411006358
SMAGP	-1.732610757	0.007441223	0.034673648	1.959733731	3.6038	0.883685492	0.426055519
GAS5	-1.737267955	5.85E-08	1.48E-06	38.00441651	57.55974245	12.42647244	10.27991861
IL7R	-1.737332031	0.003442853	0.018597019	2.97838781	7.733978005	1.135986847	1.418530939
LOC100506100	-1.737730509	0.005745166	0.028312638	3.840325876	4.834065788	1.135986847	0.922293229
EMC3-AS1	-1.742011615	0.007656468	0.035368276	3.213461828	4.131056766	0.757534815	0.99318433
PVT1	-1.743513584	0.005025567	0.025395607	1.803017719	3.6038	0.442158123	0.851402128
PLIN2	-1.74922165	7.77E-07	1.53E-05	271.8247065	790.0072674	137.189492	113.2137978
TIAM1	-1.753984012	1.59E-05	0.000208217	6.896288112	17.92760883	2.523644294	3.332590676

SLC25A29	-1.755930592	1.25E-06	2.33E-05	20.13879113	42.35717234	7.380445359	7.302492353
UNC119B	-1.756654304	2.52E-07	5.58E-06	14.81044672	21.70628232	4.920507157	3.616155081
GPC1	-1.759848466	0.000783923	0.005561004	2.116449743	2.725038722	0.694459477	0.426055519
TPX2	-1.760456095	1.68E-06	2.98E-05	17.1611869	17.2245998	3.217473017	4.892194906
CDCA4	-1.761107292	8.26E-05	0.000851335	12.92985458	23.20017649	2.523644294	6.026452528
SH3BP5-AS1	-1.767151468	5.75E-08	1.46E-06	21.78430926	21.61840619	4.289753772	5.884670325
MAPRE3	-1.769048911	4.91E-09	1.66E-07	24.44848146	34.71194923	6.49739062	7.302492353
ITGA11	-1.779828281	0.001709156	0.010470446	4.232115906	2.988667105	0.757534815	0.922293229
SHCBP1	-1.780593915	1.30E-05	0.000175744	14.18358267	13.53380244	3.217473017	3.190808473
SMAD6	-1.780781542	0.001717149	0.010500502	1.959733731	2.988667105	0.442158123	0.709619925
PNP	-1.784423675	7.67E-10	3.08E-08	74.67596334	146.2267554	25.67229353	25.237941
AK4	-1.785230305	0.00595146	0.029163162	1.332869683	10.63389022	1.199062185	1.560313141
NRAV	-1.786218938	0.001234548	0.008072387	3.605251858	4.834065788	0.883685492	1.064075432
CD93	-1.786807473	3.00E-15	3.76E-13	203.1830932	248.7781965	41.81958018	62.66844253
SESN3	-1.788656929	1.59E-05	0.000208217	6.034350045	7.997606388	1.262137524	1.98565975
ABCA2	-1.78918194	3.30E-05	0.000384586	6.112708051	9.227872177	2.145192263	1.347639837
HIP1	-1.790273287	0.000111915	0.001092721	6.191066057	16.87309529	2.460568955	2.836352966
PLPP4	-1.790916462	0.000145633	0.001366912	5.329127991	7.206721238	1.388288201	1.48942204
TCF19	-1.790978018	4.44E-09	1.51E-07	22.3328153	20.91539717	5.298959188	4.608630501
BLM	-1.79133824	0.002578199	0.014740886	5.250769985	4.306809022	1.262137524	0.922293229
TLN2	-1.793114304	0.005095723	0.025685079	2.194807749	1.846277444	0.694459477	0.213382215
PYROXD2	-1.79370014	0.009109245	0.040710904	2.743313791	2.549286466	0.379082784	0.851402128
FOXM1	-1.79384871	6.29E-08	1.58E-06	21.0790872	20.56389266	3.785151064	5.813779224
TNFRSF12A	-1.796906047	1.04E-05	0.000146576	7.053004124	19.421503	3.659000387	2.340115256
NT5DC2	-1.797543264	1.80E-12	1.30E-10	126.8623954	202.2038487	41.94573086	33.03596215
MICALL2	-1.798530825	7.98E-07	1.56E-05	16.37760684	30.58177122	5.929712573	4.750412703
WDHD1	-1.798963507	0.000376364	0.003044459	6.347782069	7.11884511	1.514438878	1.560313141

MAD2L1	-1.799175343	0.000613974	0.00455775	6.034350045	6.064331577	0.946760831	1.843877547
SH3BP5	-1.801614988	1.13E-09	4.43E-08	33.06786213	59.75664564	9.02040416	12.19397835
ALDH7A1	-1.802176914	1.24E-06	2.30E-05	8.071658202	7.206721238	1.514438878	1.98565975
CLCF1	-1.80221368	0.001397526	0.008931662	5.172411979	11.5126515	2.082116924	1.702095344
TANC2	-1.812118196	7.01E-07	1.39E-05	12.61642255	23.28805262	3.53284971	4.608630501
WDR62	-1.813754909	1.80E-05	0.0002317	9.873892341	12.21566052	2.839020986	2.127441952
SIGLEC10	-1.815294197	4.55E-10	1.93E-08	22.09774128	31.63628476	6.623541297	5.459323717
SRGAP1	-1.818109674	0.005162334	0.025935303	1.803017719	1.758401317	0.379082784	0.426055519
ORC6	-1.826097722	0.0008849	0.006107125	9.090312281	4.39468515	1.135986847	1.914768648
ABCA5	-1.826724453	0.000603408	0.004499746	3.135103822	4.043180638	0.757534815	0.851402128
ME3	-1.828011723	9.93E-05	0.00098961	4.858979955	8.788491538	2.019041586	0.99318433
RASSF2	-1.83018474	2.12E-07	4.76E-06	59.39615216	151.1478185	18.79708163	28.42804056
SKA3	-1.834479358	0.006110975	0.029783004	4.232115906	3.340171616	0.505233461	1.205857634
ARAP3	-1.838347895	4.20E-05	0.000476087	3.605251858	8.085482516	1.703664893	0.851402128
PTPRCAP	-1.839472596	0.000981179	0.006694751	6.504498081	7.206721238	2.019041586	0.99318433
FAM86DP	-1.841150228	3.79E-05	0.000433776	5.172411979	8.436987027	1.388288201	1.631204243
SLX1B-SULT1A4	-1.847339012	3.82E-06	6.13E-05	14.02686666	17.57610431	4.100527756	2.836352966
IQGAP3	-1.848127974	1.16E-05	0.000159667	8.855238263	12.30353665	1.325212862	3.403481777
LOC103344931	-1.849898253	0.007107088	0.033418853	1.803017719	3.691676127	0.505233461	0.709619925
STIL	-1.85570058	0.001382934	0.008869831	4.780621949	4.043180638	0.946760831	0.99318433
CDCA8	-1.860032416	8.43E-05	0.000863712	7.758226178	8.436987027	1.703664893	1.843877547
SGPP2	-1.860156971	0.00014262	0.001347835	9.403744305	20.03663589	2.839020986	3.616155081
AQP9	-1.860219147	8.39E-05	0.000861928	25.62385156	93.5010787	12.80492447	13.25734487
TMEM80	-1.860826068	1.81E-06	3.18E-05	13.00821258	18.8063701	3.343623694	3.616155081
SDC1	-1.861225711	0.008340015	0.037966799	0.706005634	1.143268422	0.379082784	0.000708911
MACC1	-1.862662873	0.000820884	0.005736645	7.52315216	9.579376688	1.955966247	1.772986445
NT5C3B	-1.863615999	0.000188589	0.001705214	16.06417482	73.90470221	11.60649304	7.940512265

MIR5047	-1.864695033	6.49E-05	0.000697548	6.817930106	6.943092855	1.577514216	1.418530939
CDK1	-1.866917568	1.99E-05	0.000250007	13.40000261	10.98539473	2.019041586	3.332590676
SLC16A7	-1.867266106	0.000571256	0.004289175	1.959733731	2.637162594	0.694459477	0.284273317
ZNF589	-1.867708644	3.14E-10	1.40E-08	16.06417482	16.96097142	4.100527756	3.04902627
NCAPG2	-1.868447411	6.77E-05	0.000723485	10.50075639	12.4792889	1.577514216	3.474372879
LAT	-1.870026083	7.78E-06	0.000113933	11.59776847	39.72088851	5.740486558	5.388432616
MIR3064	-1.871199114	9.03E-05	0.000911433	5.720918021	6.591588344	1.325212862	1.347639837
CACNA2D4	-1.872292004	4.83E-12	3.09E-10	44.89992104	62.4808056	13.81412989	9.358334293
TBXAS1	-1.877745164	9.89E-18	2.23E-15	124.3549392	166.9655215	27.7537797	35.3753685
PLK4	-1.881420803	0.001313735	0.008516852	6.191066057	4.39468515	0.883685492	1.418530939
NCAPG	-1.882199113	0.000350532	0.002875798	10.6574724	9.052119921	1.451363539	2.836352966
BRICD5	-1.888370247	0.003152425	0.017396806	3.918683882	5.712827066	1.135986847	0.922293229
DYRK3	-1.888761257	0.000578885	0.004337429	1.489585695	2.900790978	0.505233461	0.426055519
AMPD3	-1.893525896	1.53E-16	2.39E-14	82.90355397	113.4489597	18.48170494	23.60744567
VAMP1	-1.894153413	2.57E-06	4.37E-05	9.795534335	9.755128943	2.649794971	1.48942204
UBXN11	-1.894380483	3.91E-10	1.71E-08	51.87378358	82.07718209	17.53557486	10.91793852
LCAT	-1.895689625	0.002723658	0.015401686	4.937337961	7.11884511	1.82981557	0.709619925
AMPD2	-1.896945255	1.37E-17	2.90E-15	66.91852074	79.96815503	16.52636944	14.746058
CASTOR1	-1.899265657	0.000304857	0.002553329	5.250769985	11.86415601	2.397493617	1.205857634
CHTF18	-1.902826966	1.64E-06	2.91E-05	15.04552074	12.12778439	3.469774371	2.269224155
SPOCD1	-1.907555106	0.000244137	0.002109664	3.37017784	14.50043984	1.325212862	2.481897459
SAPCD2	-1.90837373	0.000586512	0.004385468	5.329127991	9.139996049	1.766740232	1.276748736
USP13	-1.910140366	0.002288072	0.013346471	2.97838781	6.503712216	1.072911508	0.922293229
ANLN	-1.911707037	0.000833261	0.005806274	8.071658202	7.206721238	1.262137524	1.98565975
TLE1	-1.913020243	0.008007459	0.036707636	0.706005634	3.779552255	0.379082784	0.567837722
SKA1	-1.914517989	0.002712098	0.015348348	5.094053973	4.131056766	0.820610154	1.134966533
JAML	-1.918399742	0.001851504	0.011160841	4.1537579	3.076543233	1.072911508	0.426055519

CXCR4	-1.919400684	4.30E-07	9.01E-06	82.82519596	223.9092523	35.07051896	29.20784268
PCED1B	-1.923735589	0.001008484	0.006832594	3.605251858	4.39468515	0.757534815	0.922293229
LY6K	-1.92429484	0.004776933	0.024350378	5.720918021	15.90645789	2.523644294	1.98565975
ZNF704	-1.927914376	0.000791718	0.005590514	3.213461828	6.767340599	0.883685492	1.205857634
CLLU1OS	-1.928985902	0.000206596	0.001839294	9.482102311	29.61513382	4.037452418	4.112392791
SLC7A11	-1.929476087	1.32E-07	3.08E-06	90.73935457	223.9092523	27.81685503	37.99833925
CRIPAK	-1.929795555	3.45E-07	7.42E-06	14.65373071	16.96097142	3.659000387	2.907244068
TBC1D17	-1.929832447	7.18E-12	4.51E-10	31.03055397	46.31159809	7.695822051	8.436749975
ZWINT	-1.931344261	1.12E-10	5.47E-09	20.21714914	22.40929134	4.542055126	4.325066095
CLEC10A	-1.937235535	0.000113351	0.0011045	11.83284249	26.36371709	3.406699033	4.537739399
TMEM97	-1.940051816	8.56E-06	0.000123342	9.168670287	8.788491538	2.27134294	1.418530939
SERINC2	-1.940284841	9.63E-07	1.85E-05	31.18726998	90.60116649	12.23724642	12.97378046
MT1E	-1.940619408	2.93E-05	0.000346725	16.29924884	21.26690168	5.488185203	2.198333054
TMPO-AS1	-1.944065249	0.008594106	0.038878225	2.821671798	2.373534211	0.442158123	0.638728823
NOL4L	-1.945811465	0.000639608	0.004704474	2.429881767	5.976455449	1.072911508	0.638728823
CTTN	-1.945967875	3.55E-08	9.37E-07	9.090312281	22.40929134	3.217473017	3.261699574
FAM102A	-1.946198662	5.32E-07	1.10E-05	4.467189924	7.821854132	1.703664893	0.780511026
RAB11FIP4	-1.94752276	0.000430663	0.003394266	5.015695967	11.86415601	1.766740232	1.702095344
SH3PXD2A	-1.94790291	1.34E-06	2.47E-05	12.38134853	37.61186144	5.172808511	5.10486821
MMP9	-1.951903809	3.25E-17	6.17E-15	4480.903359	4875.104819	1147.341038	766.1208415
AMT	-1.953744974	0.000988172	0.006726558	6.896288112	11.33689924	2.965171663	0.709619925
RNASEH1-AS1	-1.959693015	0.002615023	0.014904187	3.918683882	3.6038	0.757534815	0.780511026
CHI3L1	-1.966543483	1.24E-08	3.73E-07	201.9293651	72.85018868	29.83526587	25.94685202
GOLGA8B	-1.966934996	0.00093452	0.006400595	2.664955785	7.206721238	1.072911508	0.922293229
NME1-NME2	-1.966996203	0.000197312	0.001771882	5.799276027	4.570437405	1.640589555	0.426055519
CMTM4	-1.968232347	1.41E-05	0.000188327	6.661214093	10.98539473	2.334418278	1.205857634
THBD	-1.974228382	1.58E-14	1.79E-12	124.9034452	210.1127002	36.26895039	31.33457572

SOX13	-1.976471676	1.57E-07	3.62E-06	5.094053973	5.888579322	0.757534815	1.48942204
RUBCNL	-1.978662358	7.44E-08	1.86E-06	10.97090443	15.46707725	3.028247002	2.269224155
KIF11	-1.98359714	0.000730176	0.005260693	10.89254642	10.63389022	1.388288201	2.978135169
A1BG	-1.98714701	4.23E-05	0.000479934	8.071658202	17.04884755	3.280548356	1.702095344
NPIPA2	-1.988902978	0.00816853	0.037362933	3.76196787	2.988667105	0.379082784	0.99318433
CCNB2	-1.99046011	5.39E-06	8.32E-05	11.20597844	10.54601409	2.019041586	2.340115256
NCAPH	-1.99072971	2.98E-06	4.96E-05	10.10896636	10.01875733	1.703664893	2.340115256
LOC105372480	-1.997952898	0.00550707	0.027330121	3.683609864	3.955304511	1.072911508	0.426055519
SERPINB2	-1.999675523	0.00165722	0.010215867	236.4852458	862.5929489	96.19052198	122.6423143
PLPP3	-2.000143396	1.36E-06	2.48E-05	24.44848146	52.19929865	6.812767312	8.436749975
GINS2	-2.002513311	0.000224268	0.001966227	9.560460317	4.746189661	1.955966247	0.851402128
MCM7	-2.003226586	3.76E-18	9.04E-16	61.98196636	63.79894752	12.17417109	12.76110716
MAPK8IP3	-2.00536955	5.70E-14	5.71E-12	53.28422769	81.81355371	13.1833765	13.54090927
ZNF367	-2.005450695	0.007059654	0.033293421	3.291819834	1.758401317	0.505233461	0.496946621
SLC12A6	-2.00550721	5.22E-11	2.71E-09	26.95593766	39.72088851	7.191219343	5.955561427
CKAP2L	-2.006301823	0.007345013	0.034302874	4.467189924	4.658313533	0.883685492	0.922293229
NQO2	-2.008560784	3.78E-16	5.56E-14	161.496634	116.3488719	27.62762902	27.22289184
INSR	-2.011255955	4.26E-06	6.71E-05	10.10896636	19.86088364	2.586719632	3.332590676
STMN1	-2.016508934	7.53E-14	7.39E-12	47.17230322	50.35389997	7.75889739	11.48506733
SIGLEC7	-2.019539362	3.76E-15	4.63E-13	67.38866877	48.50850129	11.10189033	11.62684954
CELF6	-2.021189599	2.29E-05	0.000281432	6.974646118	7.733978005	1.325212862	1.560313141
ASMTL-AS1	-2.022774627	0.00017201	0.001578052	5.485844003	11.5126515	2.019041586	1.276748736
DDIT4	-2.02409872	3.71E-13	3.19E-11	81.49310986	117.3155093	18.98630764	19.8502173
OIP5	-2.028224684	0.005001719	0.025299618	3.291819834	4.306809022	0.5683088	0.922293229
ACP6	-2.03352051	0.001422623	0.009061558	3.448535846	7.470349621	1.451363539	0.638728823
FABP3	-2.035245004	2.04E-07	4.61E-06	7.679868172	17.2245998	2.27134294	2.552788561
DST	-2.037638204	2.72E-06	4.60E-05	9.011954275	13.88530695	1.892890909	2.552788561

LOC441081	-2.040127736	5.42E-07	1.11E-05	20.60893917	20.21238815	3.848226402	4.04150169
NUP210	-2.040783186	2.04E-08	5.72E-07	16.61268086	23.02442424	4.35282911	3.261699574
EDNRB	-2.041309385	0.00066171	0.004835009	9.325386299	16.08221014	1.072911508	3.899719487
PGBD5	-2.052350189	0.001017035	0.00688407	2.97838781	7.733978005	1.451363539	0.567837722
BHLHE41	-2.056909749	0.000324141	0.002689871	7.758226178	13.18229793	1.135986847	2.907244068
LTBP2	-2.057682008	5.46E-07	1.11E-05	13.87015065	41.65416332	4.79435648	5.813779224
CDC25A	-2.066585021	0.000295168	0.00248807	4.075399894	5.449198683	0.883685492	0.922293229
SEC31B	-2.067026383	1.77E-05	0.000227696	7.52315216	11.33689924	2.397493617	1.134966533
MYC	-2.068701191	7.06E-06	0.000105034	20.60893917	72.93806481	11.41726702	6.168234731
UNC5B	-2.071065136	0.00111741	0.007437889	1.646301707	3.691676127	0.442158123	0.567837722
ICAM5	-2.073188228	0.00097717	0.006670548	4.310473912	4.834065788	1.072911508	0.638728823
ZNF587B	-2.074929276	0.000332459	0.002746863	3.37017784	5.888579322	0.757534815	0.99318433
PAPLN	-2.080294214	4.08E-07	8.65E-06	11.44105246	17.66398044	2.965171663	2.481897459
DEPTOR	-2.081674609	0.000250141	0.002149962	4.388831918	3.340171616	1.135986847	0.284273317
RFLNB	-2.081716394	1.23E-09	4.74E-08	9.482102311	14.41256371	2.208267601	2.269224155
SH3PXD2B	-2.084073922	0.004991612	0.025257346	12.22463252	51.23266125	4.163603095	7.798730063
LRRC37A2	-2.088890714	0.002542819	0.01460771	4.232115906	11.68840375	1.009836169	1.98565975
ITGAE	-2.089867499	3.85E-12	2.52E-10	41.29545276	53.69319283	10.47113695	7.16071015
ANKDD1A	-2.090805326	1.96E-05	0.000247791	9.795534335	10.80964248	2.460568955	1.347639837
ZNF395	-2.103501262	4.02E-08	1.05E-06	19.12013705	43.76319039	5.866637235	5.742888123
PHACTR1	-2.103527279	0.007487272	0.034809541	1.724659713	3.691676127	0.5683088	0.426055519
ALOX5	-2.104807478	1.92E-08	5.43E-07	45.29171107	131.0241853	14.88641064	17.72348426
KYAT1	-2.109320424	0.000241197	0.00209052	4.623905936	3.340171616	0.757534815	0.709619925
IFT172	-2.110562657	2.11E-06	3.66E-05	8.385090226	6.855216727	1.388288201	1.418530939
ASF1B	-2.110863062	6.35E-09	2.08E-07	17.55297693	15.20344886	2.712870309	3.332590676
ASGR1	-2.11488573	0.000359747	0.00293637	9.168670287	12.30353665	2.019041586	1.914768648
KHK	-2.115041051	2.61E-05	0.000314046	9.403744305	7.382473493	1.388288201	1.702095344

ZNF697	-2.11991534	1.72E-05	0.000222683	5.720918021	14.0610592	1.766740232	1.843877547
LOC155060	-2.122096895	0.006575564	0.031473287	1.959733731	5.449198683	0.442158123	0.922293229
SSBP3	-2.125327747	6.01E-12	3.82E-10	34.71338026	66.2594791	9.02040416	9.358334293
SNHG14	-2.125591968	0.005268152	0.026379148	3.135103822	1.934153572	0.694459477	0.213382215
STEAP3	-2.131675133	4.35E-17	8.08E-15	47.32901923	72.58656029	10.47113695	11.27239403
CENPA	-2.134092813	0.006320244	0.030585997	2.351523761	3.515923872	0.694459477	0.355164418
PLXNA2	-2.138761412	4.67E-08	1.21E-06	8.855238263	15.37920112	3.028247002	1.276748736
SIRPB2	-2.139313244	0.00250865	0.0144405	9.560460317	2.461410339	0.505233461	1.702095344
CBFA2T3	-2.139811216	0.002578062	0.014740886	2.900029804	2.988667105	0.694459477	0.355164418
KIF2C	-2.142652859	1.35E-06	2.47E-05	8.541806238	10.01875733	1.703664893	1.631204243
ABCC3	-2.152611245	1.08E-12	8.26E-11	52.1872156	99.8281599	14.88641064	12.19397835
CR1	-2.154503564	0.0086216	0.03896596	1.803017719	5.712827066	0.631384138	0.709619925
CCNB1	-2.154519006	4.16E-09	1.43E-07	14.65373071	20.65176879	2.839020986	3.474372879
ATP2A3	-2.167694677	4.60E-08	1.19E-06	47.95588328	153.3447217	17.85095155	17.72348426
MCM2	-2.167816277	4.41E-14	4.57E-12	27.89623373	26.7152216	4.100527756	5.60110592
S100B	-2.178247795	2.94E-06	4.90E-05	12.06791651	5.361322555	1.640589555	1.418530939
SYT1	-2.179615492	0.000286435	0.002424369	2.97838781	6.855216727	1.199062185	0.496946621
PDE2A	-2.186772486	0.005932679	0.029081009	1.724659713	2.461410339	0.505233461	0.213382215
HSPA7	-2.190620902	4.15E-19	1.20E-16	101.3960434	89.98603359	19.68013637	13.47001817
WDR90	-2.195337823	2.94E-06	4.90E-05	10.34404038	13.79743082	1.325212862	2.907244068
PID1	-2.196044276	4.17E-15	5.06E-13	94.9706869	169.7775576	21.76162254	24.17457448
TROAP	-2.198167581	6.60E-05	0.000707574	6.426140075	7.030968982	1.325212862	0.99318433
VCAN	-2.199167578	0.000258705	0.002210404	6.974646118	21.17902555	1.577514216	3.332590676
RGS14	-2.203203749	2.28E-08	6.27E-07	26.87757965	17.2245998	3.785151064	3.828828385
XYLB	-2.209378764	0.009965784	0.043806343	0.706005634	2.81291485	0.316007446	0.284273317
ABCB5	-2.218160793	0.001061202	0.007129515	2.194807749	10.19450958	1.514438878	0.567837722
GAPLINC	-2.218580767	7.47E-08	1.86E-06	4.937337961	5.624950938	1.009836169	0.780511026

NTSR1	-2.219996451	1.34E-06	2.47E-05	5.094053973	10.54601409	1.577514216	1.064075432
TMEM163	-2.222159923	0.008268818	0.037749861	2.586597779	10.10663345	0.757534815	1.418530939
PPM1H	-2.228816069	3.94E-07	8.37E-06	5.720918021	12.12778439	1.262137524	1.772986445
GEM	-2.229292465	0.006385504	0.030812823	0.706005634	5.273446427	0.379082784	0.638728823
LINC00094	-2.233619286	1.09E-07	2.61E-06	9.011954275	12.03990827	1.514438878	2.056550851
CELSR1	-2.234548992	4.08E-10	1.75E-08	24.99698751	55.27496313	5.992787912	7.586056758
IGIP	-2.239359276	0.002989673	0.016638632	2.194807749	2.988667105	0.379082784	0.496946621
SEMA4B	-2.251743238	7.50E-08	1.87E-06	25.62385156	77.59549958	7.001993328	10.27991861
GOLGA8A	-2.255086834	2.73E-06	4.61E-05	6.347782069	17.40035206	1.892890909	2.056550851
FAM13A-AS1	-2.268949764	1.03E-05	0.000145047	8.150016208	7.997606388	1.514438878	1.134966533
BRCA1	-2.274087	6.96E-06	0.000103797	8.385090226	9.755128943	0.757534815	2.269224155
LRP5L	-2.274931417	0.000103542	0.001024139	5.877634033	5.888579322	1.072911508	0.851402128
MET	-2.279145331	0.001594334	0.00989584	1.489585695	4.306809022	0.252932107	0.709619925
TK1	-2.285118393	4.91E-14	4.95E-12	51.01184551	36.99672855	7.254294682	7.089819049
ST6GALNAC4	-2.287425312	5.75E-13	4.77E-11	19.19849506	35.85433889	4.478979787	4.466848298
TMEM119	-2.288020737	6.54E-23	4.11E-20	56.41854793	70.3896571	11.60649304	8.932987685
PLAU	-2.289053261	2.30E-11	1.30E-09	115.4221265	317.8488329	38.09813521	32.18526894
NIPAL4	-2.290046991	0.000817883	0.00572399	1.724659713	2.81291485	0.379082784	0.355164418
DGAT2	-2.291466676	0.004913319	0.024927871	2.821671798	5.273446427	1.072911508	0.213382215
MREG	-2.29312638	2.71E-08	7.39E-07	6.7395721	15.29132499	1.325212862	2.269224155
PRC1	-2.294231105	4.84E-11	2.54E-09	19.6686431	28.64849641	3.785151064	4.04150169
ATP8B3	-2.302000617	0.005555983	0.027519572	2.194807749	4.482561277	0.442158123	0.638728823
EHD3	-2.307770681	0.005738561	0.028312198	0.627647628	1.494772933	0.000630753	0.355164418
DTL	-2.315548201	0.000129957	0.001243638	5.407485997	2.988667105	0.505233461	0.851402128
SMG1P7	-2.325595206	0.002296079	0.013382357	2.900029804	5.097694172	0.694459477	0.567837722
MATK	-2.326133657	1.45E-15	1.94E-13	17.70969295	26.18796484	3.469774371	3.474372879
SRPX2	-2.32929003	0.007950407	0.036527324	0.78436364	5.097694172	0.631384138	0.284273317

SCD5	-2.338977465	0.004506396	0.023225644	1.019437659	2.461410339	0.000630753	0.567837722
REXO5	-2.3660901	0.001788194	0.010860867	3.840325876	1.758401317	0.505233461	0.355164418
SLC45A3	-2.369115351	3.80E-06	6.10E-05	5.015695967	9.579376688	0.379082784	1.914768648
MCM10	-2.370826804	0.002038291	0.012091732	4.623905936	2.373534211	0.379082784	0.709619925
SNHG10	-2.377129637	0.008710116	0.039292179	1.724659713	1.758401317	0.316007446	0.213382215
GPRIN3	-2.38016543	2.14E-06	3.70E-05	9.560460317	8.436987027	1.199062185	1.560313141
KCNMB4	-2.380844741	0.00251405	0.014465813	2.743313791	2.988667105	0.316007446	0.567837722
DDX11	-2.382621035	2.00E-08	5.62E-07	18.10148298	11.5126515	1.514438878	3.04902627
PHLDA3	-2.393061095	7.56E-08	1.87E-06	3.605251858	6.591588344	0.820610154	0.709619925
CDCA5	-2.397536073	3.29E-10	1.46E-08	12.77313856	14.32468759	1.82981557	2.269224155
MZF1	-2.408304542	4.36E-09	1.48E-07	13.08657059	23.551681	2.712870309	2.765461865
PLK1	-2.409104693	4.29E-09	1.47E-07	15.98581681	33.0423028	2.902096325	4.466848298
POLQ	-2.415003971	0.001837264	0.011102829	4.310473912	5.185570299	0.505233461	0.922293229
SFXN2	-2.420520988	0.009839646	0.043370845	1.254511677	0.967516167	0.316007446	0.000708911
MT1F	-2.424500769	1.57E-05	0.000206761	10.50075639	43.85106652	5.551260542	2.411006358
LOC105369340	-2.427465843	0.00520615	0.026113989	0.862721647	2.81291485	0.12678143	0.426055519
THBS1	-2.434140364	1.91E-12	1.37E-10	196.2875887	464.5140901	47.87481268	49.27002437
LOC101929331	-2.434859229	0.004443694	0.023001056	2.97838781	1.582649061	0.252932107	0.426055519
HMCN1	-2.43692105	0.008947606	0.040125202	2.116449743	4.746189661	0.442158123	0.567837722
MERTK	-2.441244576	2.38E-17	4.74E-15	161.496634	329.7121101	32.35827941	39.62883458
FRY	-2.446636225	0.000606642	0.004514934	1.881375725	6.855216727	0.631384138	0.638728823
ESPL1	-2.447451863	1.43E-07	3.32E-06	8.620164244	10.63389022	1.451363539	1.347639837
MST1	-2.449070873	8.08E-05	0.000836722	4.780621949	5.273446427	0.883685492	0.567837722
SETD9	-2.458860453	0.00914912	0.040838526	1.803017719	2.461410339	0.000630753	0.638728823
S100A8	-2.463796219	4.83E-12	3.09E-10	18.80670503	36.29371953	4.289753772	3.616155081
FGFR1	-2.472243061	3.52E-11	1.90E-09	12.53806455	18.8063701	2.082116924	2.411006358
MMP2-AS1	-2.474189015	0.001894615	0.011373209	2.038091737	4.482561277	0.5683088	0.355164418

ENO3	-2.482615292	9.03E-07	1.74E-05	7.758226178	11.95203214	1.514438878	1.276748736
KIF20A	-2.492203641	0.001277808	0.00832884	4.702263942	6.240083833	0.631384138	0.922293229
LHX4-AS1	-2.507180733	0.000153306	0.001431948	4.54554793	5.097694172	0.442158123	0.922293229
LINC02035	-2.50784288	7.80E-05	0.000813103	3.526893852	7.11884511	0.442158123	1.064075432
KIAA0319	-2.508113981	0.007299627	0.034135102	2.429881767	9.052119921	0.820610154	0.780511026
CENPF	-2.510539221	4.22E-07	8.89E-06	7.601510166	14.93982048	1.577514216	1.560313141
SPAG5	-2.515574487	3.44E-11	1.87E-09	15.35895276	14.41256371	2.145192263	1.98565975
MIR3142HG	-2.516155601	0.005981227	0.029279203	1.097795665	1.758401317	0.379082784	0.000708911
RASAL2	-2.522499496	2.26E-08	6.24E-07	9.090312281	22.49716747	1.82981557	2.552788561
COL6A1	-2.533232115	3.61E-14	3.86E-12	8.071658202	13.97318308	1.199062185	1.843877547
CDC20	-2.537038061	5.96E-09	1.97E-07	16.37760684	25.04557517	1.892890909	3.828828385
CELSR2	-2.537283524	0.002868847	0.016084045	1.803017719	2.109905828	0.252932107	0.284273317
MORN4	-2.53821394	0.005446778	0.027076286	1.332869683	2.988667105	0.5683088	0.000708911
C11orf80	-2.538381382	0.006374552	0.030776566	1.019437659	2.988667105	0.000630753	0.567837722
MSH5-SAPCD1	-2.543568578	2.08E-05	0.000259792	13.2432866	7.90973026	2.334418278	0.496946621
AKR1C2	-2.552439641	0.007402822	0.034528157	1.724659713	5.712827066	0.442158123	0.567837722
IL1B	-2.554544829	0.005132888	0.025809306	69.26926092	426.9031074	45.47794982	21.12625712
CD300LB	-2.556520802	8.14E-10	3.25E-08	6.504498081	9.843005071	1.135986847	1.064075432
KIF15	-2.559378794	0.003152101	0.017396806	3.683609864	3.340171616	0.252932107	0.709619925
UBE2C	-2.559892255	7.28E-11	3.70E-09	13.79179264	14.58831597	1.577514216	2.269224155
FLNB	-2.560479687	1.04E-11	6.35E-10	31.343986	84.44983754	6.434315281	9.21655209
XRRA1	-2.561474484	2.08E-06	3.61E-05	11.20597844	6.767340599	0.883685492	1.560313141
PDK1	-2.568277135	7.84E-07	1.54E-05	3.76196787	8.788491538	0.694459477	0.99318433
N4BP2L2-IT2	-2.570124147	0.001071798	0.007183983	3.918683882	3.076543233	0.442158123	0.496946621
FGFRL1	-2.572184292	2.55E-07	5.64E-06	9.717176329	32.16354152	2.082116924	3.54526398
GTSE1	-2.578790553	8.34E-05	0.000858466	6.269424063	9.49150056	0.631384138	1.48942204
B3GALNT1	-2.580635568	2.09E-05	0.00026066	3.37017784	5.712827066	0.5683088	0.638728823

ARHGAP32	-2.580964399	0.000996171	0.006768238	2.194807749	9.315748304	0.946760831	0.567837722
SLC9B1	-2.591311549	0.001568785	0.009762455	1.411227689	1.670525189	0.000630753	0.426055519
TFCP2L1	-2.60527032	0.001796784	0.010899283	0.862721647	2.197781955	0.379082784	0.000708911
C15orf52	-2.605633655	1.64E-06	2.91E-05	6.269424063	11.07327086	1.325212862	0.922293229
VASH1	-2.607105004	9.86E-18	2.23E-15	80.39609778	175.577382	17.85095155	15.45496901
EFHC1	-2.611731115	0.002728923	0.015425415	2.900029804	3.428047744	0.000630753	0.851402128
KIF18B	-2.622004918	1.37E-05	0.000184745	5.720918021	8.70061541	1.135986847	0.709619925
RECQL4	-2.624109369	8.19E-11	4.14E-09	19.27685307	11.77627988	2.019041586	1.98565975
STX1A	-2.625243405	4.24E-07	8.91E-06	9.638818323	29.35150543	2.397493617	2.623679662
MAOA	-2.632318087	2.99E-08	8.00E-07	6.974646118	15.11557274	1.009836169	1.843877547
KIFC3	-2.641129711	2.40E-17	4.74E-15	89.48562648	160.8141926	18.4186296	13.32823597
PIF1	-2.643034822	0.00193288	0.011564423	2.900029804	3.955304511	0.379082784	0.496946621
MT1X	-2.648008483	8.21E-11	4.14E-09	24.68355548	59.22938887	6.118938589	4.466848298
NOTCH3	-2.648421055	1.49E-08	4.33E-07	12.45970654	34.6240731	2.712870309	3.261699574
CDCA2	-2.651630726	0.0076241	0.035286526	3.135103822	2.285658083	0.000630753	0.709619925
TOP2A	-2.661559263	3.05E-09	1.08E-07	14.41865669	18.36698946	1.325212862	2.836352966
CYP27A1	-2.670505193	6.84E-22	3.37E-19	892.0283243	1515.776207	160.2750659	139.7270697
EME1	-2.67825964	0.001091426	0.007298598	3.056745816	3.340171616	0.5683088	0.213382215
APBA1	-2.678716535	1.63E-06	2.90E-05	4.623905936	14.32468759	1.009836169	1.347639837
KIF24	-2.689958186	0.000143141	0.001350732	4.780621949	3.955304511	0.379082784	0.709619925
WDR34	-2.692672258	3.98E-12	2.59E-10	16.53432285	14.41256371	1.955966247	1.843877547
SEMA3A	-2.693338033	0.002440857	0.014123497	1.411227689	1.846277444	0.252932107	0.142491114
BIRC5	-2.698036924	2.08E-11	1.20E-09	14.26194068	21.88203457	1.955966247	2.481897459
SEMA6B	-2.701719664	1.27E-27	1.83E-24	456.1227367	632.0059896	68.058921	64.72428447
E2F2	-2.738087895	3.43E-09	1.20E-07	9.168670287	9.930881199	1.072911508	1.205857634
TMEM71	-2.742420625	0.002146402	0.012632436	2.97838781	3.076543233	0.694459477	0.000708911
TICRR	-2.758463715	0.005651582	0.027940385	1.959733731	4.482561277	0.000630753	0.780511026

RBP1	-2.762958922	2.73E-05	0.000327207	2.586597779	5.976455449	0.5683088	0.426055519
CD276	-2.763867335	3.31E-35	1.19E-31	121.925841	139.7239219	18.04017757	12.40665165
C14orf79	-2.767622175	0.001193758	0.007854777	2.194807749	3.428047744	0.631384138	0.000708911
FAM111B	-2.770200087	8.51E-06	0.000122843	9.403744305	7.382473493	0.631384138	1.347639837
KANK1	-2.772312707	2.22E-08	6.14E-07	8.541806238	29.08787705	1.703664893	2.694570763
CDCA7	-2.775014089	0.004242531	0.022190636	2.351523761	1.758401317	0.000630753	0.496946621
FABP4	-2.813909275	1.01E-10	4.96E-09	15.75074279	25.04557517	2.334418278	2.269224155
TSEN2	-2.847773607	0.000900715	0.006204401	4.310473912	2.197781955	0.694459477	0.000708911
E2F1	-2.852063796	8.25E-12	5.15E-10	17.1611869	12.12778439	1.199062185	2.056550851
C16orf45	-2.856112931	2.65E-05	0.000318384	3.213461828	3.428047744	0.5683088	0.142491114
HAMP	-2.863861232	3.36E-07	7.26E-06	16.22089083	9.227872177	1.82981557	0.922293229
HTRA4	-2.864772016	5.48E-06	8.42E-05	2.194807749	5.800703194	0.442158123	0.426055519
TNFSF14	-2.879077539	1.85E-05	0.000235766	4.232115906	11.86415601	0.883685492	0.851402128
CASS4	-2.889096207	1.77E-06	3.12E-05	4.858979955	5.097694172	0.5683088	0.496946621
TRIP13	-2.891590711	0.000509635	0.003918243	5.094053973	7.11884511	0.000630753	1.347639837
IL1A	-2.898762196	0.00129349	0.008415848	1.489585695	2.285658083	0.189856769	0.213382215
PPP1R3G	-2.900194521	0.003267163	0.017872793	0.941079653	1.582649061	0.12678143	0.142491114
VNN2	-2.909144869	0.006300592	0.030511357	1.646301707	16.52159078	1.199062185	0.709619925
DNAAF4	-2.910694583	0.002948425	0.016434425	1.411227689	1.143268422	0.000630753	0.284273317
CAMKK1	-2.915524424	2.35E-16	3.53E-14	19.7470011	29.08787705	2.649794971	2.481897459
NCS1	-2.922651128	1.68E-13	1.55E-11	26.32907361	81.37417307	5.740486558	5.530214819
WEE1	-2.933343462	1.12E-05	0.000155684	3.76196787	3.955304511	0.379082784	0.426055519
KIF4A	-2.937119186	3.87E-05	0.000442969	3.056745816	6.591588344	0.5683088	0.426055519
FLRT2	-2.948908176	3.76E-05	0.000430644	3.213461828	7.206721238	0.505233461	0.567837722
SMOX	-2.956060426	1.06E-11	6.43E-10	22.01938328	68.72001067	4.605130464	4.679521602
CENPM	-2.957497611	1.07E-09	4.20E-08	13.40000261	11.16114699	1.766740232	0.709619925
LIF	-2.995597722	0.000212303	0.001876142	5.485844003	27.24247837	1.82981557	1.418530939

PKMYT1	-2.995701307	1.46E-12	1.09E-10	18.88506304	18.27911334	2.586719632	1.064075432
KIFC1	-3.002705973	4.93E-15	5.93E-13	19.98207512	21.0032733	2.27134294	1.772986445
WNT2B	-3.005070664	0.009933518	0.043697404	1.254511677	1.406896806	0.252932107	0.000708911
SV2B	-3.006515475	4.85E-06	7.58E-05	2.743313791	6.064331577	0.442158123	0.426055519
SERPINE1	-3.021639719	0.000118792	0.00114976	23.74325941	123.9062189	7.821972728	6.593581339
CDT1	-3.037381423	2.57E-12	1.75E-10	19.90371711	13.62167856	2.145192263	1.064075432
CEBPB-AS1	-3.050903076	0.004168346	0.021849967	2.508239773	3.076543233	0.252932107	0.284273317
RASAL1	-3.055761041	6.26E-10	2.57E-08	11.36269446	40.07239302	2.965171663	1.914768648
MKI67	-3.065932171	8.94E-20	3.00E-17	44.19469899	54.39620185	3.53284971	5.884670325
EGR1	-3.068309721	1.53E-19	4.85E-17	54.61631379	96.6646193	8.641952129	5.60110592
DIRAS1	-3.074352746	0.000233178	0.002036917	2.351523761	1.846277444	0.379082784	0.000708911
LAMB3	-3.07653659	1.44E-16	2.28E-14	31.50070201	74.69558736	4.289753772	5.742888123
EXO1	-3.077483986	0.000817033	0.005720817	2.900029804	2.900790978	0.000630753	0.567837722
VNN1	-3.078817754	1.51E-06	2.71E-05	2.900029804	7.733978005	0.631384138	0.355164418
TPST1	-3.080134684	2.20E-06	3.79E-05	4.075399894	11.5126515	0.883685492	0.567837722
DLGAP5	-3.089872458	1.91E-05	0.000242551	4.702263942	5.976455449	0.631384138	0.355164418
SLC28A3	-3.092516018	0.000728654	0.00525235	1.019437659	1.846277444	0.252932107	0.000708911
ZMIZ1-AS1	-3.095856904	9.55E-17	1.60E-14	24.13504944	47.36611163	2.839020986	3.828828385
C21orf58	-3.099682234	0.000786073	0.005568887	2.664955785	4.39468515	0.631384138	0.000708911
SHROOM1	-3.102433668	0.000137799	0.001307411	4.1537579	3.691676127	0.5683088	0.142491114
MYBL2	-3.128473282	3.84E-23	2.52E-20	24.9186295	25.04557517	2.334418278	2.198333054
MANEAL	-3.129381293	3.93E-08	1.03E-06	5.564202009	5.449198683	0.5683088	0.426055519
PLB1	-3.145829201	2.36E-13	2.09E-11	9.482102311	12.12778439	0.946760831	0.99318433
SLC2A5	-3.157050334	2.69E-25	2.43E-22	49.05289536	32.95442667	3.09132234	4.254174994
SPC24	-3.16293119	2.92E-08	7.85E-07	7.13136213	9.403624432	0.820610154	0.638728823
CBX7	-3.164497376	1.26E-08	3.76E-07	5.955992039	10.01875733	0.442158123	0.99318433
PIMREG	-3.195157454	0.000580463	0.004346995	2.664955785	4.131056766	0.5683088	0.000708911

PALLD	-3.208452049	0.000394952	0.003162907	14.4970147	69.4230197	1.82981557	5.459323717
RRM2	-3.225516228	7.01E-22	3.37E-19	33.45965216	36.11796727	2.334418278	3.616155081
DCHS1	-3.235860978	2.56E-05	0.000309177	4.467189924	3.428047744	0.379082784	0.284273317
CRIM1	-3.263672788	8.54E-07	1.65E-05	5.720918021	39.19363174	1.262137524	2.481897459
NDRG2	-3.269429093	1.70E-05	0.000221269	1.332869683	10.8975186	0.442158123	0.567837722
ASIC1	-3.273633226	2.96E-05	0.000349718	2.038091737	5.976455449	0.631384138	0.000708911
AURKB	-3.287686885	9.83E-12	6.01E-10	12.77313856	13.35805018	1.325212862	0.780511026
KCNN4	-3.311438509	1.83E-23	1.25E-20	79.69087572	194.0313689	10.97573965	10.91793852
IGSF3	-3.318142529	0.001648035	0.010172279	1.097795665	1.406896806	0.189856769	0.000708911
CACNA2D3	-3.318629405	1.18E-15	1.59E-13	16.06417482	14.0610592	1.325212862	1.064075432
PCBP3	-3.368350375	0.006216499	0.030208474	1.176153671	2.373534211	0.000630753	0.284273317
BUB1B	-3.376366487	5.77E-05	0.000623051	4.1537579	6.591588344	0.000630753	0.851402128
POU6F1	-3.395508828	2.57E-06	4.36E-05	3.213461828	4.834065788	0.12678143	0.496946621
RAD54L	-3.410761103	1.51E-07	3.50E-06	5.877634033	5.712827066	0.505233461	0.355164418
MT1G	-3.41726428	0.005199993	0.026092178	5.407485997	40.07239302	2.902096325	0.426055519
CLEC5A	-3.436866127	9.26E-08	2.26E-06	122.004199	343.9480428	10.21883559	24.24546558
SEMA4C	-3.440836487	1.51E-10	7.24E-09	10.89254642	17.66398044	0.694459477	1.418530939
ACOT11	-3.445863648	1.64E-18	4.22E-16	16.29924884	34.36044472	1.514438878	2.198333054
ZNRF3	-3.452483523	0.003710304	0.019823547	1.411227689	1.319020678	0.189856769	0.000708911
DPYSL3	-3.470510289	7.70E-05	0.000804205	1.332869683	27.41823062	1.199062185	0.851402128
BCAR1	-3.470958914	2.18E-07	4.89E-06	3.526893852	2.988667105	0.252932107	0.213382215
GPR27	-3.476760203	0.000387429	0.003114758	2.429881767	3.164419361	0.252932107	0.142491114
ACOX2	-3.510588921	0.000417324	0.003316202	1.803017719	3.076543233	0.000630753	0.355164418
CTNNAL1	-3.53561567	0.00030717	0.002568236	1.724659713	4.043180638	0.379082784	0.000708911
HTR7	-3.539871266	1.55E-08	4.47E-07	5.955992039	25.30920356	1.135986847	0.99318433
GJB2	-3.540068915	0.006370685	0.030768192	1.724659713	6.064331577	0.316007446	0.213382215
HJURP	-3.545115589	3.04E-09	1.08E-07	7.444794154	8.349110899	0.379082784	0.709619925

MARCO	-3.554173214	1.61E-15	2.13E-13	64.41106455	116.6125003	6.749691974	5.459323717
CIRBP-AS1	-3.558170859	0.001625355	0.010045181	1.019437659	1.934153572	0.189856769	0.000708911
SLC9A3-AS1	-3.564888712	0.000306594	0.002564905	1.567943701	7.206721238	0.5683088	0.000708911
CDC6	-3.597841161	1.13E-06	2.13E-05	4.388831918	3.867428383	0.000630753	0.567837722
SPC25	-3.600292981	0.000144988	0.001362711	2.97838781	3.252295489	0.000630753	0.426055519
PAQR5	-3.602863522	5.67E-10	2.35E-08	6.426140075	29.61513382	0.883685492	1.48942204
PCOLCE2	-3.621905092	4.65E-07	9.64E-06	3.918683882	30.23026671	1.135986847	1.064075432
FAM57A	-3.633645829	1.24E-05	0.000168524	2.821671798	4.39468515	0.442158123	0.000708911
TREML1	-3.642085049	0.000242705	0.002099804	1.019437659	3.252295489	0.000630753	0.284273317
MMP8	-3.655888606	0.009226274	0.041119321	0.470931616	3.691676127	0.252932107	0.000708911
ZNF554	-3.662259704	0.000554423	0.004195529	2.038091737	2.285658083	0.000630753	0.284273317
GAS6-AS1	-3.663139549	0.004836355	0.024590238	1.567943701	1.582649061	0.189856769	0.000708911
COL7A1	-3.664491513	7.34E-05	0.000774457	2.273165755	8.173358643	0.631384138	0.000708911
ACO1	-3.685589473	6.43E-16	9.10E-14	18.57163101	72.76231255	2.397493617	3.261699574
FOLR3	-3.754750314	0.000151949	0.001421111	26.1723576	3.515923872	0.694459477	1.064075432
MINCR	-3.759748514	0.006818524	0.032367801	0.941079653	1.319020678	0.12678143	0.000708911
LOC101928143	-3.771053316	0.000639171	0.004704474	0.941079653	3.6038	0.252932107	0.000708911
TXLNB	-3.783563275	1.99E-11	1.15E-09	7.444794154	8.788491538	0.442158123	0.496946621
AKR1C3	-3.834156882	2.77E-07	6.06E-06	5.564202009	9.930881199	0.5683088	0.284273317
SNRPN	-3.866354792	0.000545978	0.004142487	26.64250563	5.712827066	1.451363539	0.284273317
SORL1	-3.874571099	3.81E-30	9.16E-27	38.31784853	61.77779658	2.523644294	2.907244068
MARCKSL1	-3.920025691	1.95E-27	2.34E-24	60.33644823	139.7239219	5.046657834	5.459323717
CDCA3	-3.921269077	6.65E-06	9.98E-05	3.37017784	4.39468515	0.000630753	0.426055519
SLC46A2	-3.932565236	1.08E-06	2.07E-05	3.37017784	16.96097142	0.757534815	0.284273317
AKR1C1	-3.934317832	1.93E-11	1.13E-09	13.55671863	60.98691143	1.955966247	1.914768648
WNT5A	-3.940087988	6.13E-06	9.29E-05	3.135103822	12.39141278	0.379082784	0.426055519
LGI2	-3.964607424	1.11E-16	1.83E-14	26.01564159	63.53531914	0.946760831	3.687046183

PPBP	-4.024368356	0.000719629	0.005200287	88.78040442	1449.166102	47.18098396	27.50645625
TYMS	-4.02694207	1.34E-22	7.73E-20	18.72834702	21.0032733	1.009836169	0.922293229
RAI14	-4.029877448	1.35E-08	3.98E-07	2.821671798	21.9699107	0.5683088	0.638728823
ENO2	-4.093584462	4.67E-14	4.78E-12	6.269424063	23.99106164	0.631384138	0.780511026
ST18	-4.105775513	4.02E-05	0.0004574	1.489585695	4.39468515	0.000630753	0.284273317
SPTBN4	-4.114059305	0.005284878	0.026444548	0.862721647	2.0220297	0.12678143	0.000708911
MND1	-4.172401057	0.003056262	0.016969957	1.489585695	1.582649061	0.000630753	0.142491114
CDC45	-4.185020211	3.57E-11	1.91E-09	10.10896636	8.436987027	0.12678143	0.709619925
UHRF1	-4.185696702	1.72E-16	2.67E-14	13.00821258	17.8397327	0.379082784	0.99318433
PKIA	-4.214555705	0.007652703	0.035368276	0.549289622	0.000878761	0.000630753	0.000708911
ESCO2	-4.219334579	3.67E-05	0.000421643	2.664955785	3.691676127	0.000630753	0.284273317
SCG5	-4.262232733	1.49E-06	2.68E-05	1.097795665	5.361322555	0.252932107	0.000708911
FCAR	-4.395466008	3.40E-11	1.86E-09	5.955992039	40.24814528	0.757534815	0.99318433
OXER1	-4.542785567	5.05E-06	7.87E-05	2.586597779	5.273446427	0.12678143	0.142491114
MMP1	-4.548323727	0.00699487	0.033031075	0.706005634	0.000878761	0.000630753	0.000708911
MIR210HG	-4.559720103	1.11E-07	2.66E-06	2.900029804	11.24902312	0.000630753	0.496946621
NAT8L	-4.587137504	0.007988166	0.036654126	0.39257361	0.352383272	0.000630753	0.000708911
PCDHGC3	-4.608885435	0.004116629	0.0216577	0.39257361	24.69407066	0.189856769	0.638728823
STXBP1	-4.613485793	4.21E-06	6.67E-05	2.194807749	4.043180638	0.000630753	0.213382215
MLXIPL	-4.615551278	8.43E-14	8.16E-12	8.933596269	15.90645789	0.316007446	0.496946621
LINC01963	-4.651428007	0.000209853	0.001860841	0.941079653	3.340171616	0.000630753	0.142491114
SLIT3	-4.74511777	0.008739374	0.039411846	0.39257361	0.4402594	0.000630753	0.000708911
TMPRSS5	-4.804878624	0.009135603	0.040790806	0.00078358	0.879640039	0.000630753	0.000708911
CD1A	-4.822532263	0.007538353	0.035024462	0.862721647	0.000878761	0.000630753	0.000708911
AFAP1L1	-4.832657067	0.004106753	0.02161362	0.862721647	0.000878761	0.000630753	0.000708911
ADGRE4P	-4.840115068	4.63E-05	0.000516474	1.646301707	3.164419361	0.12678143	0.000708911
CXCL5	-4.854593524	4.11E-05	0.000467809	48.03424128	793.2586841	11.5434177	11.55595844

WWC1	-4.878798428	0.001460744	0.009241952	0.470931616	0.4402594	0.000630753	0.000708911
DBN1	-4.983142797	0.002699589	0.015289546	0.706005634	0.264507145	0.000630753	0.000708911
LINC02210	-4.98692953	0.007577076	0.035159277	0.627647628	0.352383272	0.000630753	0.000708911
ZBED3-AS1	-4.98877593	0.004403291	0.022849296	0.627647628	0.352383272	0.000630753	0.000708911
LINC01050	-5.007286563	0.002018064	0.011989579	0.470931616	0.528135528	0.000630753	0.000708911
VWCE	-5.029349788	0.006094214	0.029721393	0.314215604	0.703887783	0.000630753	0.000708911
GPRC5C	-5.050543569	0.006223895	0.030221074	0.157499592	0.879640039	0.000630753	0.000708911
NES	-5.070395745	0.005497513	0.027309679	0.00078358	1.055392294	0.000630753	0.000708911
PCLAF	-5.107245138	3.25E-10	1.45E-08	6.7395721	7.646101877	0.316007446	0.000708911
PDE4D	-5.12840348	0.003631911	0.01946958	0.470931616	0.616011656	0.000630753	0.000708911
PWAR6	-5.129322403	0.007191019	0.03373654	0.470931616	0.616011656	0.000630753	0.000708911
DNAJC3-AS1	-5.129632021	0.009825902	0.043336671	0.470931616	0.616011656	0.000630753	0.000708911
SLC25A48	-5.142550379	0.00188912	0.01134494	0.314215604	0.791763911	0.000630753	0.000708911
MYLK	-5.159832512	0.006450085	0.030969991	0.235857598	0.879640039	0.000630753	0.000708911
OCLN	-5.290464931	0.007461078	0.034740232	2.743313791	0.528135528	0.063706092	0.000708911
CXADR	-5.297183715	0.004664946	0.02386382	0.00078358	1.23114455	0.000630753	0.000708911
MEIKIN	-5.332205075	0.00081215	0.005699354	0.549289622	0.703887783	0.000630753	0.000708911
SORCS2	-5.361409074	0.00345364	0.018631583	0.314215604	0.967516167	0.000630753	0.000708911
PDGFRA	-5.397620229	0.003923098	0.020760627	0.00078358	1.319020678	0.000630753	0.000708911
CCL24	-5.452409521	0.001221571	0.008002041	0.314215604	1.055392294	0.000630753	0.000708911
ANO7	-5.467268862	0.004319468	0.022503336	0.235857598	1.143268422	0.000630753	0.000708911
STOX2	-5.494172357	0.005252203	0.026308412	0.00078358	1.406896806	0.000630753	0.000708911
CBARP	-5.530738487	0.002118006	0.012480581	0.470931616	0.967516167	0.000630753	0.000708911
PPFIA4	-5.536714018	0.001343233	0.008657523	0.39257361	1.055392294	0.000630753	0.000708911
CD244	-5.583890847	0.006716216	0.031955724	0.00078358	1.494772933	0.000630753	0.000708911
ADGRA3	-5.599607732	0.000736272	0.005291407	0.627647628	0.879640039	0.000630753	0.000708911
LOC105371998	-5.625663423	0.003740069	0.019960403	0.39257361	1.143268422	0.000630753	0.000708911

SEZ6L2	-5.638727462	0.000519185	0.003976836	1.176153671	0.352383272	0.000630753	0.000708911
MELTF-AS1	-5.690252298	0.002169826	0.012765088	0.549289622	1.055392294	0.000630753	0.000708911
L3MBTL1	-5.696137772	0.001865025	0.011228645	1.332869683	0.264507145	0.000630753	0.000708911
SMIM5	-5.715839691	0.005415951	0.026950895	1.097795665	0.528135528	0.000630753	0.000708911
SIT1	-5.718409861	0.001232978	0.008065777	1.097795665	0.528135528	0.000630753	0.000708911
SNRK-AS1	-5.755382366	0.0004035	0.003224203	3.291819834	1.23114455	0.063706092	0.000708911
MUC20	-5.757521086	1.62E-05	0.000212021	3.840325876	5.361322555	0.000630753	0.142491114
LGALS12	-5.810218249	0.001730589	0.010564353	1.724659713	0.000878761	0.000630753	0.000708911
NLGN3	-5.82700063	0.003295797	0.017954567	0.706005634	1.055392294	0.000630753	0.000708911
SHC3	-5.846179452	0.000209926	0.001860841	0.314215604	1.494772933	0.000630753	0.000708911
MROH7	-5.858295829	0.000812783	0.005699354	1.176153671	0.616011656	0.000630753	0.000708911
LOC101929523	-5.862499982	0.008317654	0.037930537	1.097795665	0.703887783	0.000630753	0.000708911
INCA1	-5.870086583	0.002287505	0.013346471	1.019437659	0.791763911	0.000630753	0.000708911
N4BP3	-5.889557045	0.003339944	0.01813346	0.00078358	1.846277444	0.000630753	0.000708911
RBFOX2	-5.889572818	0.000134943	0.001282006	0.706005634	1.143268422	0.000630753	0.000708911
GLB1L2	-5.907167597	0.000588238	0.004396096	0.549289622	1.319020678	0.000630753	0.000708911
AKAP3	-5.931585086	0.005135215	0.025812015	0.314215604	1.582649061	0.000630753	0.000708911
ADSSL1	-5.944376136	0.000743114	0.005329956	0.941079653	0.967516167	0.000630753	0.000708911
CCDC183-AS1	-5.95708182	0.001509478	0.009462763	0.78436364	1.143268422	0.000630753	0.000708911
LINC00663	-5.969591776	0.001412768	0.009013107	0.627647628	1.319020678	0.000630753	0.000708911
SNX22	-5.983148406	0.007110005	0.033418853	1.254511677	0.703887783	0.000630753	0.000708911
MYLK2	-6.023767514	0.007242051	0.033898812	0.00078358	2.0220297	0.000630753	0.000708911
SLC9A3	-6.041401382	0.006991803	0.0330274	0.549289622	1.494772933	0.000630753	0.000708911
PLOD2	-6.055339286	0.000677013	0.004939323	0.314215604	1.758401317	0.000630753	0.000708911
HSD11B1	-6.122790223	0.001006822	0.00682454	0.235857598	1.934153572	0.000630753	0.000708911
RPGRIP1L	-6.146168487	0.000541906	0.004122429	0.706005634	1.494772933	0.000630753	0.000708911
SLC29A2	-6.153992514	0.000212775	0.00187871	0.549289622	1.670525189	0.000630753	0.000708911

CCL20	-6.154901459	4.41E-05	0.0004966	0.941079653	10.8975186	0.12678143	0.000708911
SHANK3	-6.16470663	0.001586432	0.009859518	0.470931616	1.758401317	0.000630753	0.000708911
ZNF761	-6.204077416	3.38E-06	5.50E-05	8.30673222	4.218932894	0.000630753	0.142491114
SLC22A1	-6.22643575	6.83E-05	0.000728869	1.176153671	1.143268422	0.000630753	0.000708911
TMEFF1	-6.231635642	0.002988971	0.016638632	1.097795665	1.23114455	0.000630753	0.000708911
WDR31	-6.231642304	0.002945416	0.016423997	1.097795665	1.23114455	0.000630753	0.000708911
EMP2	-6.233515078	0.00015443	0.001438718	1.959733731	0.352383272	0.000630753	0.000708911
FAM198A	-6.257472989	0.000621827	0.004602446	0.706005634	1.670525189	0.000630753	0.000708911
LOC399715	-6.290094167	0.001433813	0.00910895	1.019437659	1.406896806	0.000630753	0.000708911
LINC02201	-6.321454635	0.000193403	0.001741106	1.332869683	1.143268422	0.000630753	0.000708911
KCNAB3	-6.456201117	0.000352023	0.00288639	0.706005634	2.0220297	0.000630753	0.000708911
LINC01569	-6.55112959	7.88E-05	0.000819829	1.411227689	1.494772933	0.000630753	0.000708911
P2RX5	-6.551317187	0.000419806	0.003330519	1.411227689	1.494772933	0.000630753	0.000708911
SPRY4	-6.555764591	2.83E-05	0.00033599	0.314215604	2.637162594	0.000630753	0.000708911
FHAD1	-6.599693492	0.000132273	0.001262451	2.116449743	0.879640039	0.000630753	0.000708911
LRRC32	-6.620707261	0.004286846	0.022381866	0.941079653	2.109905828	0.000630753	0.000708911
PLXNB3	-6.681088091	2.06E-05	0.000257783	1.254511677	1.934153572	0.000630753	0.000708911
CELSR3	-6.699950885	0.000285901	0.002421266	0.941079653	2.285658083	0.000630753	0.000708911
DOCK3	-6.714204186	2.91E-05	0.000344762	1.411227689	1.846277444	0.000630753	0.000708911
CCDC150	-6.748988958	0.000873885	0.006045556	2.273165755	1.055392294	0.000630753	0.000708911
CEACAM19	-6.789700682	2.83E-05	0.00033599	1.411227689	2.0220297	0.000630753	0.000708911
CEACAM21	-6.820952473	0.001844864	0.01112546	3.056745816	0.4402594	0.000630753	0.000708911
OBSL1	-6.853157838	1.38E-05	0.000186145	2.508239773	1.055392294	0.000630753	0.000708911
ST3GAL6-AS1	-6.932319207	1.56E-05	0.000205205	1.332869683	2.461410339	0.000630753	0.000708911
WFDC21P	-7.146028535	8.86E-05	0.000897535	0.706005634	3.691676127	0.000630753	0.000708911
GFRA2	-7.283417868	0.00034836	0.002859602	0.00078358	4.834065788	0.000630753	0.000708911
NEK10	-7.386555863	3.43E-05	0.000396917	1.411227689	3.779552255	0.000630753	0.000708911

G0S2	-7.661034643	1.94E-17	3.94E-15	18.02312497	50.70540448	0.000630753	0.284273317
TMX2-CTNND1	-7.783935568	2.71E-05	0.000325707	3.135103822	3.691676127	0.000630753	0.000708911
STXBP5-AS1	-7.797474283	3.71E-06	5.99E-05	1.019437659	5.888579322	0.000630753	0.000708911
TNC	-7.879208751	0.007095291	0.033400875	0.00078358	7.294597366	0.000630753	0.000708911
NPHP3	-8.025951211	1.58E-07	3.63E-06	3.683609864	4.39468515	0.000630753	0.000708911
CNIH3	-8.273370338	2.56E-05	0.000308823	0.627647628	8.964243793	0.000630753	0.000708911
MROH7-TTC4	-8.540472259	4.86E-08	1.25E-06	4.075399894	7.470349621	0.000630753	0.000708911
CLCN4	-9.057531732	1.45E-08	4.23E-07	5.877634033	10.63389022	0.000630753	0.000708911
LOC400927-CSNK1E	-22.03867378	4.11E-06	6.55E-05	0.00078358	30.84539961	0.000630753	0.000708911
SERF2-C15ORF63	-22.36872074	2.94E-06	4.91E-05	0.00078358	40.24814528	0.000630753	0.000708911
SENP3-EIF4A1	-23.10962353	1.37E-06	2.50E-05	0.00078358	70.56540936	0.000630753	0.000708911
